# Spirostane-Type Saponins Obtained from *Yucca schidigera*

**DOI:** 10.3390/molecules23010167

**Published:** 2018-01-14

**Authors:** Lu Qu, Jianli Wang, Jingya Ruan, Xiaoyong Yao, Peijian Huang, Yue Wang, Haiyang Yu, Lifeng Han, Yi Zhang, Tao Wang

**Affiliations:** 1 Tianjin State Key Laboratory of Modern Chinese Medicine, 312 Anshanxi Road, Nankai District, Tianjin 300193, China; qululuhan88@163.com (L.Q.); Ruanjy19930919@163.com (J.R.); 2 Tianjin Key Laboratory of TCM Chemistry and Analysis, Institute of Traditional Chinese Medicine, Tianjin University of Traditional Chinese Medicine, 312 Anshanxi Road, Nankai District, Tianjin 300193, China; wjl15802226160@126.com (J.W.); hpjforever@sina.com (P.H.); wy1609112949@163.com (Y.W.); yuhaiyang19830116@hotmail.com (H.Y.); hanlifeng_1@sohu.com (L.H.); 3 Risun Bio-Tech Inc., D/17F, Haibo Business Building, FengCheng 9th Road, Xi’an 710018, China; denny@risunextract.com

**Keywords:** *Yucca schidigera*, spirostane-type saponin, Yucca spirostanoside

## Abstract

It is well known that spirostane-type saponins show various bioactivities. In our on-going program of screening these kinds of constituents from natural products, *Yucca schidigera* was found to be rich in them, and nine new spirostanol saponins, Yucca spirostanosides A_1_ (**1**), A_2_ (**2**), B_1_ (**3**), B_2_ (**4**), B_3_ (**5**), C_1_ (**6**), C_2_ (**7**), C_3_ (**8**), and D_1_ (**9**), together with five known ones (**10**–**14**) were isolated from the plant. Their structures were elucidated by extensive spectroscopic methods, including 1D and 2D NMR and MS spectra, and comparing with published data.

## 1. Introduction

As one of the secondary metabolites, spirostanol saponins have been found to have broad bioactivities, such as antiproliferative, anti-inflammatory [1,2,3,4], anti-HIV [5], anti-bacterial [6], anti-fungi [7], and anti-hyperuricemic [8] activities, which make the phytochemical or bioactive researches for spirostanol saponins meaningful.

In our on-going program of investigating spirostanol saponins [3,4,8] from natural products, we found that *Yucca schidigera* Roezl (Agavaceae family) is a plant rich in these kinds of constituents. As one of the major industrial sources of steroid saponins, *Y. schidigera* is native to the desert of the southwestern United States and northern Baja California, Mexico [9]. The commercial extracts of *Y. schidigera* are approved by the FDA as GRAS (Generally Recognized as Safe) and widely used as animal and human food additives [10].

Then, the isolation of spirostanol saponins from *Y. schidigera* stems was studied, which led to the separation of nine new spirostane-type saponins, Yucca spirostanosides A_1_ (**1**), A_2_ (**2**), B_1_ (**3**), B_2_ (**4**), B_3_ (**5**), C_1_ (**6**), C_2_ (**7**), C_3_ (**8**), and D_1_ (**9**), along with five known ones, schidigera-saponins A3 (**10**) and A1 (**11**) [9], 5β-spirost-25(27)-en-3β-ol-12-one 3-*O*-{β-d-glucopyranosyl-(1→2)-*O*-[β-d-glucopyranosyl-(1→3)]-β-d-glucopyranoside} (**12**) [11], schidigera-saponins C2 (**13**) and C1 (**14**) [9]. In this paper, their structures were determined by analysis of physical data, spectroscopic analysis, and chemical methods.

## 2. Results and Discussion

**Figure 1:**
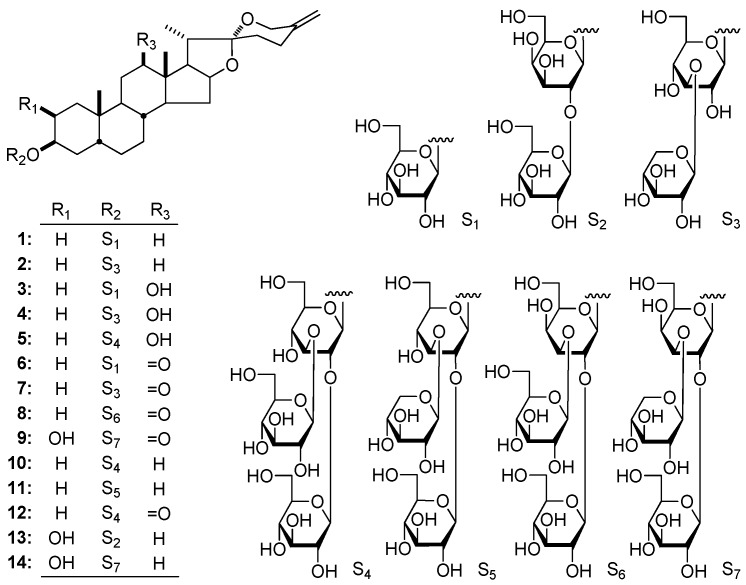
The compounds **1**–**14** obtained from the stems of *Y. schidigera*.

The 70% EtOH extract of *Y. schidigera* stems were subjected to D101 column chromatography (CC) (H_2_O → 95% EtOH), and 95% EtOH eluate was yielded, which was separated by Silica gel, ODS, and finally preparative HPLC to afford compounds **1**–**14**. The structures of them are shown in Figure 1.

**Figure 2:**
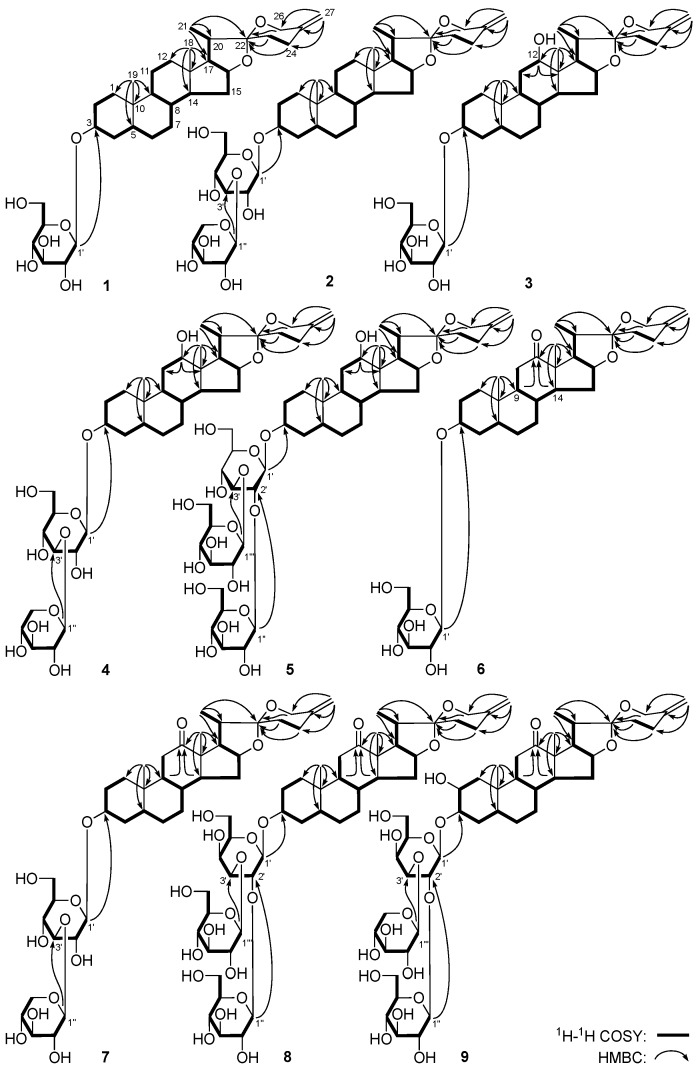
The main ^1^H–^1^H COSY and HMBC correlations of **1**–**9**.

*Yucca spirostanoside A*_1_ (**1**) was obtained as a white powder with negative optical rotation (${\left[\alpha \right]}_{D}^{25}$ −45.4°, MeOH). The molecular formula, C_33_H_52_O_8_, of **1** was established by positive-ion HRESI-TOF-MS (*m/z* 577.3758 [M + H]^+^, calcd for C_33_H_53_O_8_, 577.3735). The IR spectrum showed absorption bands ascribable to hydroxyl (3370 cm^−1^), terminal olefinic bond (1651, 1019, 921 cm^−1^), and *O*-glycosidic linkage (1077 cm^−1^). Acid hydrolysis of it yielded d-glucose, which was identified by retention time and optical rotation using chiral detection by HPLC analysis [12]. Thirty-three carbon signals were displayed in the ^13^C NMR (Table 1, C_5_D_5_N) spectrum. In addition to the carbon signals represented by d-glucose, the other 27 indicated **1** was a spirostane-type steroid saponin. Its ^1^H NMR spectrum showed signals for two tertiary methyl groups at δ 0.84, 0.85 (3H each, both s, H_3_-18, 19), a secondary methyl group at δ 1.11 (3H, d, *J* = 7.0 Hz, H_3_-21), one oxygenated methylene group at δ 4.04, 4.48 (1H each, both d, *J* = 12.5 Hz, H_2_-26), two oxygenated methine protons at δ [4.37 (1H, m, H-3), 4.61 (1H, q like, ca. *J* = 8 Hz, H-16)], one terminal olefinic moiety at δ 4.79, 4.82 (1H each, both br. s, H_2_-27), together with the sugar portion signal of one anomeric proton at δ 4.93 (1H, d, *J* = 8.0 Hz, H-1′). The ^1^H–^1^H COSY spectrum of **1** suggested the presence of three partial structures written in bold lines as shown in Figure 2. The planar structure of the aglycon was determined based on the key HMBC correlations from H_3_-18 to C-12-14, C-17; H_3_-19 to C-1, C-5, C-9, C-10; H_3_-21 to C-17, C-20, C-22; H_2_-26 to C-22; H_2_-27 to C-24-26, which was very close to that of 5β-spirost-25(27)-en-3β-ol 3-*O*-β-d-glucopyranosyl(1→3)-[β-d-glucopyranosyl(1→2)]-β-d-glucopyranoside [9]. Thus, the aglycon of **1** was determined to be 5β-spirost-25(27)-en-3β-ol. Meanwhile, the long-range correlation from H-1′ to C-3 observed in the HMBC spectrum suggested d-glucose was attached to C-3 of the aglycon. On the basis of above mentioned evidence, the structure of Yucca spirostanoside A_1_ (**1**) was identified as 5β-spirost-25(27)-en-3β-ol 3-*O*-β-d-glucopyranoside.

The molecular formula of Yucca spirostanoside A_2_ (**2**) was assigned as C_38_H_60_O_12_ on the basis of ^13^C NMR data and positive-ion HRESI-TOF-MS (*m/z* 709.4178 [M + H]^+^, calcd for C_38_H_61_O_12_, 709.4158). A detailed comparison between compounds **2** and **1** indicated that they have the consistent ^1^H and ^13^C NMR spectroscopic data from their aglycon moieties (Table 1), except for the signals due to the sugar moieties. Meanwhile, its ^1^H NMR spectrum suggested the presence of two anomeric proton signals at δ 4.91 (1H, d, *J* = 7.5 Hz, H-1′) and 5.26 (1H, d, *J* = 8.0 Hz, H-1′′), which correlated to the corresponding anomeric carbon signals at δ_C_ 102.5 (C-1′) and 106.3 (C-1′′), respectively. With the help of ^1^H–^1^H COSY, HSQC, and HMBC NMR analysis, the ^1^H and ^13^C NMR chemical shifts for the sugar moiety were assignable. On acid hydrolysis, **2** yielded d-glucose and d-xylose [12]. Furthermore, the sugar sequence was consolidated by key HMBC correlations from δ_H_ 4.91 (H-1′) to δ_C_ 74.4 (C-3); δ_H_ 5.26 (H-1′′) to δ_C_ 87.8 (C-3′). Consequently, the structure of **2** was elucidated to be 5β-spirost-25(27)-en-3β-ol 3-*O*-β-d-xylopyranosyl(1→3)-β-d-glucopyranoside.

*Yucca spirostanoside B*_1_ (**3**) was determined to possess the molecular formula, C_33_H_52_O_9_ by its quasi-molecular ion peak at *m/z* 593.3700 [M + H]^+^ (calcd for C_33_H_53_O_9_, 593.3684) in the positive HRESI-TOF-MS experiment, which was 16 amu greater than that of **1**. Moreover, the ^13^C NMR signals of **3** were coincident with those of **1** except for the C ring carbons. Furthermore, comparing the DEPT spectrum of compound **3** with that of **1**, showed that **3** had one oxygenated methine more and one methylene less than **1**. According to the HMBC correlations from δ_H_ 1.09 (H_3_-18) to δ_C_ 79.4 (C-12), δ_H_ 3.54 (H-12) to δ_C_ 31.4 (C-11), 46.7 (C-13), the position of the oxygenated methine was determined. Meanwhile, the configuration of C-12 hydroxyl group in **3** was deduced to be β by comparing carbon signals of C-11–14, 17, and 18 (δ_C_ 11.2 (C-18), 31.4 (C-11), 46.7 (C-13), 55.3 (C-14), 63.0 (C-17), 79.4 (C-12)) with those of its similar compounds, (25*R*)-26-*O*-β-d-glucopyranosyl-5β-furostane-3β,12β,22,26-tetraol-3-*O*-β-d-glucopyranosyl(1→2)-β-d-galactopyranoside (δ_C_ 11.3 (C-18), 31.4 (C-11), 47.0 (C-13), 55.2 (C-14), 63.8 (C-17), 79.6 (C-12)) [13] and (25*R*)-26-*O*-β-d-glucopyranosyl-5β-furostane-3β,12α,22,26-tetraol-3-*O*-β-d-glucopyranosyl(1→2)-β-d-galactopyranoside (δ_C_ 17.5 (C-18), 29.6 (C-11), 45.8 (C-13), 48.6 (C-14), 54.6 (C-17), 71.7 (C-12)) [14]. Finally, the β-d-glucopyranosyl was proved to link at C-3 of aglycon by the observed HMBC correlation from δ_H_ 4.93 (H-1′) to δ_C_ 74.3 (C-3). Thus, the structure of **3** was determined to be 5β-spirost-25(27)-en-3β,12β-diol 3-*O*-β-d-glucopyranoside.

*Yucca spirostanoside B*_2_ (**4**) was isolated as white powder with negative optical rotation (${\left[\alpha \right]}_{D}^{25}$ –36.2°, MeOH). The molecular formula, C_38_H_60_O_13_ of **4** was deduced by the positive-ion HRESI-TOF-MS signal at *m/z* 725.4121 [M + H]^+^ (calcd for C_38_H_61_O_13_, 725.4107). The ^1^H and ^13^C NMR (Table 1) spectroscopic data analysis indicated that **4** had the same aglycon, 5β-spirost-25(27)-en-3β,12β-diol as **3**, and the same sugar moiety, β-d-xylopyranosyl(1→3)-β-d-glucopyranosyl as **2**, which was supported by ^1^H–^1^H COSY, HSQC, and HMBC experiments (Figure 2). Moreover, the HMBC correlation from δ_H_ 4.91 (H-1′) to δ_C_ 74.4 (C-3) suggested β-d-xylopyranosyl(1→3)-β-d-glucopyranosyl was attached to C-3 of the aglycon. Therefore, the structure of **4** was established as 5β-spirost-25(27)-en-3β, 12β-diol 3-*O*-β-d-xylopyranosyl(1→3)-β-d-glucopyranoside.

The HRESI-MS of Yucca spirostanoside B_3_ (**5**) showed the [M + Na]^+^ ion at *m/z* 939.4565 (calcd for C_45_H_72_O_19_Na, 939.4560), consistent with the molecular formula of C_45_H_72_O_19_. The ^1^H and ^13^C NMR spectroscopic data comparison of **5**, **4** and **3** revealed that all the three compounds have the same aglycon pattern and the difference was only in the signals due to the sugar moieties. Compound **5** was subjected to acid hydrolysis and give d-glucose only. The ^1^H NMR spectrum of **5** indicated there were three β-d-glucopyranosyl moieties [δ 4.87 (1H, d, *J* = 7.5 Hz, H-1′), 5.35 (1H, d, *J* = 7.5 Hz, H-1′′′), 5.65 (1H, d, *J* = 7.5 Hz, H-1′′)]. A combination of HSQC, HSQC-TOCSY, and ^1^H–^1^H COSY spectra analysis led to the assignment of three β-d-glucopyranosyl units. In the HSQC-TOCSY spectrum, the correlations between the following proton and carbon pairs were observed: δ_H_ 4.87 (H-1′) and δ_C_ 70.1 (C-4′), 78.0 (C-5′), 80.1 (C-2′), 88.4 (C-3′), 102.1 (C-1′); δ_H_ 3.79 (H-5′) and δ_C_ 62.6 (C-6′); δ_H_ 5.65 (H-1′′) and δ_C_ 72.6 (C-4′′), 76.6 (C-2′′), 78.4 (C-3′′), 104.4 (C-1′′); δ_H_ 3.97 (H-5′′) and δ_C_ 63.5 (C-6′′), 72.6 (C-4′′), 78.4 (C-3′′ and 5′′); δ_C_ 104.4 (C-1′′) and δ_H_ 4.28 (H-3′′); δ_H_ 5.35 (H-1′′′) and δ_C_ 71.8 (C-4′′′), 75.5 (C-2′′′), 78.7 (C-3′′′), 105.0 (C-1′′′); δ_C_ 105.0 (C-1′′′) and δ_H_ 4.22 (H-3′′′). Once again, direct evidence of the sugar sequence and the linkage sites was derived from HSQC-TOCSY and HMBC experiments. The glycosidation shifts on C-3 (δ 75.7), C-2′ (δ 80.1), and C-3′ (δ 88.4) suggested the linkage sites. Long-range correlations from δ_H_ 4.87 (H-1′) to δ_C_ 75.7 (C-3); δ_H_ 5.65 (H-1′′) to δ_C_ 80.1 (H-2′); δ_H_ 5.35 (H-1′′′) to δ_C_ 88.4 (H-3′) were observed in the HMBC spectrum of it. Therefore, compound **5** was established as 5β-spirost-25(27)-en-3β, 12β-diol 3-*O*-β-d-glucopyranosyl(1→3)-[β-d-glucopyranosyl(1→2)]-β-d-glucopyranoside.

*Yucca spirostanoside C*_1_ (**6**) presented as a white powder with negative optical rotation (${\left[\alpha \right]}_{D}^{25}$ −8.9°, MeOH). The positive-ion HRESI-TOF-MS spectrum of **6** (*m/z* 591.3557 [M + H]^+^, calcd for C_33_H_51_O_9_, 591.3528) supported a molecular formula of C_33_H_50_O_9_, two proton less than that of **3**. Meanwhile, the ^13^C NMR spectrum of **6** was similar to that of **3**, except for the signals due to the C-ring carbons of aglycon moiety. The above-mentioned evidence suggested the hydroxyl at C-12 in **3** changed into carboxyl in **6**, which was identified by the presence of carbon signal at δ_C_ 213.0 (C-12) and the long-range correlations from δ_H_ 1.10 (H_3_-18), 1.49 (H-14), 1.76 (H-9), 2.21, 2.38 (H_2_-11), 2.83 (H-17) to δ_C_ 213.0 (C-12) showed in HMBC spectrum of **6**. On the other hand, the ^1^H, ^13^C NMR (Table 1, C_5_D_5_N) and 2D NMR (^1^H–^1^H COSY, HSQC, HMBC) experiments suggested the aglycon moiety of **6** was identical with that of 5β-spirost-25(27)-en-3β-ol-12-one 3-*O*-β-d-xylopyranosyl(1→3)-[β-d-glucopyranosyl (1→2)]-β-d-glucopyranoside [9], which indicated the aglycon of **6** was 5β-spirost-25(27)-en-3β-ol-12-one. On acid hydrolysis, **6** afforded glucose [12]. Finally, the linkage position of β-d-glucopyranosyl was identified by the HMBC correlation from δ_H_ 4.94 (H-1′) to δ_C_ 73.9 (C-3). Then, **6** was formulated as 5β-spirost-25(27)-en-3β-ol-12-one 3-*O*-β-d-glucopyranoside.

The molecular formula of Yucca spirostanoside C_2_ (**7**) was established as C_38_H_58_O_13_ by positive-ion HRESI-TOF-MS analysis (*m/z* 723.3959 [M + H]^+^, calcd for C_38_H_59_O_13_, 723.3950). The ^1^H and ^13^C NMR (Table 1) spectroscopic data for aglycon of **7** were identical to those of **6**. The remaining eleven carbon signals were assigned to the sugar moiety, which was determined to be β-d-xylopyranosyl(1→3)-β-d-glucopyranosyl group by comparing the NMR data of it with those of compounds **2** and **4**. Finally, according to the ^1^H–^1^H COSY, HSQC, and HMBC experiments, the structure of **7** was identified as 5β-spirost-25(27)-en-3β-ol-12-one 3-*O*-β-d-xylopyranosyl(1→3)-β-d-glucopyranoside.

*Yucca spirostanoside C*_3_ (**8**) was obtained as white powder, and its molecular formula was deduced as C_45_H_70_O_19_ from [M + Na]^+^ quasi-molecular ion at *m/z* 937.4412 (calcd for C_45_H_70_O_19_Na, 937.4404 ) in the positive-ion HRESI-TOF-MS spectrum. On acid hydrolysis, **8** gave d-glucose and d-galactose as sugar components. The ^1^H and ^13^C NMR (Table 1) spectra indicated that **8** possessed the same aglycon, 5β-spirost-25(27)-en-3β-ol-12-one as that of **6** and **7**. The presence of one β-d-galactopyranosyl (δ 4.83 (1H, d, *J* = 7.5 Hz, H-1′)) and two β-d-glucopyranosyl (δ 5.37 (1H, d, *J* = 7.5 Hz, H-1′′′), 5.57 (1H, d, *J* = 7.5 Hz, H-1′′)) were suggested by its ^1^H and ^13^C NMR spectra, too. The linkage positions between sugar and sugar, as well as aglycon and sugar were elucidated by HMBC correlations from δ_H_ 4.83 (H-1′) to δ_C_ 75.0 (C-3); δ_H_ 5.57 (H-1′′) to δ_C_ 77.8 (C-2′); δ_H_ 5.37 (H-1′′′) to δ_C_ 84.3 (C-3′′). The combined use of ^1^H–^1^H COSY, HSQC, and HSQC-TOCSY experiments allowed the sequential assignments of all resonances for each monosaccharide. In the HSQC-TOCSY spectrum of **8**, the correlations between δ_H_ 4.83 (H-1′) and δ_C_ 70.0 (C-4′), 77.8 (C-2′), 84.3 (C-3′), 102.0 (C-1′); δ_H_ 3.98 (H-5′) and δ_C_ 62.5 (C-6′), 70.0 (C-4′), 76.6 (C-5′); δ_H_ 5.57 (H-1′′) and δ_C_ 72.9 (C-4′′), 76.5 (C-2′′), 78.5 (C-3′′), 104.6 (C-1′′); δ_H_ 3.81 (H-5′′) and δ_C_ 63.6 (C-6′′), 72.9 (C-4′′), 76.5 (C-2′′), 78.1 (C-5′′), 78.5 (C-3′′); δ_H_ 5.37 (H-1′′′) and δ_C_ 71.7 (C-4′′′), 75.5 (C-2′′′), 78.7 (C-3′′′), 105.5 (C-1′′′); δ_H_ 3.92 (H-5′′′) and δ_C_ 62.7 (C-6′′′), 71.7 (C-4′′′), 78.5 (C-5′′′), 78.7 (C-3′′′) were found. On the basis of above mentioned evidence, the structure of Yucca spirostanoside C_3_ (**8**) was formulated as 5β-spirost-25(27)-en-3β-ol-12-one 3-*O*-β-d-glucopyranosyl(1→3)-[β-d-glucopyranosyl(1→2)]-β-d-galactopyranoside.

The molecular formula of Yucca spirostanoside D_1_ (**9**) was elucidated as C_44_H_68_O_19_ from [M + Na]^+^ quasi-molecular ion at *m/z* 923.4293 (calcd for C_44_H_68_O_19_Na, 923.4247) in the positive-ion HRESI-TOF-MS spectrum. Acid hydrolysis of it yielded d-galactose, d-glucose, and d-xylose [12]. The ^1^H and ^13^C NMR (Table 1) suggested the presence of one β-d-galactopyranosyl [δ 4.98 (1H, d, *J* = 7.5 Hz, H-1′)], one β-d-glucopyranosyl (δ 5.57 (1H, d, *J* = 8.0 Hz, H-1′′)], together with one β-d-xylopyranosyl (δ 5.22 (1H, d, *J* = 7.5 Hz, H-1′′′)). The ^13^C NMR signals due to the sugar moieties of **9** were in good agreement with those of schidigera-saponin A_2_, which was 5β-spirost-25(27)-en-3β-ol 3-*O*-β-d-xylopyranosyl(1→3)[β-d-glucopyranosyl(1→2)]-β-d-galactopyranoside [9]. To assign the badly overlapped protons in sugar chemical shift range, HSQC-TOCSY and ^1^H−^1^H COSY experiments were determined. In the HSQC-TOCSY spectrum, the correlations between the following proton and carbon pairs were observed: δ_H_ 4.98 (H-1′) and δ_C_ 69.7 (C-4′), 77.1 (C-2′), 84.1 (C-3′), 101.6 (C-1′); δ_H_ 5.57 (H-1′′) and δ_C_ 72.6 (C-4′′), 76.2 (C-2′′), 78.5 (C-3′′), 104.2 (C-1′′); δ_H_ 3.77 (H-5′′) and δ_C_ 63.3 (C-6′′), 72.6 (C-4′′), 77.8 (C-5′′); δ_H_ 5.22 (H-1′′′) and δ_C_ 67.0 (C-5′′′), 70.9 (C-4′′′), 75.0 (C-2′′′), 78.3 (C-3′′′), 106.0 (C-1′′′); δ_H_ 4.38 (H-5′) and δ_C_ 61.8 (C-6′), 76.6 (C-5′). Meanwhile, the correlations between δ_H_ 4.11 (H-5′) and δ_H_ 4.39 (H_2_-6′), 4.74 (H-4′) were found in the ^1^H–^1^H COSY spectrum. Meanwhile, the ^13^C NMR data for aglycon of **9** were almost superimposable on those of **6** except for the signals due to the A-ring carbons. Moreover, comparing the ^13^C NMR data of C-1–6 and C-10 of **9** [δ_C_ 26.0 (C-6), 30.5 (C-4), 35.3 (C-5), 37.3 (C-10), 39.6 (C-1), 66.7 (C-2), 79.7 (C-3)] with those of 3-*O*-β-d-xylopyranosyl(1→3)[β-d-glucopyranosyl(1→2)]-β-d-galactopyranosyl-5β-spirost-25(27)-ene-2β,3β-diol (schidigera-saponin C1) [δ_C_ 26.4 (C-6), 31.8 (C-4), 36.1 (C-5), 37.1 (C-10), 40.3 (C-1), 67.1 (C-2), 81.6 (C-3)], which obtained *Y. schidigera* [9], the aglycon of **9** was clarified as 5β-spirost-25(27)-en-2β,3β-diol-12-one. Finally, in the HMBC experiment (Figure 2), long-range correlations were observed from δ_H_ 4.98 (H-1′) to δ_C_ 79.7 (C-3); δ_H_ 5.57 (H-1′′) to δ_C_ 77.1 (C-2′); δ_H_ 5.22 (H-1′′′) to δ_C_ 84.1 (C-3′), then the connectivities between oligoglycoside moieties and aglycon were characterized. Thus, the structure of Yucca spirostanoside D_1_ (**9**) was elucidated to be 5β-spirost-25(27)-en-2β,3β-diol-12-one 3-*O*-β-d-xylopyranosyl(1→3)-[β-d-glucopyranosyl(1→2)]-β-d-galactopyranoside.

The structures of known compounds **10**–**14** were identified by comparing their ^1^H, ^13^C NMR data with references.

## 3. Experimental

### 3.1. General

Optical rotations were measured on a Rudolph Autopol^®^ IV automatic polarimeter (l = 50 mm) (Rudolph Research Analytical, Hackettstown, NJ, USA). IR spectra were recorded on a Varian 640-IR FT-IR spectrophotometer (Varian Australia Pty Ltd., Mulgrave, Australia). NMR spectra were determined on a Bruker 500 MHz NMR spectrometer (Bruker BioSpin AG Industriestrasse 26 CH-8117, Fällanden, Switzerland) at 500 MHz for ^1^H and 125 MHz for ^13^C NMR (internal standard: TMS). Positive-ion mode HRESI-TOF-MS were obtained on an Agilent Technologies 6520 Accurate-Mass Q-Tof LC/MS spectrometer (Agilent Corp., Santa Clara, CA, USA).

Column chromatographies (CC) were performed on macroporous resin D101 (Haiguang Chemical Co., Ltd., Tianjin, China), Silica gel (48–75 μm, Qingdao Haiyang Chemical Co., Ltd., Qingdao, China), and ODS (40–63 μm, YMC Co., Ltd., Tokyo, Japan). Preparative high-performance liquid chromatography (PHPLC) columns, Cosmosil 5C_18_-MS-II (20 mm i.d. × 250 mm, Nacalai Tesque, Inc., Kyoto, Japan), Wacopak Navi C_30_-5 (7.5 mm i.d. × 250 mm, Wako Pure Chemical Industries, Ltd., Osaka, Japan), and Cosmosil PBr (20 mm i.d. × 250 mm, Nacalai Tesque, Inc., Kyoto, Japan) were used to separate the constituents.

### 3.2. Plant Material

The stems of *Y. schidigera* were collected from the State of Florida, the United States of America, and identified by Dr. Li Tianxiang (The Hall of TCM Specimens, Tianjin University of TCM, China). The voucher specimen was deposited at the Academy of Traditional Chinese Medicine of Tianjin University of TCM (No. 20160301).

### 3.3. Extraction and Isolation

The dried stems of *Y. schidigera* (5.0 kg) were refluxed with 70% ethanol-water for three times. Evaporation of the solvent under pressure provided a 70% ethanol-water (800.0 g). The residue (700.0 g) was dissolved in H_2_O, and subjected to D101 CC (H_2_O → 95% EtOH) to afford H_2_O (380.4 g) and 95% EtOH (310.1 g) eluates, respectively.

The 95% EtOH eluate (200.0 g) was subjected to silica gel CC (CH_2_Cl_2_ → CH_2_Cl_2_–MeOH (100:1 → 100:3 → 100:7 → 5:1 → 3:1 → 2:1, *v*/*v*) → MeOH) to afford 13 fractions (Fr. 1–Fr. 13). Fraction 6 (12.0 g) was separated by ODS CC [MeOH–H_2_O (30:70 → 40:60 → 50:50 → 60:40 → 70:30 → 80:20 → 100:0, *v*/*v*)], and fourteen fractions (Fr. 6-1–Fr. 6-14) were obtained. Fraction 6-11 (596.5 mg) was purified by PHPLC (CH_3_CN–1% CH_3_COOH (40:60, *v*/*v*), Cosmosil 5C_18_-MS-II column) to give Yucca spirostanosides B_1_ (**3**, 12.5 mg, *t*_R_ 38.76′) and B_2_ (**4**, 15.6 mg, *t*_R_ 29.24′). Fraction 6-12 (800.9 mg) was isolated by PHPLC (MeOH–1% CH_3_COOH (75:25, *v*/*v*), Cosmosil 5C_18_-MS-II column) to provide eight fractions (Fr. 6-12-1–Fr. 6-12-8). Fraction 6-12-4 (143.4 mg) was purified by PHPLC (CH_3_CN–1% CH_3_COOH (48:52, *v*/*v*), Cosmosil 5C_18_-MS-II column) to gain Yucca spirostanoside C_2_ (**7**, 49.4 mg, *t*_R_ 23.06′). Fraction 6-12-5 (165.4 mg) was separated by PHPLC (CH_3_CN–1% CH_3_COOH (45:55, *v*/*v*), Cosmosil 5C_18_-MS-II column) to yield Yucca spirostanoside C_1_ (**6**, 32.2 mg, *t*_R_ 21.02′). Fraction 6-13 (1.2 g) was subjected to silica gel CC (CH_2_Cl_2_–MeOH (100:3 → 100:5 → 100:7) → MeOH, *v*/*v*) to produce nine fractions (Fr. 6-13-1–Fr. 6-13-9). Fraction 6-13-3 (446.3 mg) was isolated by PHPLC [MeOH–1% CH_3_COOH (90:10, *v*/*v*), Cosmosil 5C_18_-MS-II column] to provide Yucca spirostanoside A_1_ (**1**, 42.1 mg, *t*_R_ 33.03′). Fraction 6-13-5 (740.6 mg) was further purified by PHPLC (MeOH–1% CH_3_COOH (85:15, *v*/*v*), Cosmosil 5C_18_-MS-II column) to give Yucca spirostanoside A_2_ (**2**, 56.6 mg, *t*_R_ 31.04′). Fraction 7 (10.0 g) was separated by PHPLC (MeOH–1% CH_3_COOH (80:20, *v*/*v*), Cosmosil 5C_18_-MS-II column), and 13 fractions (Fr. 7-1–Fr. 7-13) were obtained. Fraction 7-11 (984.6 mg) was isolated by PHPLC (MeOH–1% CH_3_COOH (95:5, *v*/*v*), Cosmosil PBr column) to gain four fractions (Fr. 7-11-1–Fr. 7-11-4). Fraction 7-11-4 (60.8 mg) was purified by PHPLC (CH_3_CN–1% CH_3_COOH (55:45, *v*/*v*), Wacopak Navi C_30_-5 column) to provide schidigera-saponin A1 (**11**, 10.3 mg, *t*_R_ 42.24′). Fraction 8 (10.0 g) was separated by PHPLC (MeOH–1% CH_3_COOH (80:20, *v*/*v*), Cosmosil 5C_18_-MS-II column], and 16 fractions (Fr. 8-1–Fr. 8-16) were given. Fraction 8-15 (80.4 mg) was purified by PHPLC (MeOH–1% CH_3_COOH (75:25, *v*/*v*), Cosmosil 5C_18_-MS-II column) to yield schidigera-saponin C2 (**13**, 40.2 mg, *t*_R_ 26.17′). Fraction 9 (12.4 g) was isolated by PHPLC (MeOH–H_2_O (80:20, *v*/*v*) + 1% CH_3_COOH, Cosmosil 5C_18_-MS-II column) to afford 16 fractions (Fr. 9-1–Fr. 9-16). Fraction 9-5 (120.1 mg) was purified by PHPLC (CH_3_CN–1% CH_3_COOH (32:68, *v*/*v*), Cosmosil PBr column) to yield Yucca spirostanosides B_3_ (**5**, 10.0 mg, *t*_R_ 65.87′) and D_1_ (**9**, 33.5 mg, *t*_R_ 64.75′). Fraction 9-7 (220.2 mg) was separated by PHPLC (MeOH–1% CH_3_COOH (85:15, *v*/*v*), Cosmosil 5C_18_-MS-II column] to gain Yucca spirostanoside C_3_ (**8**, 11.7 mg, *t*_R_ 42.85′). Fraction 9-8 (549.4 mg) was purified by PHPLC (CH_3_CN–1% CH_3_COOH (38:62, *v*/*v*), Cosmosil 5C_18_-MS-II column] to produce 5β-spirost-25(27)-en-3β-ol-12-one 3-*O*-{β-d-glucopyranosyl-(1→2)-*O*-[β-d-glucopyranosyl-(1→3)]-β-d-glucopyranoside} (**12**, 253.8 mg, *t*_R_ 46.73′). Fraction 9-15 (151.9 mg) was subjected to PHPLC (CH_3_CN–1% CH_3_COOH (45:55, *v*/*v*), Cosmosil 5C_18_-MS-II column] to obtain schidigera-saponin C1 (**14**, 97.8 mg, *t*_R_ 28.06′). Fraction 9-16 (400.0 mg) was separated by PHPLC (MeOH–1% CH_3_COOH (85:15, *v*/*v*), Cosmosil 5C_18_-MS-II column) to provide six fractions (Fr. 9-16-1–Fr. 9-16-6). Fraction 9-16-5 (189.1 mg) was further purified by PHPLC [MeOH–1% CH_3_COOH (80:20, *v*/*v*), Wacopak Navi C_30_-5 column] to afford schidigera-saponin A3 (**10**, 68.0 mg, *t*_R_ 23.04′).

*Yucca spirostanoside A*_1_ (**1**): White powder; ${\left[\alpha \right]}_{D}^{25}$ −45.4° (*c* = 0.41, MeOH); IR *ν*_max_ (KBr) cm^−1^: 3370, 2928, 1651, 1451, 1375, 1231, 1169, 1077, 1043, 1019, 921; ^1^H NMR (C_5_D_5_N, 500 MHz) data: δ 1.47, 1.72 (1H each, both m, H_2_-1), [1.54 (1H, m, overlapped), 1.90 (1H, m), H_2_-2], 4.37 (1H, m, H-3), [1.75 (1H, m, overlapped), 1.82 (1H, m), H_2_-4], 2.02 (1H, m, H-5), [1.09 (1H, m), 1.76 (1H, m, overlapped), H_2_-6], [0.99 (1H, m), 1.30 (1H, m, overlapped), H_2_-7], 1.51 (1H, m, H-8), 1.31 (1H, m, overlapped, H-9), 1.21, 1.33 (1H each, both m, H_2_-11), [1.10 (1H, m, overlapped), 1.69 (1H, m), H_2_-12], 1.11 (1H, m, overlapped, H-14), 1.42, 2.04 (1H each, both m, H_2_-15), 4.61 (1H, q like, ca. *J* = 8 Hz, H-16), 1.86 (1H, dd, *J* = 6.5, 8.5 Hz, H-17), 0.84 (3H, s, H_3_-18), 0.85 (3H, s, H_3_-19), 1.98 (1H, m, H-20), 1.11 (3H, d, *J* = 7.0 Hz, H_3_-21), 1.79 (2H, m, H_2_-23), [2.25 (1H, m), 2.72 (1H, dt, *J* = 5.0, 13.0 Hz), H_2_-24], [4.04 (1H, d, *J* = 12.5 Hz), 4.48 (1H, d, *J* = 12.5 Hz), H_2_-26], 4.79, 4.82 (1H each, both br. s, H_2_-27), 4.93 (1H, d, *J* = 8.0 Hz, H-1′), 4.03 (1H, dd, *J* = 8.0, 9.0 Hz, H-2′), 4.25 (2H, m, H-3′, 4′), 3.94 (1H, m, H-5′), [4.40 (1H, dd, *J* = 4.5, 12.0 Hz), 4.53 (1H, dd, *J* = 2.5, 12.0 Hz), H_2_-6′]; ^13^C NMR (C_5_D_5_N, 125 MHz) data, see Table 1; HRESI-TOF-MS Positive-ion mode *m/z* - 577.3758 [M + H]^+^ (calcd for C_33_H_53_O_8_, 577.3735).

*Yucca spirostanoside A*_1_ (**2**): White powder; ${\left[\alpha \right]}_{D}^{25}$ –53.5° (*c* = 0.40, MeOH); IR *ν*_max_ (KBr) cm^−1^: 3373, 2928, 1651, 1451, 1374, 1233, 1161, 1080, 1042, 922; ^1^H NMR (C_5_D_5_N, 500 MHz) data: δ [1.47 (1H, m), 1.71 (1H, m, overlapped), H_2_-1], [1.54 (1H, m, overlapped), 1.91 (1H, m), H_2_-2], 4.34 (1H, m, H-3), [1.75 (1H, m, overlapped), 1.83 (1H, m), H_2_-4], 2.02 (1H, m, H-5), 1.14, 1.80 (1H each, both m, H_2_-6), [1.01 (1H, m), 1.31 (1H, m, overlapped), H_2_-7], 1.53 (1H, m, overlapped, H-8), 1.32 (1H, m, overlapped, H-9), [1.23 (1H, m), 1.33 (1H, m, overlapped), H_2_-11], 1.10,1.70 (1H each, both m, overlapped, H_2_-12), 1.11 (1H, m, overlapped, H-14), 1.42, 2.05 (1H each, both m, H_2_-15), 4.62 (1H, q like, ca. *J* = 8 Hz, H-16), 1.86 (1H, dd, *J* = 6.5, 8.5 Hz, H-17), 0.84 (3H, s, H_3_-18), 0.87 (3H, s, H_3_-19), 1.98 (1H, m, H-20), 1.10 (3H, d, *J* = 6.5 Hz, H_3_-21), 1.78 (2H, m, H_2_-23), [2.26 (1H, m), 2.72 (1H, dt, *J* = 5.0, 12.5 Hz), H_2_-24], [4.04 (1H, d, *J* = 11.0 Hz), 4.48 (1H, d, *J* = 11.0 Hz), H_2_-26], 4.79, 4.82 (1H each, both br. s, H_2_-27), 4.91 (1H, d, *J* = 7.5 Hz, H-1′), 4.05 (1H, dd, *J* = 7.5, 8.0 Hz, H-2′), 4.22 (1H, dd, *J* = 8.0, 9.0 Hz, H-3′), 4.15 (1H, dd, *J* = 9.0, 9.0 Hz, H-4′), 3.89 (1H, m, H-5′), [4.32 (1H, dd, *J* = 5.0, 12.0 Hz), 4.47 (1H, dd, *J* = 2.5, 12.0 Hz), H_2_-6′], 5.26 (1H, d, *J* = 8.0 Hz, H-1′′), 4.00 (1H, dd, *J* = 8.0, 8.5 Hz, H-2′′), 4.11 (1H, dd, *J* = 8.5, 8.5 Hz, H-3′′), 4.13 (1H, m, H-4′′), [3.67 (1H, dd, *J* = 11.0, 11.0 Hz), 4.29 (1H, dd, *J* = 4.5, 11.0 Hz), H_2_-5′′]; ^13^C NMR (C_5_D_5_N, 125 MHz) data: see Table 1; HRESI-TOF-MS Positive-ion mode *m/z* 709.4178 [M + H]^+^ (calcd for C_38_H_61_O_12_, 709.4158).

*Yucca spirostanoside B*_1_ (**3**): White powder; ${\left[\alpha \right]}_{D}^{25}$ −45.2° (*c* = 0.42, MeOH); IR *ν*_max_ (KBr) cm^−1^: 3385, 2928, 2857, 1649, 1451, 1372, 1234, 1158, 1078, 1040, 919; ^1^H NMR (C_5_D_5_N, 500 MHz) δ [1.46 (1H, m), 1.77 (1H, m, overlapped), H_2_-1], 1.53, 1.90 (1H each, both m, H_2_-2), 4.35 (1H, m, H-3), [1.75 (1H, m, overlapped), 1.81 (1H, m), H_2_-4], 2.04 (1H, m, H-5), [1.12 (1H, m), 1.76 (1H, m, overlapped), H_2_-6], 0.98, 1.34 (1H each, both m, H_2_-7), 1.55 (1H, m, H-8), 1.49 (1H, m, overlapped, H-9), 1.50, 1.78 (1H each, both m, overlapped, H_2_-11), 3.54 (1H, dd, *J* = 5.5, 10.0 Hz, H-12), 1.16 (1H, m, H-14), 1.61, 2.11 (1H each, both m, H_2_-15), 4.70 (1H, q like, ca. *J* = 8 Hz, H-16), 2.23 (1H, m, overlapped, H-17), 1.09 (3H, s, H_3_-18), 0.87 (3H, s, H_3_-19), 2.23 (1H, m, overlapped, H-20), 1.39 (3H, d, *J* = 6.5 Hz, H_3_-21), 1.84 (2H, m, H_2_-23), [2.26 (1H, m), 2.75 (1H, dt, *J* = 6.0, 13.0 Hz), H_2_-24], [4.06 (1H, d, *J* = 12.0 Hz), 4.54 (1H, d, *J* = 12.0 Hz), H_2_-26], 4.79, 4.82 (1H each, both br. s, H_2_-27), 4.94 (1H, d, *J* = 8.0 Hz, H-1′), 4.04 (1H, dd, *J* = 8.0, 9.0 Hz, H-2′), 4.25 (2H, m, H-3′, 4′), 3.96 (1H, m, H-5′), [4.39 (1H, dd, *J* = 5.5, 12.0 Hz), 4.55 (1H, dd, *J* = 2.5, 12.0 Hz), H_2_-6′]; ^13^C NMR (C_5_D_5_N, 125 MHz) data, see Table 1; HRESI-TOF-MS Positive-ion mode *m/z* 593.3700 [M + H]^+^ (calcd for C_33_H_53_O_9_, 593.3684).

*Yucca spirostanoside B*_2_ (**4**): White powder; ${\left[\alpha \right]}_{D}^{25}$ –36.2° (*c* = 0.37, MeOH); IR *ν*_max_ (KBr) cm^−1^: 3389, 2928, 2872, 1650, 1452, 1373, 1264, 1161, 1079, 1041, 921; ^1^H NMR (C_5_D_5_N, 500 MHz) δ [1.48 (1H, m), 1.77 (1H, m, overlapped), H_2_-1], 1.51, 1.86 (1H each, both m, H_2_-2), 4.32 (1H, m, H-3), 1.75, 1.81 (1H each, both m, overlapped, H_2_-4), 2.04 (1H, m, H-5), 1.16, 1.80 (1H each, both m, overlapped, H_2_-6), 1.00, 1.34 (1H each, both m, H_2_-7), 1.56 (1H, m, H-8), 1.49 (1H, m, overlapped, H-9), 1.50, 1.79 (1H each, both m, overlapped, H_2_-11), 3.55 (1H, dd, *J* = 5.0, 10.0 Hz, H-12), 1.16 (1H, m, overlapped, H-14), 1.61, 2.11 (1H each, both m, H_2_-15), 4.70 (1H, q like, ca. *J* = 8 Hz, H-16), 2.23 (1H, m, overlapped, H-17), 1.09 (3H, s, H_3_-18), 0.89 (3H, s, H_3_-19), 2.23 (1H, m, overlapped, H-20), 1.40 (3H, d, *J* = 6.0 Hz, H_3_-21), 1.84 (2H, m, H_2_-23), [2.26 (1H, m), 2.76 (1H, dt, *J* = 5.5, 13.0 Hz), H_2_-24], [4.06 (1H, m, overlapped), 4.53 (1H, d, *J* = 12.0 Hz), H_2_-26], 4.79, 4.82 (1H each, both br. s, H_2_-27), 4.91 (1H, d, *J* = 8.0 Hz, H-1′), 4.06 (1H, m, overlapped, H-2′), 4.24 (1H, dd, *J* = 9.0, 9.0 Hz, H-3′), 4.18 (1H, dd, *J* = 9.0, 9.0 Hz, H-4′), 3.89 (1H, m, H-5′), [4.30 (1H, dd, *J* = 5.0, 12.0 Hz), 4.48 (1H, br. d, ca. *J* = 12 Hz), H_2_-6′], 5.28 (1H, d, *J* = 7.5 Hz, H-1′′), 4.02 (1H, dd, *J* = 7.5, 8.0 Hz, H-2′′), 4.13 (1H, dd, *J* = 8.0, 9.0 Hz, H-3′′), 4.15 (1H, m, H-4′′), [3.69 (1H, dd, *J* = 11.5, 11.5 Hz), 4.29 (1H, dd, *J* = 4.5, 11.5 Hz), H_2_-5′′]; ^13^C NMR (C_5_D_5_N, 125 MHz) data, see Table 1; HRESI-TOF-MS Positive-ion mode *m/z* 725.4121 [M + H]^+^ (calcd for C_38_H_61_O_13_, 725.4107).

*Yucca spirostanoside B*_3_ (**5**): White powder; ${\left[\alpha \right]}_{D}^{25}$ –33.0° (*c* = 0.23, MeOH); IR *ν*_max_ (KBr) cm^−1^: 3364, 2928, 2861, 1648, 1452, 1369, 1264, 1159, 1078, 1039, 920; ^1^H NMR (C_5_D_5_N, 500 MHz) δ 1.46, 1.84 (1H each, both m, overlapped, H_2_-1), [1.47 (1H, m, overlapped), 1.88 (1H, m), H_2_-2], 4.30 (1H, m, overlapped, H-3), 1.84 (2H, m, H_2_-4), 2.19 (1H, m, H-5), [1.20 (1H, m), 1.82 (1H, m, overlapped), H_2_-6], 0.96, 1.31 (1H each, both m, H_2_-7), 1.55 (1H, m, H-8), 1.47 (1H, m, overelapped, H-9), 1.50, 1.77 (1H each, both m, H_2_-11), 3.55 (1H, dd, *J* = 5.5, 10.0 Hz, H-12), 1.13 (1H, m, H-14), 1.60, 2.11 (1H each, both m, H_2_-15), 4.71 (1H, q like, ca. *J* = 8 Hz, H-16), 2.23 (1H, m, overlapped, H_3_-17), 1.09 (3H, s, H_3_-18), 0.97 (3H, s, H_3_-19), 2.23 (1H, m, overlapped, H-20), 1.40 (3H, d, *J* = 6.0 Hz, H_3_-21), 1.84 (2H, m, overlapped, H_2_-23), [2.27 (1H, m), 2.76 (1H, dt, *J* = 6.0, 13.0 Hz), H_2_-24], [4.08 (1H, d, *J* = 11.5 Hz), 4.53 (1H, d, *J* = 11.5 Hz), H_2_-26], 4.79, 4.83 (1H each, both br. s, H_2_-27), 4.87 (1H, d, *J* = 7.5 Hz, H-1′), 4.37 (1H, dd, *J* = 7.5, 9.0 Hz, H-2′), 4.27 (1H, m, overlapped, H-3′), 4.04 (1H, m, overlapped, H-4′), 3.79 (1H, m, H-5′), [4.23 (1H, m, overlapped), 4.41 (1H, m, overlapped), H_2_-6′], 5.65 (1H, d, *J* = 7.5 Hz, H-1′′), 4.07 (1H, m, overlapped, H-2′′), 4.28 (1H, m, overlapped, H-3′′), 4.20 (1H, dd, *J* = 9.0, 9.0 Hz, H-4′′), 3.97 (1H, m, H-5′′), [4.43 (1H, m, overlapped), 4.56 (1H, m, overlapped), H_2_-6′′], 5.35 (1H, d, *J* = 7.5 Hz, H-1′′′), 4.06 (1H, m, overlapped, H-2′′′), 4.22 (1H, dd, *J* = 8.0, 9.0 Hz, H-3′′′), 4.15 (1H, dd, *J* = 9.0, 9.0 Hz, H-4′′′), 4.04 (1H, m, H-5′′′), [4.28 (1H, m, overlapped), 4.54 (1H, m, overlapped), H_2_-6′′′]; ^13^C NMR (C_5_D_5_N, 125 MHz) data, see Table 1; HRESI-TOF-MS Positive-ion mode *m/z* 939.4565 [M + Na]^+^ (calcd for C_45_H_72_O_19_Na, 939.4560).

*Yucca spirostanoside C*_1_ (**6**): White powder; [α${]}_{D}^{25}$ –8.9° (*c* = 0.09, MeOH); IR *ν*_max_ (KBr) cm^−1^: 3388, 2928, 2872, 1703, 1650, 1452, 1375, 1266, 1164, 1078, 1042, 922; ^1^H NMR (C_5_D_5_N, 500 MHz) δ [1.28 (1H, m), 1.75 (1H, m, overlapped), H_2_-1], [1.42 (1H, m), 1.85 (1H, m, overlapped), H_2_-2], 4.33 (1H, m, H-3), 1.74 (2H, m, overlapped, H_2_-4), 2.09 (1H, m, H-5), [1.12 (1H, m), 1.76 (1H, m, overlapped), H_2_-6], 0.98, 1.34 (1H each, both m, H_2_-7), 1.84 (1H, m, overlapped, H-8), 1.76 (1H, m, overlapped, H-9), [2.21 (1H, dd, *J* = 4.5, 14.0 Hz), 2.38 (1H, dd, *J* = 14.0, 14.0 Hz), H_2_-11], 1.49 (1H, m, H-14), [1.63 (1H, m), 2.15 (1H, m), H_2_-15], 4.56 (1H, m, overlapped, H-16), 2.83 (1H, dd, *J* = 6.5, 8.5 Hz, H-17), 1.10 (3H, s, H_3_-18), 0.86 (3H, s, H_3_-19), 1.96 (1H, m, H-20), 1.34 (3H, d, *J* = 6.5 Hz, H_3_-21), 1.77 (2H, m, overlapped, H_2_-23), [2.26 (1H, m), 2.72 (1H, dt, *J* = 6.0, 13.0 Hz), H_2_-24], [4.06 (1H, d, *J* = 12.5 Hz), 4.47 (1H, d, *J* = 12.5 Hz), H_2_-26], 4.80, 4.84 (1H each, both br. s, H_2_-27), 4.94 (1H, d, *J* = 8.0 Hz, H-1′), 4.07 (1H, m, overlapped, H-2′), 4.28 (2H, m, H-3′, 4′), 3.96 (1H, m, H-5′), [4.41 (1H, dd, *J* = 5.5, 12.0 Hz), 4.56 (1H, m, overlapped), H_2_-6′]; ^13^C NMR (C_5_D_5_N, 125 MHz) data, see Table 1; HRESI-TOF-MS Positive-ion mode *m/z* 591.3557 [M + H]^+^ (calcd for C_33_H_51_O_9_, 591.3528).

*Yucca spirostanoside C*_2_ (**7**)*:* White powder; ${\left[\alpha \right]}_{D}^{25}$ –8.5° (*c* = 0.77, MeOH); IR *ν*_max_ (KBr) cm^−1^: 3371, 2928, 1705, 1651, 1452, 1375, 1265, 1163, 1082, 1041, 922; ^1^H NMR (C_5_D_5_N, 500 MHz) δ [1.28 (1H, m), 1.70 (1H, m, overlapped), H_2_-1], [1.42 (1H, m), 1.85 (1H, m, overlapped), H_2_-2], 4.29 (1H, m, overlapped, H-3), 1.74 (2H, m, overlapped, H_2_-4), 2.08 (1H, m, H-5), [1.15 (1H, m), 1.80 (1H, m, overlapped), H_2_-6], 1.00, 1.36 (1H each, both m, H_2_-7), 1.85 (1H, m, overlapped, H-8), 1.76 (1H, m, overlapped, H-9), [2.20 (1H, dd, *J* = 5.0, 13.5 Hz), 2.38 (1H, dd, *J* = 13.5, 13.5 Hz), H_2_-11], 1.49 (1H, m, H-14), 1.63, 2.15 (1H each, both m, H_2_-15), 4.55 (1H, q like, ca. *J* = 9 Hz H-16), 2.82 (1H, dd, *J* = 7.0, 9.0 Hz, H-17), 1.10 (3H, s, H_3_-18), 0.87 (3H, s, H_3_-19), 1.95 (1H, m, H-20), 1.33 (3H, d, *J* = 7.0 Hz, H_3_-21), [1.77 (1H, m, overlapped), 1.82 (1H, m, overlapped), H_2_-23], [2.26 (1H, m), 2.72 (1H, dt, *J* = 5.0, 13.0 Hz), H_2_-24], [4.06 (1H, d, *J* = 12.5 Hz), 4.46 (1H, d, *J* = 12.5 Hz), H_2_-26], 4.80, 4.84 (1H each, both br. s, H_2_-27), 4.90 (1H, d, *J* = 8.0 Hz, H-1′), 4.06 (1H, dd, *J* = 8.0, 9.0 Hz, H-2′), 4.24 (1H, dd, *J* = 9.0, 9.0 Hz, H-3′), 4.17 (1H, dd, *J* = 9.0, 9.0 Hz, H-4′), 3.89 (1H, m, H-5′), [4.33 (1H, dd, *J* = 5.0, 12.0 Hz), 4.49 (1H, dd, *J* = 2.0, 12.0 Hz), H_2_-6′], 5.28 (1H, d, *J* = 7.0 Hz, H-1′′), 4.02 (1H, dd, *J* = 7.0, 8.5 Hz, H-2′′), 4.14 (1H, dd, *J* = 8.5, 8.5 Hz, H-3′′), 4.15 (1H, m, H-4′′), [3.67 (1H, dd, *J* = 11.0, 11.0 Hz), 4.31 (1H, m, overlapped), H_2_-5′′]; ^13^C NMR (C_5_D_5_N, 125 MHz) data, see Table 1; HRESI-TOF-MS Positive-ion mode *m/z* 723.3959 [M + H]^+^ (calcd for C_38_H_59_O_13_, 723.3950).

*Yucca spirostanoside C*_3_ (**8**): White powder; ${\left[\alpha \right]}_{D}^{25}$ –5.0° (*c* = 0.16, MeOH); IR *ν*_max_ (KBr) cm^−1^: 3398, 2929, 2879, 1701, 1650, 1451, 1375, 1223, 1166, 1158, 1077, 1036, 921; ^1^H NMR (C_5_D_5_N, 500 MHz) δ [1.24 (1H, m), 1.78 (1H, m, overlapped), H_2_-1], [1.26 (1H, m), 1.81 (1H, m, overlapped), H_2_-2], 4.24 (1H, m, overlapped, H-3), 1.78 (2H, m, overlapped, H_2_-4), 2.28 (1H, m, H-5), [1.21 (1H, m), 1.81 (1H, m, overlapped), H_2_-6], [0.95 (1H, m), 1.33 (1H, m, overlapped), H_2_-7], 1.82 (1H, m, overlapped, H-8), 1.73 (1H, m, H-9), [2.18 (1H, dd, *J* = 4.5, 14.0 Hz), 2.36 (1H, dd, *J* = 14.0, 14.0 Hz), H_2_-11], 1.46 (1H, m, H-14), [1.62 (1H, m), 2.12 (1H, m), H_2_-15], 4.57 (1H, q like, ca. *J* = 7 Hz, H-16), 2.83 (1H, dd, *J* = 6.5, 8.5 Hz, H-17), 1.10 (3H, s, H_3_-18), 0.98 (3H, s, H_3_-19), 1.96 (1H, m, H-20), 1.33 (3H, d, *J* = 7.0 Hz, H_3_-21), 1.82 (2H, m, overlapped, H_2_-23), [2.27 (1H, m), 2.73 (1H, dt, *J* = 6.0, 13.0 Hz), H_2_-24], [4.06 (1H, d, *J* = 12.5 Hz), 4.47 (1H, d, *J* = 12.5 Hz), H_2_-26], 4.80, 4.83 (1H each, both br. s, H_2_-27), 4.83 (1H, m, overlapped, H-1′), 4.80 (1H, m, overlapped, H-2′), 4.36 (1H, m, overlapped, H-3′), 4.80 (1H, m, overlapped, H-4′), 3.98 (1H, m, H-5′), 4.35 (2H, m, overlapped, H_2_-6′), 5.57 (1H, d, *J* = 7.5 Hz, H-1′′), 4.03 (1H, dd, *J* = 7.5, 8.5 Hz, H-2′′), 4.23 (1H, m, overlapped, H-3′′), 4.16 (1H, dd, *J* = 9.0, 9.0 Hz, H-4′′), 3.81 (1H, m, H-5′′), [4.35 (1H, m, overlapped), 4.50 (1H, dd, *J* = 3.0, 11.5 Hz), H_2_-6′′], 5.37 (1H, d, *J* = 7.5 Hz, H-1′′′), 4.00 (1H, dd, *J* = 7.5, 8.0 Hz, H-2′′′), 4.23 (1H, m, overlapped, H-3′′′), 4.22 (1H, m, overlapped, H-4′′′), 3.92 (1H, m, H-5′′′), [4.29 (1H, dd, *J* = 5.5, 11.5 Hz), 4.43 (1H, dd, *J* = 2.0, 11.5 Hz), H_2_-6′′′]; ^13^C NMR (C_5_D_5_N, 125 MHz) data, see Table 1; HRESI-TOF-MS Positive-ion mode *m/z* 937.4412 [M + Na]^+^ (calcd for C_45_H_70_O_19_Na, 937.4404).

*Yucca spirostanoside D*_1_ (**9**)*:* White powder; ${\left[\alpha \right]}_{D}^{25}$ –26.2° (*c* = 0.45, MeOH); IR *ν*_max_ (KBr) cm^−1^: 3461, 3389, 2930, 2877, 1696, 1654, 1450, 1376, 1266, 1231, 1159, 1071, 1042, 916; ^1^H NMR (C_5_D_5_N, 500 MHz) δ 1.72, 1.96 (1H each, both m, overlapped, H_2_-1), 3.75 (1H, m, H-2), 4.41 (1H, m, overlapped, H-3), 1.82, 1.97 (1H each, both m, overlapped, H_2_-4), 2.26 (1H, m, overlapped, H-5), [1.32 (1H, m, overlapped), 1.78 (1H, m), H_2_-6], [0.94 (1H, m), 1.32 (1H, m, overlapped), H_2_-7], 1.81 (1H, m, overlapped, H-8), 1.72 (1H, m, overlapped, H-9), [2.37 (1H, dd, *J* = 5.0, 14.0 Hz), 2.41 (1H, dd, *J* = 14.0, 14.0 Hz), H_2_-11], 1.46 (1H, m, H-14), 1.62, 2.13 (1H each, both m, H_2_-15), 4.56 (1H, q like, ca. *J* = 8 Hz, H-16), 2.82 (1H, dd, *J* = 6.5, 8.5 Hz, H-17), 1.09 (3H, s, H_3_-18), 0.99 (3H, s, H_3_-19), 1.95 (1H, m, overlapped, H-20), 1.31 (3H, d, *J* = 6.5 Hz, H_3_-21), 1.79 (2H, m, overlapped, H_2_-23), [2.27 (1H, m, overlapped), 2.73 (1H, dt, *J* = 6.0, 13.0 Hz), H_2_-24], 4.06, 4.47 (1H each, both m, overlapped, H_2_-26), 4.80, 4.84 (1H each, both br. s, H_2_-27), 4.98 (1H, d, *J* = 7.5 Hz, H-1′), 4.87 (1H, dd, *J* = 7.5, 9.5 Hz, H-2′), 4.31 (1H, dd, *J* = 2.5, 9.5 Hz, H-3′), 4.75 (1H, m, overlapped, H-4′), 4.11 (1H, m, overlapped, H-5′), 4.39 (2H, m, H_2_-6′), 5.57 (1H, d, *J* = 8.0 Hz, H-1′′), 4.03 (1H, dd, *J* = 8.0, 8.0 Hz, H-2′′), 4.23 (1H, dd, *J* = 8.0, 9.0 Hz, H-3′′), 4.09 (1H, m, overlapped, H-4′′), 3.77 (1H, m, H-5′′), [4.28 (1H, dd, *J* = 4.5, 11.5 Hz), 4.48 (1H, m, overlapped), H_2_-6′′], 5.22 (1H, d, *J* = 7.5 Hz, H-1′′′), 3.95 (1H, dd, *J* = 7.5, 9.5 Hz, H-2′′′), 4.12 (1H, m, overlapped, H-3′′′), 4.13 (1H, m, overlapped, H-4′′′), [3.60 (1H, dd, *J* = 10.0, 10.0 Hz), 4.19 (1H, dd, *J* = 4.5, 10.0 Hz), H_2_-5′′′]; ^13^C NMR (C_5_D_5_N, 125 MHz) data, see Table 1; HRESI-TOF-MS Positive-ion mode *m/z* 923.4293 [M + Na]^+^ (calcd for C_44_H_68_O_19_Na, 923.4247).

*Acid Hydrolysis of* **1**–**9**: A solution of each saponin (about 3.0 mg) in 1 M HCl (1 mL) was heated under reflux for 3 h, respectively. Then each reaction mixture was neutralized with Amberlite IRA-400 (OH^−^ form) and removed by filtration. The aqueous layer was subjected to HPLC analysis: HPLC column, Kaseisorb LC NH_2_-60-5, 4.6 mm i.d. × 250 mm (Tokyo Kasei Co., Ltd., Tokyo, Japan); detection, optical rotation (Shodex OR-2 (Showa Denko Co., Ltd., Tokyo, Japan)]; mobile phase, CH_3_CN-H_2_O (75:25, *v*/*v*; flow rate 1.0 mL/min). As a result, d-xylose (6.0 min, positive optical rotation) for **2**, **4**, **7**, **9**; d-galactose (7.2 min, positive optical rotation) for **8**, **9**; and d-glucose (12.6 min, positive optical rotation) for **1**–**9** were identified by comparison of their retention times and optical rotations with those of authentic samples.

## 4. Conclusions

In summary, during the investigation of spirostanol saponins from natural products, fourteen spirostane-type saponins, including nine new ones, Yucca spirostanosides A_1_ (**1**), A_2_ (**2**), B_1_ (**3**), B_2_ (**4**), B_3_ (**5**), C_1_ (**6**), C_2_ (**7**), C_3_ (**8**), and D_1_ (**9**), along with five known ones (**10**–**14**) were obtained from the stems of *Y. schidigera*. Their structures were determined by means of chemical and spectroscopic methods. 

In accordance to the increasing applications of yucca extracts, further analytical, biological and physicochemical studies are still required. The presented study will make people understand the phytochemical constituents of *Y. schidigera* more fully and will lay a solid foundation for further pharmacodynamics research.

## Figures and Tables

**Table 1:** ^13^C NMR data for **1**–**9** in C_5_D_5_N.

NO.	1	2	3	4	5	6	7	8	9
1	31.0	30.9	31.0	31.0	31.0	30.6	30.6	30.5	39.6
2	27.0	27.0	26.9	26.9	26.8	26.7	26.6	26.7	66.7
3	74.3	74.4	74.3	74.4	75.7	73.9	74.0	75.0	79.7
4	30.5	30.4	30.5	30.4	31.0	30.2	30.1	30.7	30.5
5	37.0	37.0	36.8	36.8	36.7	36.5	36.5	36.2	35.3
6	27.0	27.0	27.1	27.1	27.2	26.8	26.8	27.0	26.0
7	26.8	26.8	26.7	26.7	26.8	26.4	26.4	26.6	26.3
8	35.6	35.6	34.7	34.7	34.8	34.7	34.7	34.9	34.6
9	40.3	40.3	39.4	39.4	39.6	41.9	41.9	42.2	42.8
10	35.2	35.3	35.3	35.3	35.4	35.7	35.7	35.9	37.3
11	21.2	21.2	31.4	31.5	31.6	37.7	37.7	37.9	37.8
12	40.3	40.3	79.4	79.4	79.5	213.0	213.0	213.2	212.6
13	40.9	41.0	46.7	46.7	46.9	55.6	55.6	55.8	55.4
14	56.5	56.5	55.3	55.3	55.4	56.0	56.0	56.2	55.6
15	32.1	32.1	31.9	31.9	32.0	31.4	31.4	31.6	31.3
16	81.6	81.6	81.7	81.7	81.8	80.1	80.1	80.3	80.0
17	63.2	63.2	63.0	63.0	63.2	54.3	54.3	54.5	54.2
18	16.6	16.6	11.2	11.2	11.3	16.1	16.1	16.2	15.8
19	23.9	23.9	23.8	23.8	24.0	23.0	23.1	23.3	22.8
20	41.9	41.9	42.9	42.9	43.1	42.5	42.5	42.7	42.4
21	15.0	15.0	14.3	14.3	14.5	13.9	13.9	14.0	13.7
22	109.4	109.4	109.7	109.7	109.9	109.5	109.5	109.7	109.3
23	33.3	33.3	33.4	33.4	33.5	33.2	33.2	33.4	33.1
24	29.0	29.0	29.0	29.1	29.2	28.9	28.9	29.1	28.7
25	144.4	144.4	144.6	144.6	144.7	144.2	144.2	144.4	144.1
26	65.0	65.0	65.1	65.1	65.2	65.1	65.1	65.3	64.9
27	108.7	108.7	108.6	108.6	108.8	108.9	108.9	109.0	108.7
1′	103.1	102.5	103.1	102.6	102.1	102.9	102.3	102.0	101.6
2′	75.3	74.2	75.4	74.3	80.1	75.4	74.2	77.8	77.1
3′	78.7	87.8	78.8	87.8	88.4	78.7	87.7	84.3	84.1
4′	71.8	69.6	71.8	69.6	70.1	71.7	69.5	70.0	69.7
5′	78.4	78.1	78.4	78.1	78.0	78.4	78.1	76.6	76.6
6′	62.9	62.5	62.9	62.5	62.6	62.8	62.3	62.5	61.8
1′′		106.3		106.4	104.4		106.3	104.6	104.2
2′′		75.3		75.4	76.6		75.3	76.5	76.2
3′′		78.1		78.2	78.4		78.1	78.5	78.5
4′′		70.9		70.9	72.6		70.9	72.9	72.6
5′′		67.4		67.4	78.4		67.4	78.1	77.8
6′′					63.5			63.6	63.3
1′′′					105.0			105.5	106.0
2′′′					75.5			75.5	75.0
3′′′					78.7			78.7	78.3
4′′′					71.8			71.7	70.9
5′′′					78.8			78.5	67.0
6′′′					62.6			62.7	

## Supplementary Materials

Supplementary data (NMR and MS spectroscopic data for all new compounds) associated with this article can be found in the online version.
