# Supplementary material for: Spirostane-Type Saponins Obtained from Yucca schidigera
Source: Molecules. 2018 Jan 14;23(1):167. doi: 10.3390/molecules23010167 (PMC6016961; doi:10.3390/molecules23010167)
Supplement: Supplementary file 1 [file molecules-23-00167-s001.pdf]

Supporting Information

Spirostane-type saponins obtained from *Yucca schidigera*

Lu Qu [1], Jianli Wang [2], JingYa Ruan [1], Xiaoyong Yao [3], Peijian Huang [2], Yue Wang [2], Haiyang Yu [2], Lifeng Han [2], Yi Zhang [1,2,*], and Tao Wang [1,2,*]

[1] Tianjin State Key Laboratory of Modern Chinese Medicine, 312 Anshanxi Road, Nankai District,Tianjin 300193, China; qululuhan88@163.com (L.Q.); Ruanjy19930919@163.com (J.R.)

[2] Tianjin Key Laboratory of TCM Chemistry and Analysis, Institute of Traditional Chinese Medicine, Tianjin University of Traditional Chinese Medicine, 312 Anshanxi Road, Nankai District, Tianjin 300193, China; wjl15802226160@126.com (J. W.); hpjforever@sina.com (P.H.); wy1609112949@163.com (Y. W.); yuhaiyang19830116@hotmail.com (H.Y.); hanlifeng_1@sohu.com (L.H.)

[3] Risun Bio-Tech Inc., D/17F, Haibo Business Building, FengCheng 9th Road, Xi'an, 710018, China; denny@risunextract.com (X.Y.)

* Correspondence: zhwwxzh@263.net (Y.Z.); wangtao@tjutcm.edu.cn (T.W.); Tel./Fax: +86-22-5959-6163 (Y.Z.); +86-22-59596168 (T.W.)

S1. HRESI-TOF-MS spectrum for **1**. 4
S2. ^{1}H NMR (500 MHz, C_5D_5N) spectrum for **1**. 4
S3. Enlarged ^{1}H NMR (500 MHz, C_5D_5N) spectrum 1 for **1**. 5
S4. Enlarged ^{1}H NMR (500 MHz, C_5D_5N) spectrum 2 for **1**. 5
S5. ^{13}C NMR (125 MHz, C_5D_5N) spectrum for **1**. 6
S6. ^{1}H ^{1}H COSY (C_5D_5N) spectrum for **1**. 6
S7. HSQC (C_5D_5N) spectrum for **1**. 7
S8. HMBC (C_5D_5N) spectrum for **1**. 7
S9. HRESI-TOF-MS spectrum for **2**. 8
S10. ^{1}H NMR (500 MHz, C_5D_5N) spectrum for **2**. 8
S11. Enlarged ^{1}H NMR (500 MHz, C_5D_5N) spectrum 1 for **2**. 9
S12. Enlarged ^{1}H NMR (500 MHz, C_5D_5N) spectrum 2 for **2**. 9
S13. ^{13}C NMR (125 MHz, C_5D_5N) spectrum for **2**. 10
S14. ^{1}H ^{1}H COSY (C_5D_5N) spectrum for **2**. 10
S15. HSQC (C_5D_5N) spectrum for **2**. 11
S16. HMBC (C_5D_5N) spectrum for **2**. 11
S17. HRESI-TOF-MS spectrum for **3**. 12
S18. ^{1}H NMR (500 MHz, C_5D_5N) spectrum for **3**. 12
S19. Enlarged ^{1}H NMR (500 MHz, C_5D_5N) spectrum 1 for **3**. 13
S20. Enlarged ^{1}H NMR (500 MHz, C_5D_5N) spectrum 2 for **3**. 13
S21. ^{13}C NMR (125 MHz, C_5D_5N) spectrum for **3**. 14
S22. ^{1}H ^{1}H COSY (C_5D_5N) spectrum for **3**. 14
S23. HSQC (C_5D_5N) spectrum for **3**. 15
S24. HMBC (C_5D_5N) spectrum for **3**. 15
S25. HRESI-TOF-MS spectrum for **4**. 16
S26. ^{1}H NMR (500 MHz, C_5D_5N) spectrum for **4**. 16
S27. ^{13}C NMR (125 MHz, C_5D_5N) spectrum for **4**. 17
S28. Enlarged ^{13}C NMR (125 MHz, C_5D_5N) spectrum for **4**. 17
S29. ^{1}H ^{1}H COSY (C_5D_5N) spectrum for **4**. 18
S30. HSQC (C_5D_5N) spectrum for **4**. 18
S31. HMBC (C_5D_5N) spectrum for **4**. 19
S32. HRESI-TOF-MS spectrum for **5**. 20
S33. ^{1}H NMR (500 MHz, C_5D_5N) spectrum for **5**. 20
S34. Enlarged ^{1}H NMR (500 MHz, C_5D_5N) spectrum 1 for **5**. 21
S35. Enlarged ^{1}H NMR (500 MHz, C_5D_5N) spectrum 2 for **5**. 21
S36. ^{13}C NMR (125 MHz, C_5D_5N) spectrum for **5**. 22
S37. Enlarged ^{13}C NMR (125 MHz, C_5D_5N) spectrum for **5**. 22
S38. ^{1}H ^{1}H COSY (C_5D_5N) spectrum for **5**. 23
S39. HSQC (C_5D_5N) spectrum for **5**. 23
S40. HMBC (C_5D_5N) spectrum for **5**. 24
S41. HSQC-TOCSY (C_5D_5N) spectrum for **5**. 24
S42. HRESI-TOF-MS spectrum for **6**. 25
S43. ^{1}H NMR (500 MHz, C_5D_5N) spectrum for **6**. 25

S44. Enlarged ^{1}H NMR (500 MHz, C_5D_5N) spectrum 1 for **6**. 26
S45. Enlarged ^{1}H NMR (500 MHz, C_5D_5N) spectrum 2 for **6**. 26
S46. ^{13}C NMR (125 MHz, C_5D_5N) spectrum for **6**. 27
S47. Enlarged ^{13}C NMR (125 MHz, C_5D_5N) spectrum for **6**. 27
S48. ^{1}H ^{1}H COSY (C_5D_5N) spectrum for **6**. 28
S49. HSQC (C_5D_5N) spectrum for **6**. 28
S50. HMBC (C_5D_5N) spectrum for **6**. 29
S51. HRESI-TOF-MS spectrum for **7**. 30
S52. ^{1}H NMR (500 MHz, C_5D_5N) spectrum for **7**. 30
S53. Enlarged ^{1}H NMR (500 MHz, C_5D_5N) spectrum 1 for **7**. 31
S54. Enlarged ^{1}H NMR (500 MHz, C_5D_5N) spectrum 2 for **7**. 31
S55. ^{13}C NMR (125 MHz, C_5D_5N) spectrum for **7**. 32
S56. Enlarged ^{13}C NMR (125 MHz, C_5D_5N) spectrum for **7**. 32
S57. ^{1}H ^{1}H COSY (C_5D_5N) spectrum for **7**. 33
S58. HSQC (C_5D_5N) spectrum for **7**. 33
S59. HMBC (C_5D_5N) spectrum for **7**. 34
S60. HRESI-TOF-MS spectrum for **8**. 35
S61. ^{1}H NMR (500 MHz, C_5D_5N) spectrum for **8**. 35
S62. Enlarged ^{1}H NMR (500 MHz, C_5D_5N) spectrum 1 for **8**. 36
S63. Enlarged ^{1}H NMR (500 MHz, C_5D_5N) spectrum 2 for **8**. 36
S64. ^{13}C NMR (125 MHz, C_5D_5N) spectrum for **8**. 37
S65. Enlarged ^{13}C NMR (125 MHz, C_5D_5N) spectrum for **8**. 37
S66. ^{1}H ^{1}H COSY (C_5D_5N) spectrum for **8**. 38
S67. HSQC (C_5D_5N) spectrum for **8**. 38
S68. HMBC (C_5D_5N) spectrum for **8**. 39
S69. HSQC-TOCSY (C_5D_5N) spectrum for **8**. 39
S70. HRESI-TOF-MS spectrum for **9**. 40
S71. ^{1}H NMR (500 MHz, C_5D_5N) spectrum for **9**. 40
S72. ^{13}C NMR (125 MHz, C_5D_5N) spectrum for **9**. 41
S73. Enlarged ^{13}C NMR (125 MHz, C_5D_5N) spectrum for **9**. 41
S74. ^{1}H ^{1}H COSY (C_5D_5N) spectrum for **9**. 42
S75. HSQC (C_5D_5N) spectrum for **9**. 42
S76. HMBC (C_5D_5N) spectrum for **9**. 43
S77. HSQC-TOCSY (C_5D_5N) spectrum for **9**. 43

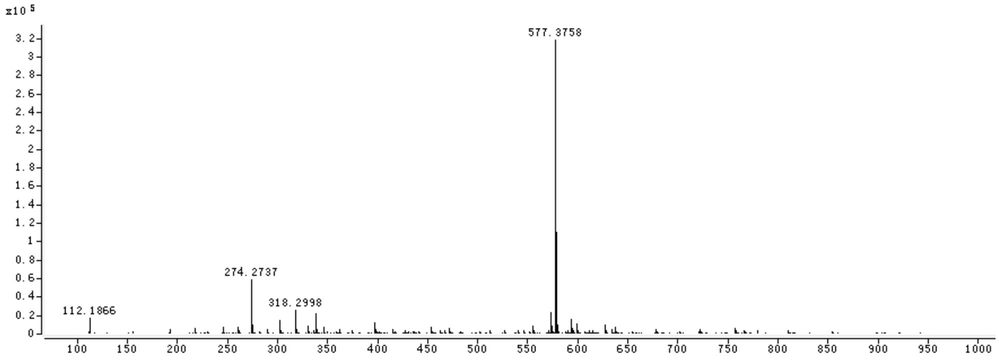

S1. HRESI-TOF-MS spectrum for **1**.

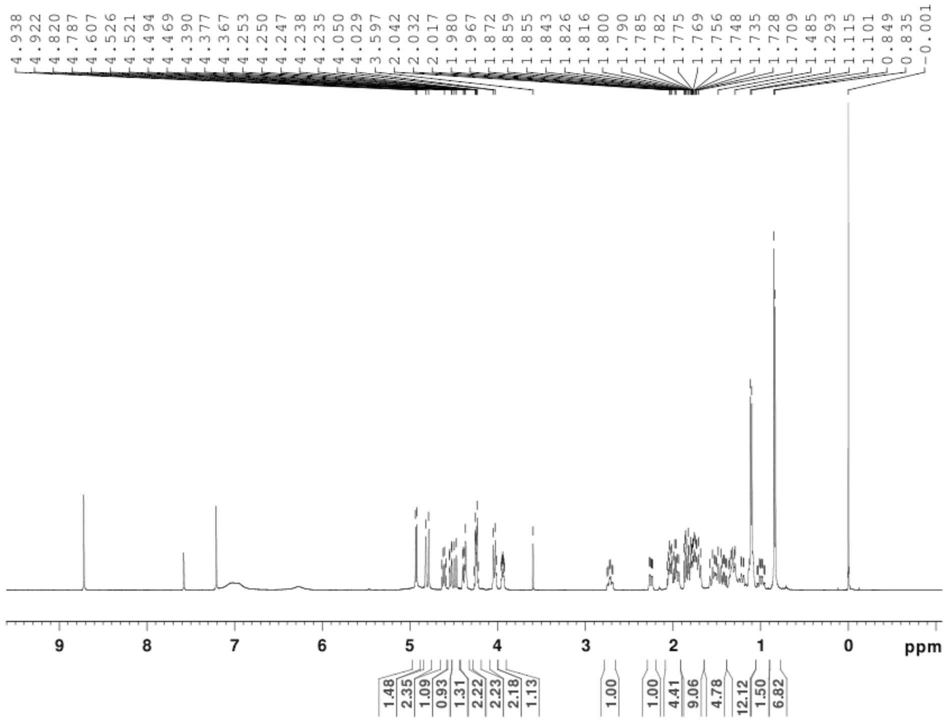

S2. ^{1}H NMR (500 MHz, C_5D_5N) spectrum for **1**.

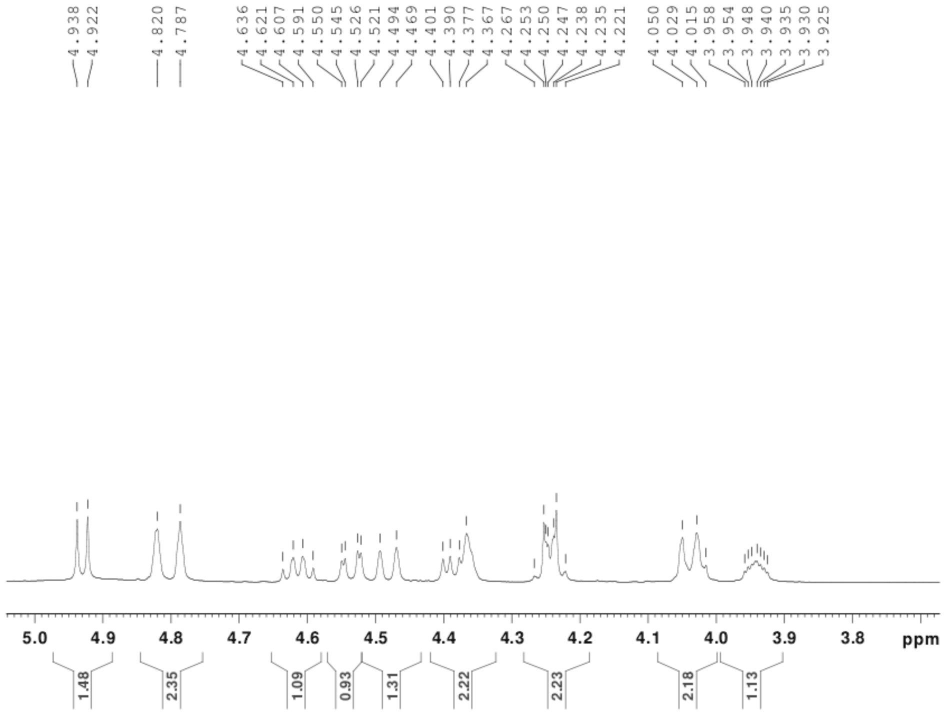

S3. Enlarged ^{1}H NMR (500 MHz, C_5D_5N) spectrum 1 for **1**.

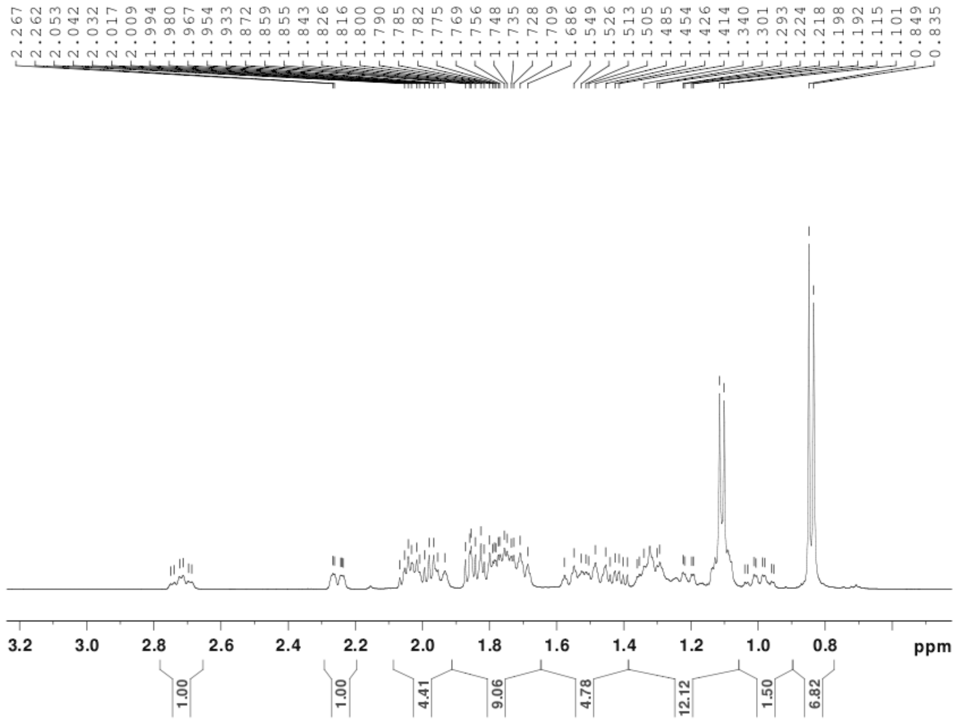

S4. Enlarged ^{1}H NMR (500 MHz, C_5D_5N) spectrum 2 for **1**.

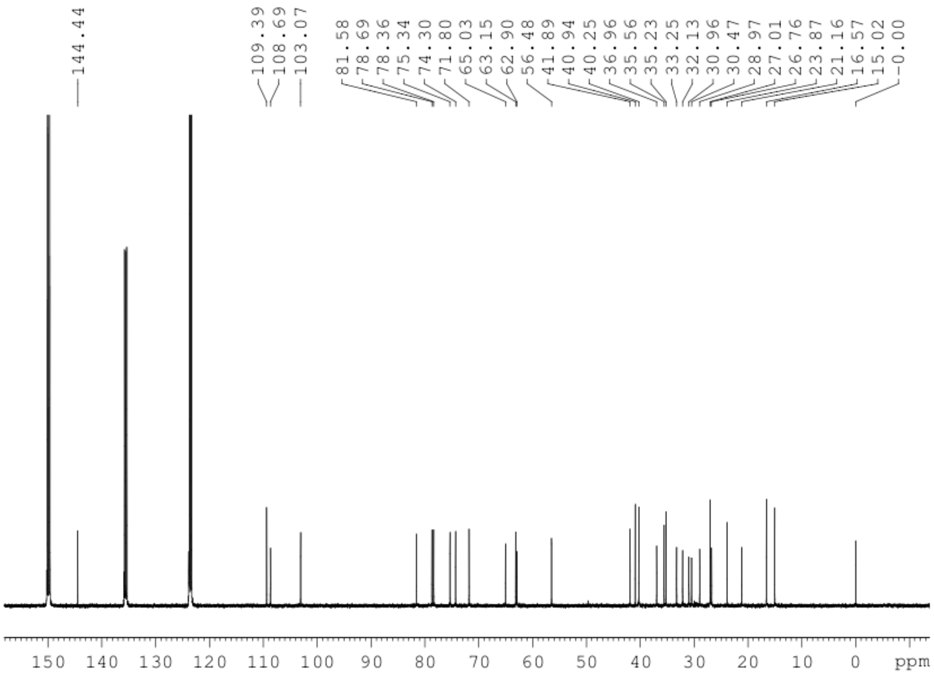

S5. ^{13}C NMR (125 MHz, C_5D_5N) spectrum for **1**.

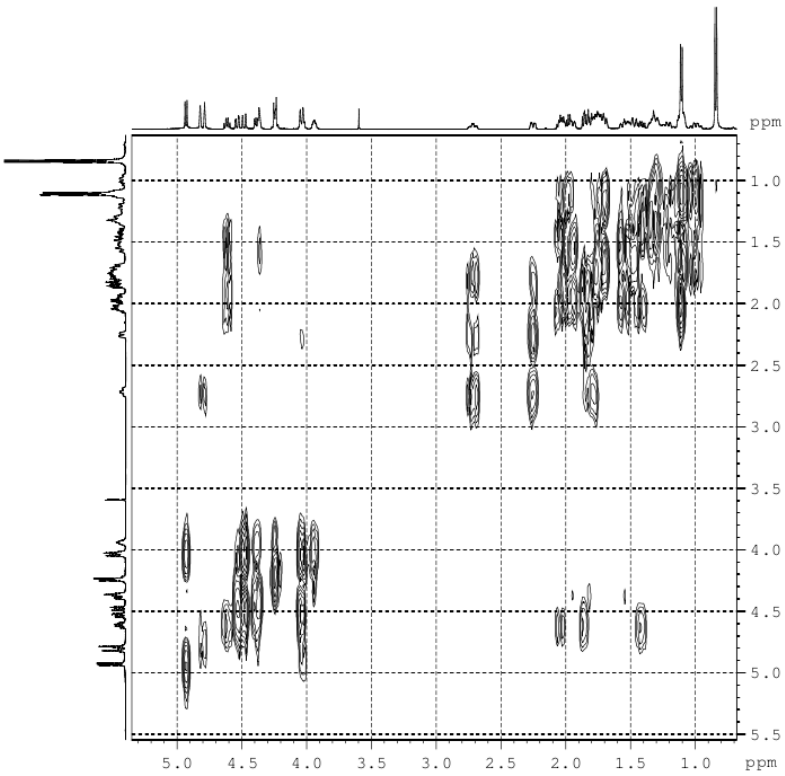

S6. ^{1}H ^{1}H COSY (C_5D_5N) spectrum for **1**.

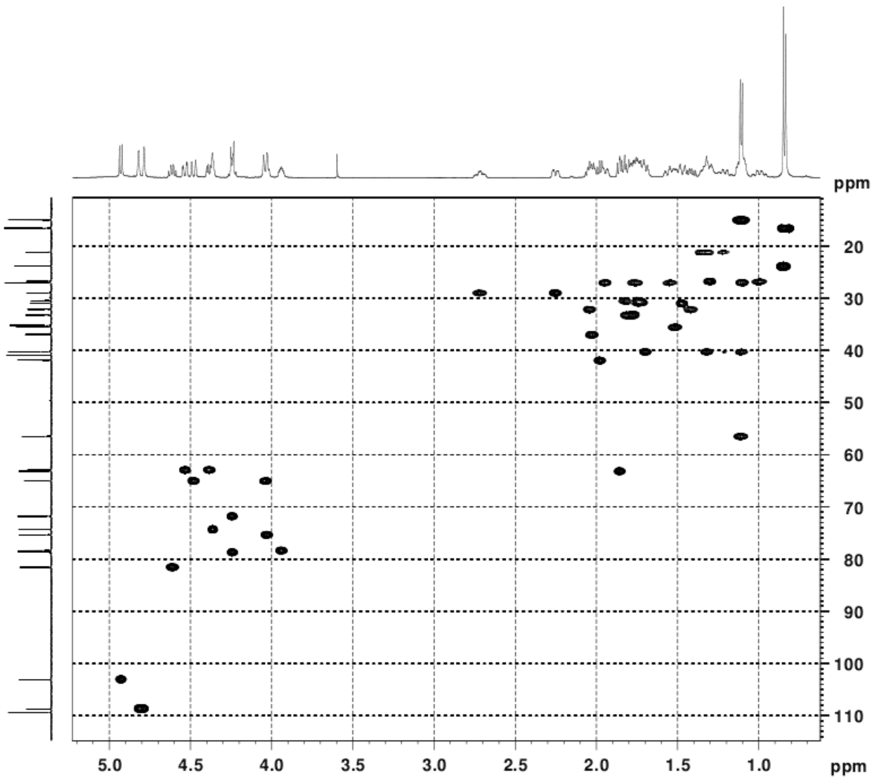

S7. HSQC (C_5D_5N) spectrum for **1**.

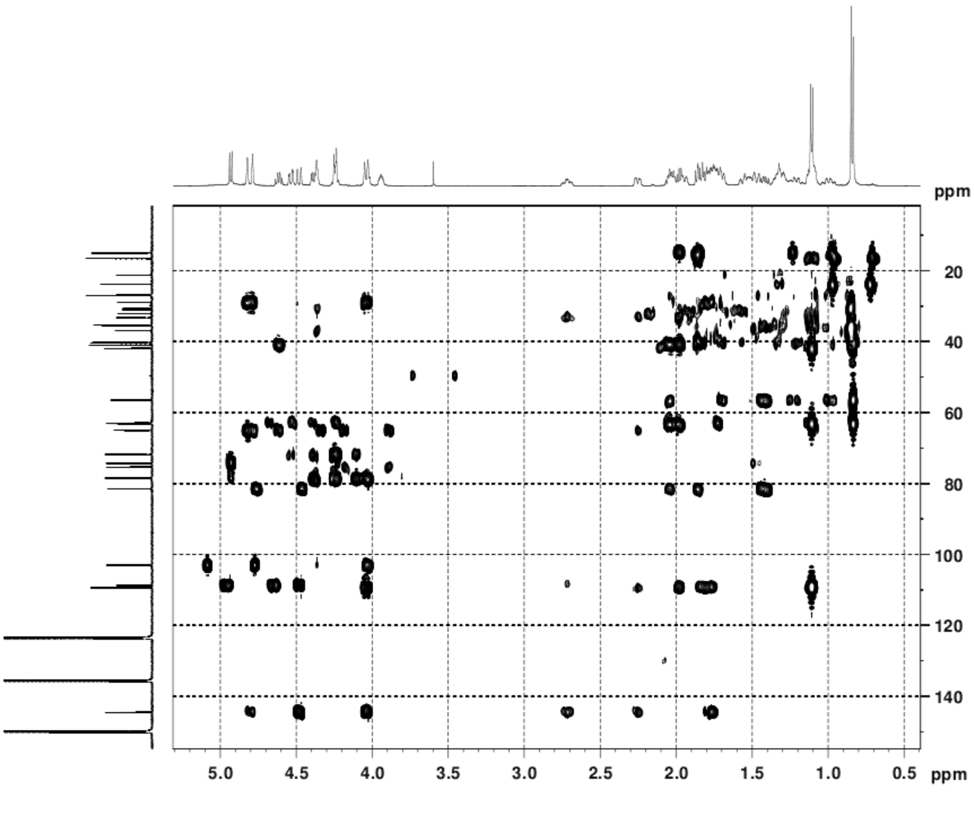

S8. HMBC (C_5D_5N) spectrum for **1**.

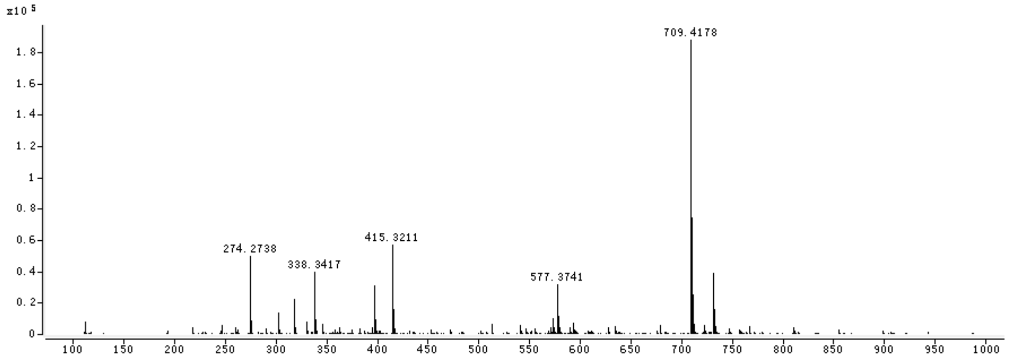

S9. HRESI-TOF-MS spectrum for **2**.

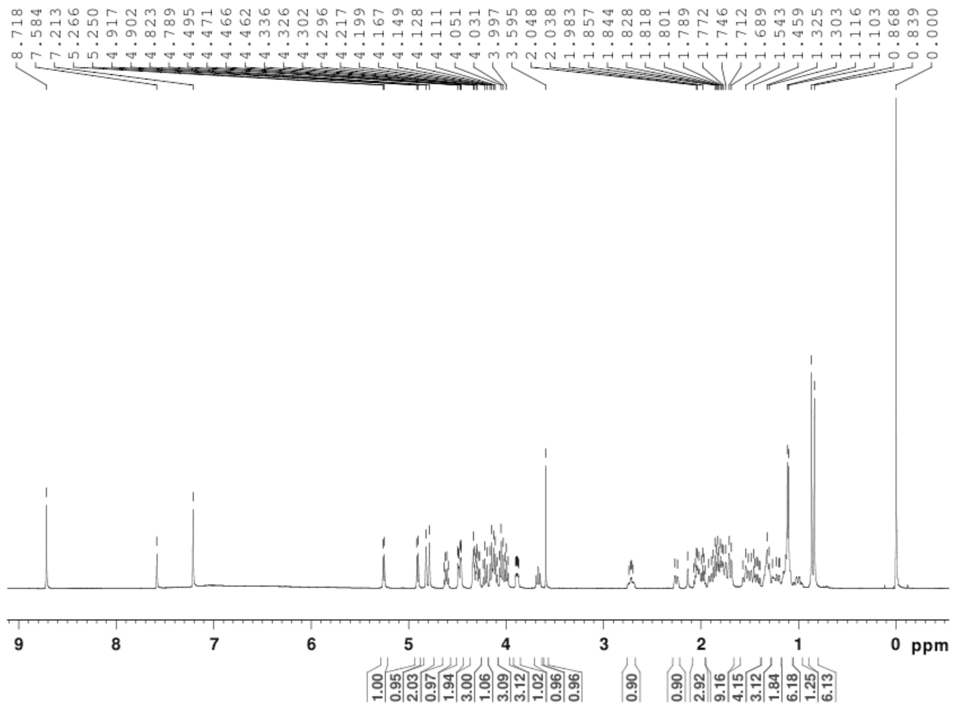

S10. ^{1}H NMR (500 MHz, C_5D_5N) spectrum for **2**.

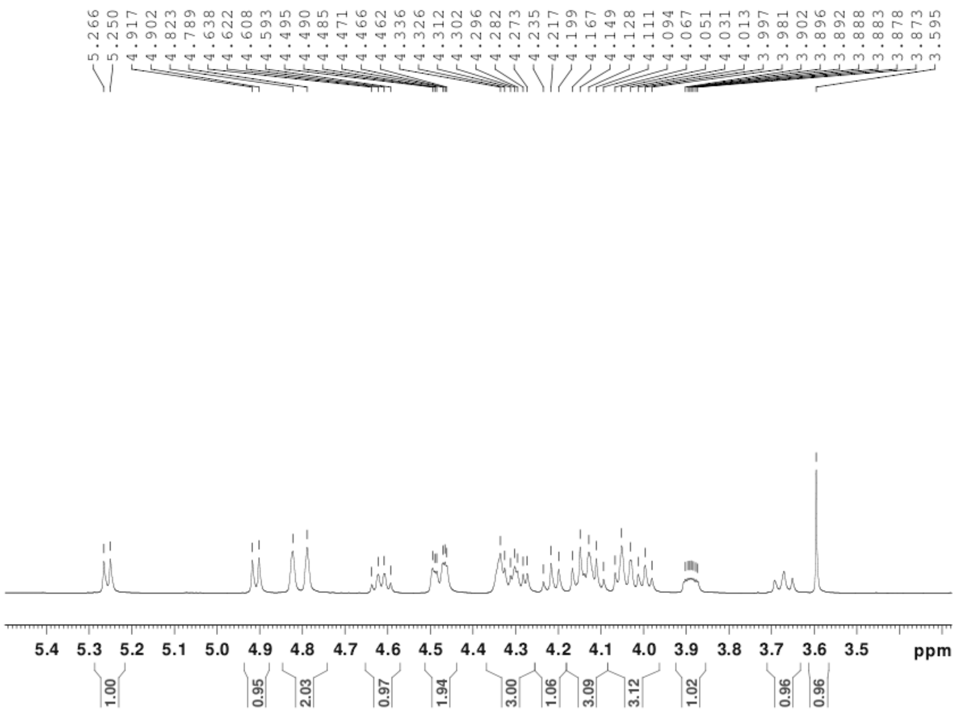

S11. Enlarged ^{1}H NMR (500 MHz, C_5D_5N) spectrum 1 for **2**.

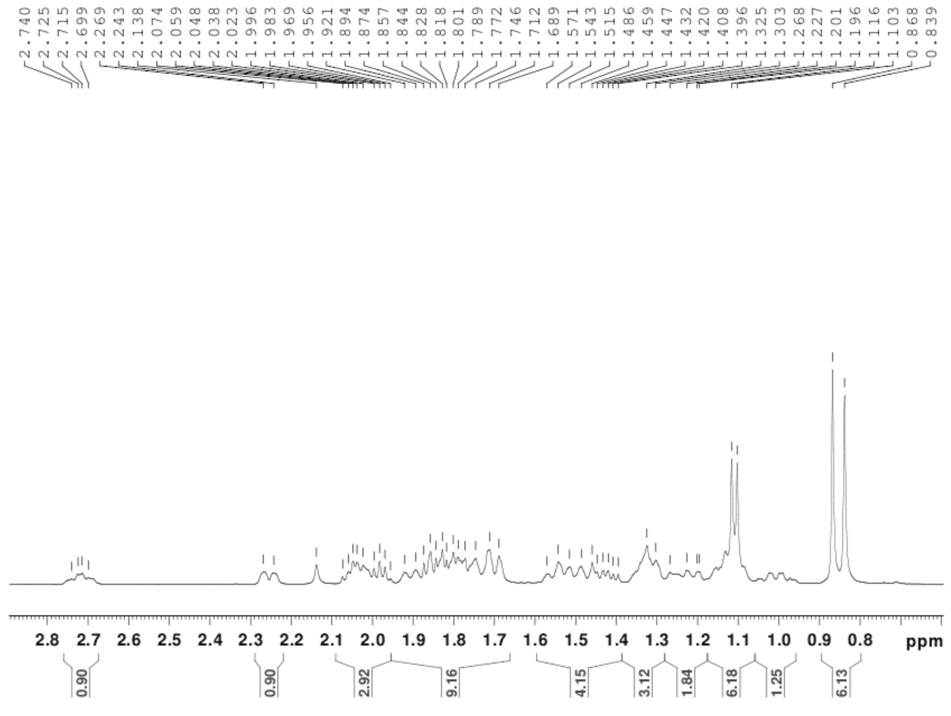

S12. Enlarged ^{1}H NMR (500 MHz, C_5D_5N) spectrum 2 for **2**.

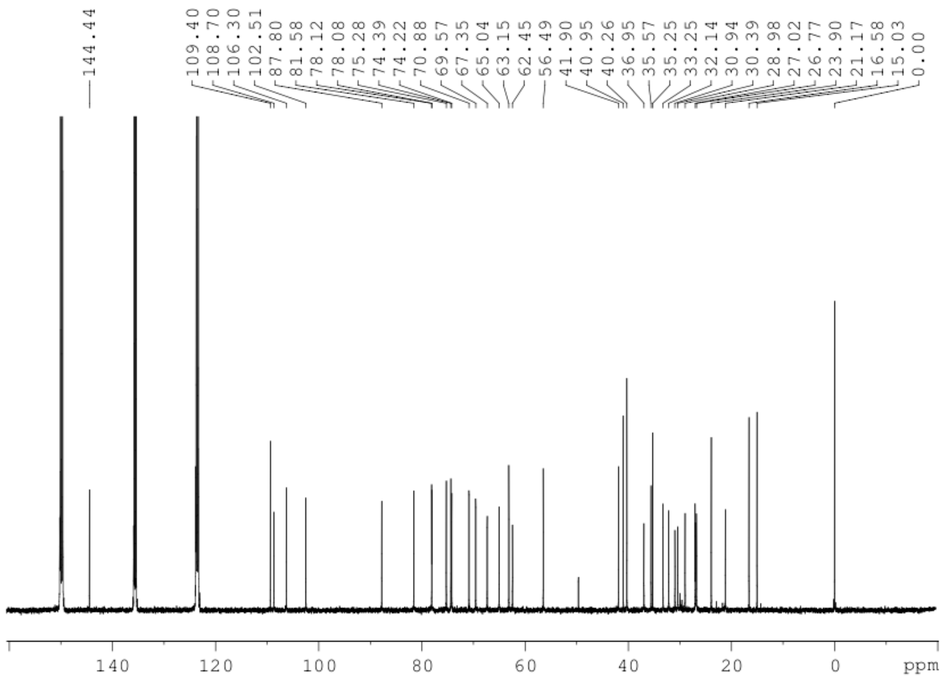

S13. ^{13}C NMR (125 MHz, C_5D_5N) spectrum for **2**.

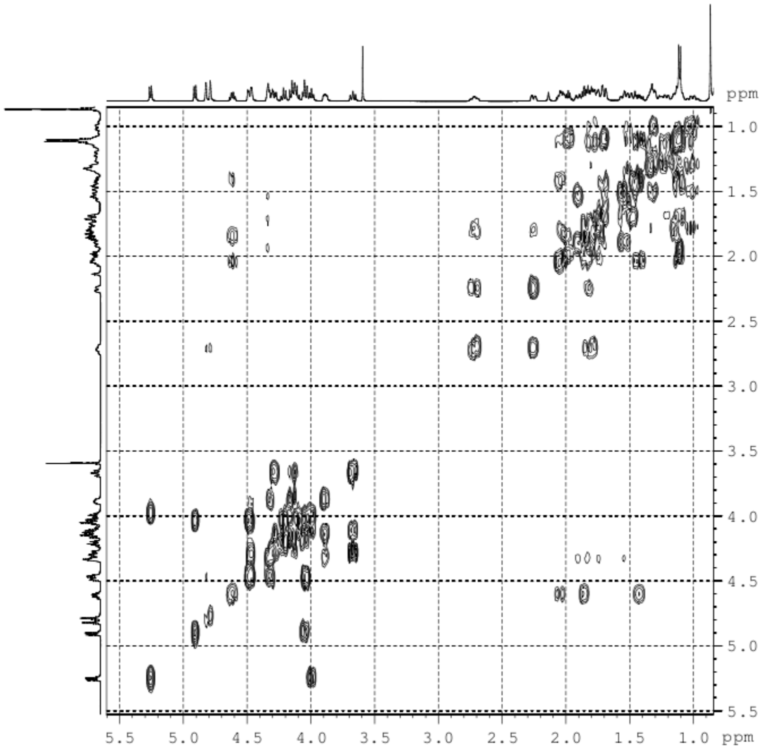

S14. ^{1}H ^{1}H COSY (C_5D_5N) spectrum for **2**.

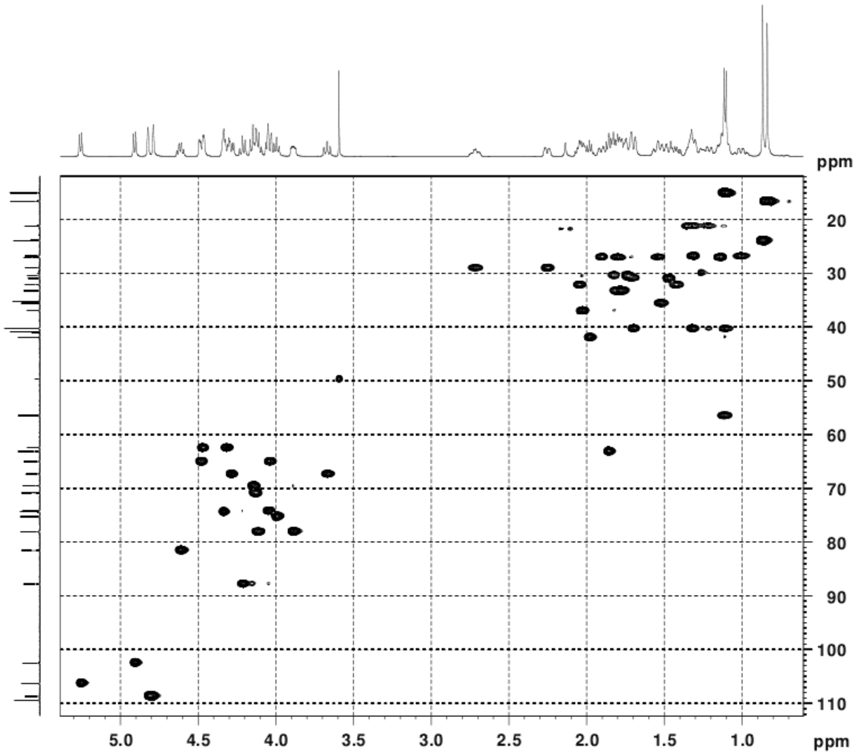

S15. HSQC (C_5D_5N) spectrum for **2**.

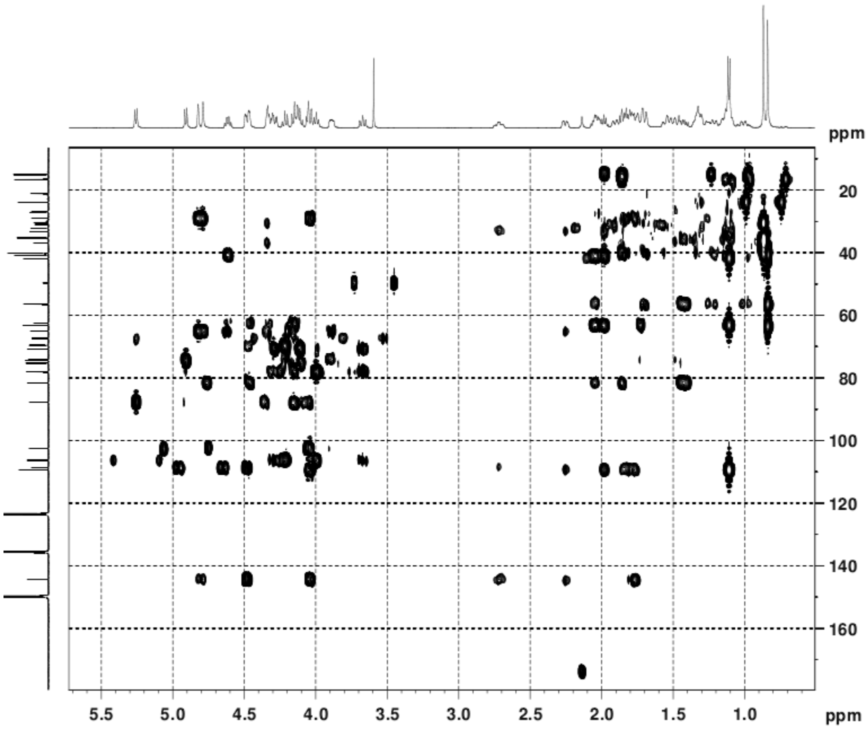

S16. HMBC (C_5D_5N) spectrum for **2**.

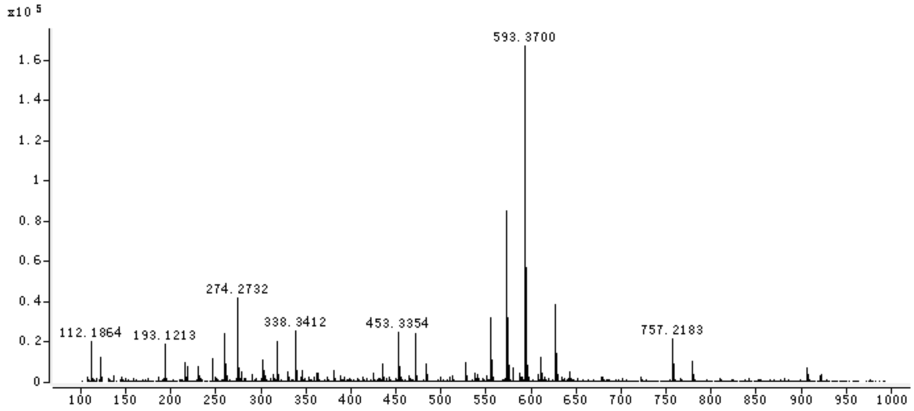

S17. HRESI-TOF-MS spectrum for **3**.

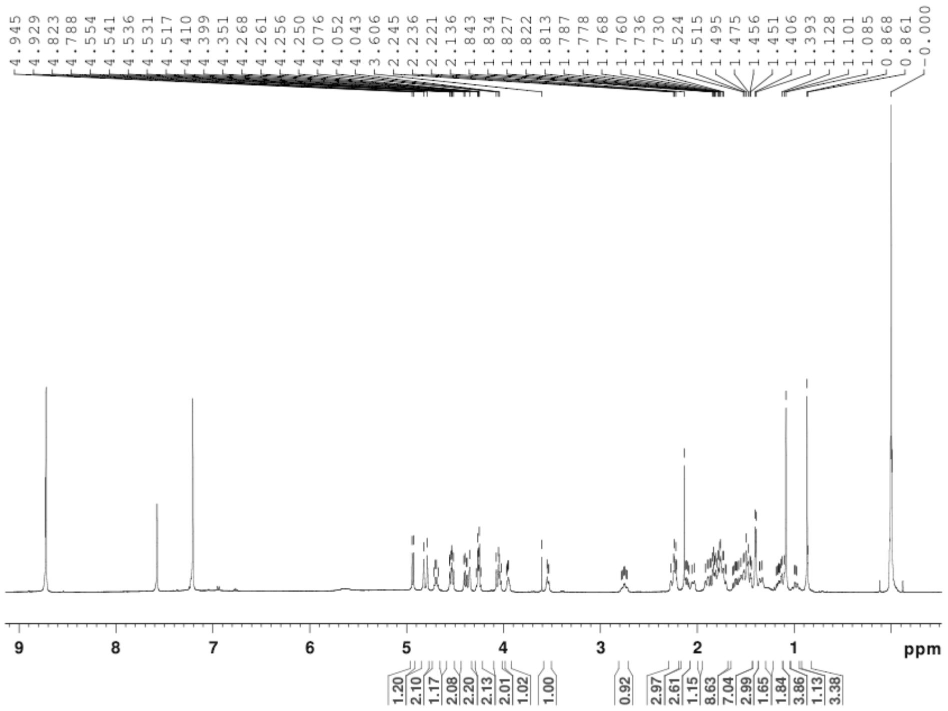

S18. ^{1}H NMR (500 MHz, C_5D_5N) spectrum for **3**.

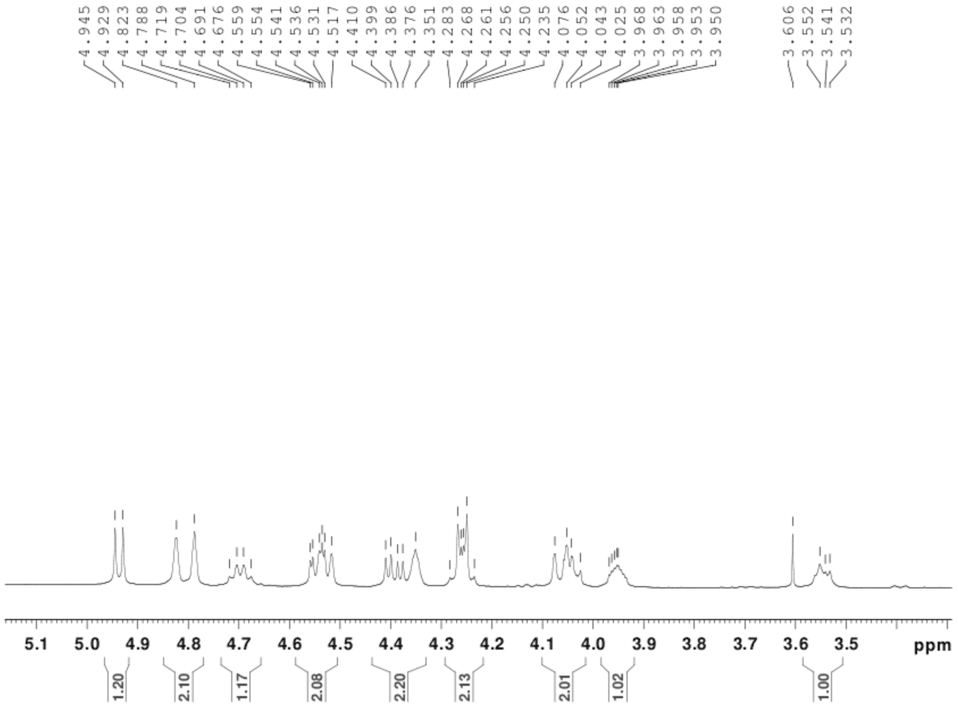

S19. Enlarged ^{1}H NMR (500 MHz, C_5D_5N) spectrum 1 for **3**.

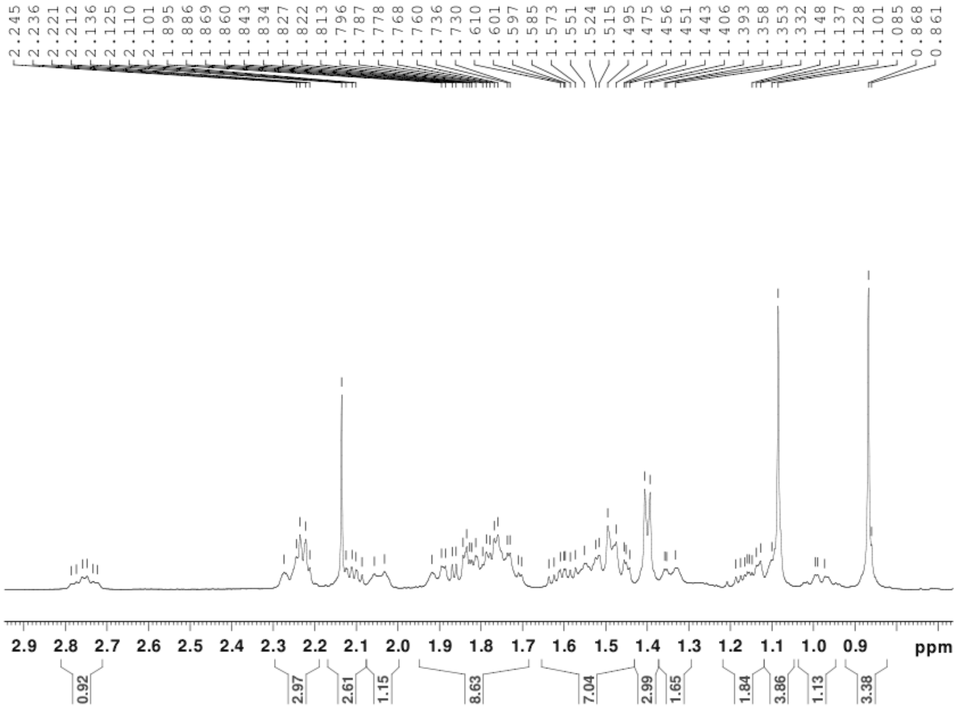

S20. Enlarged ^{1}H NMR (500 MHz, C_5D_5N) spectrum 2 for **3**.

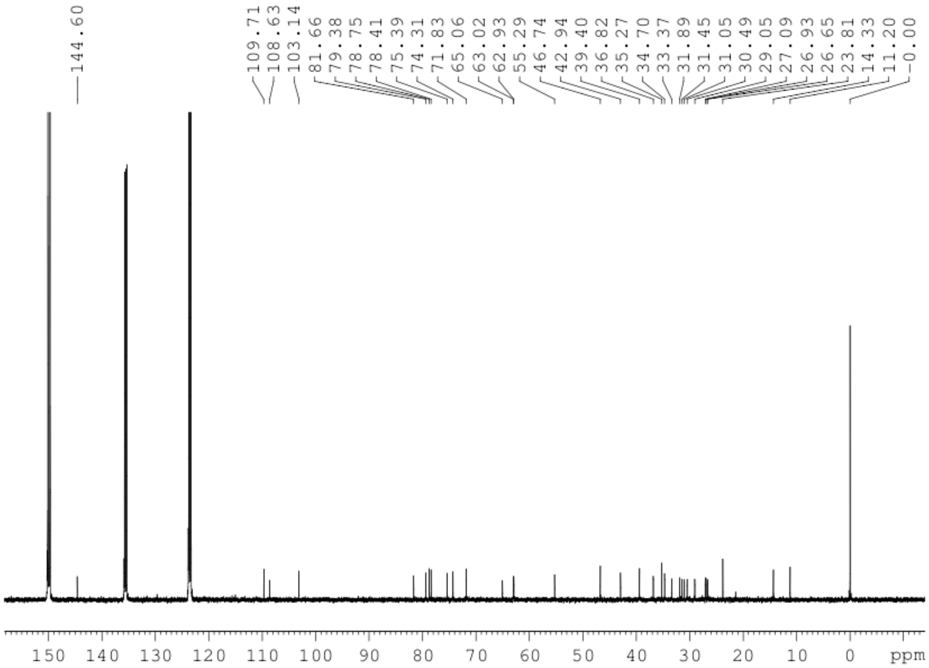

S21. ^{13}C NMR (125 MHz, C_5D_5N) spectrum for **3**.

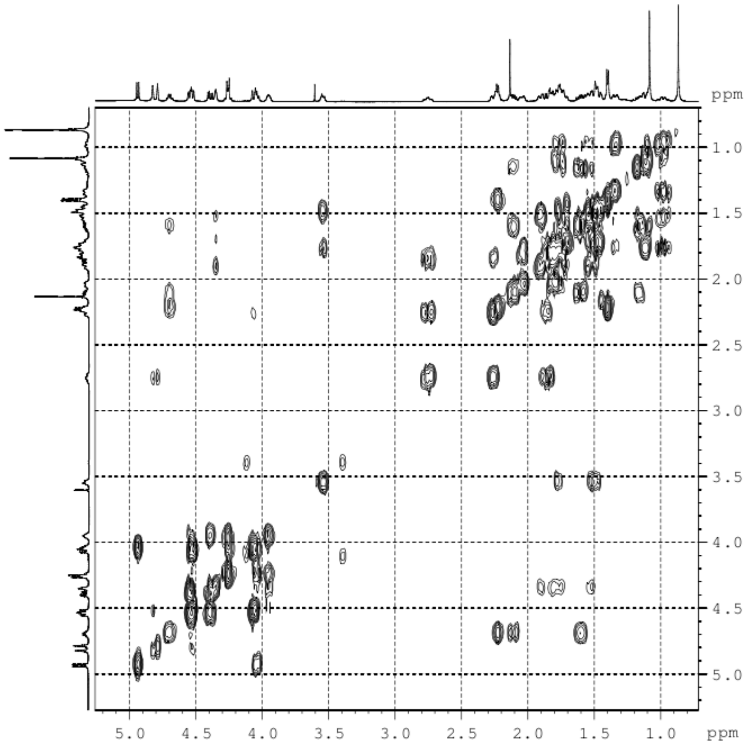

S22. ^{1}H ^{1}H COSY (C_5D_5N) spectrum for **3**.

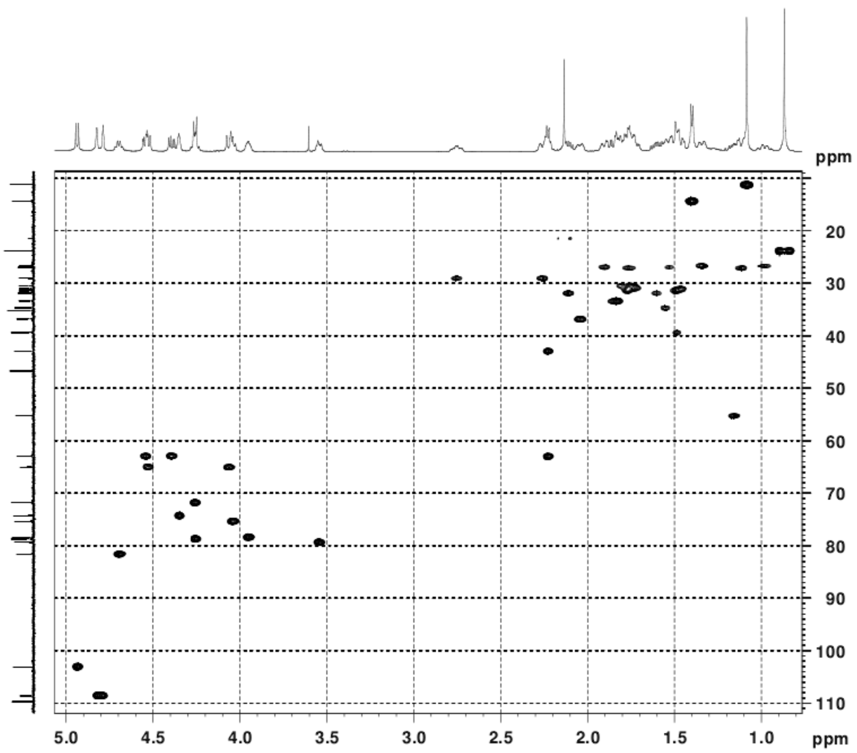

S23. HSQC (C_5D_5N) spectrum for **3**.

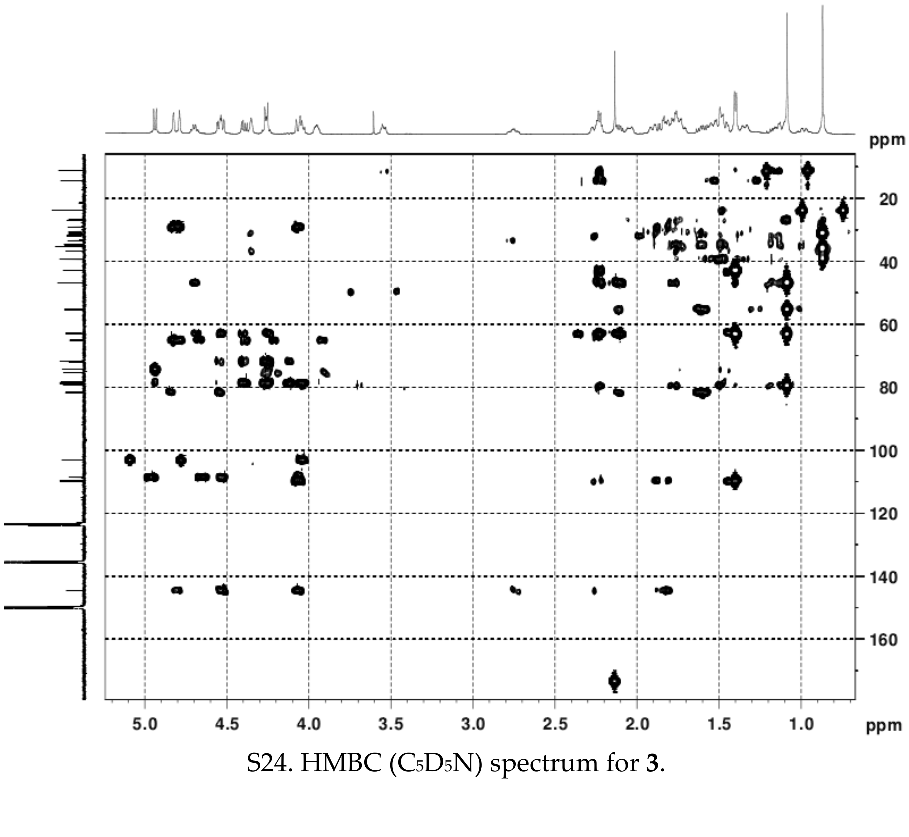

S24. HMBC (C_5D_5N) spectrum for **3**.

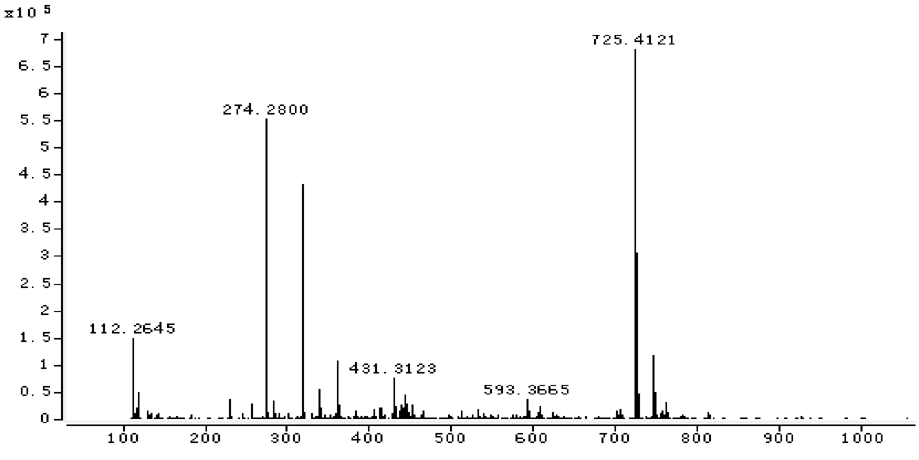

S25. HRESI-TOF-MS spectrum for **4**.

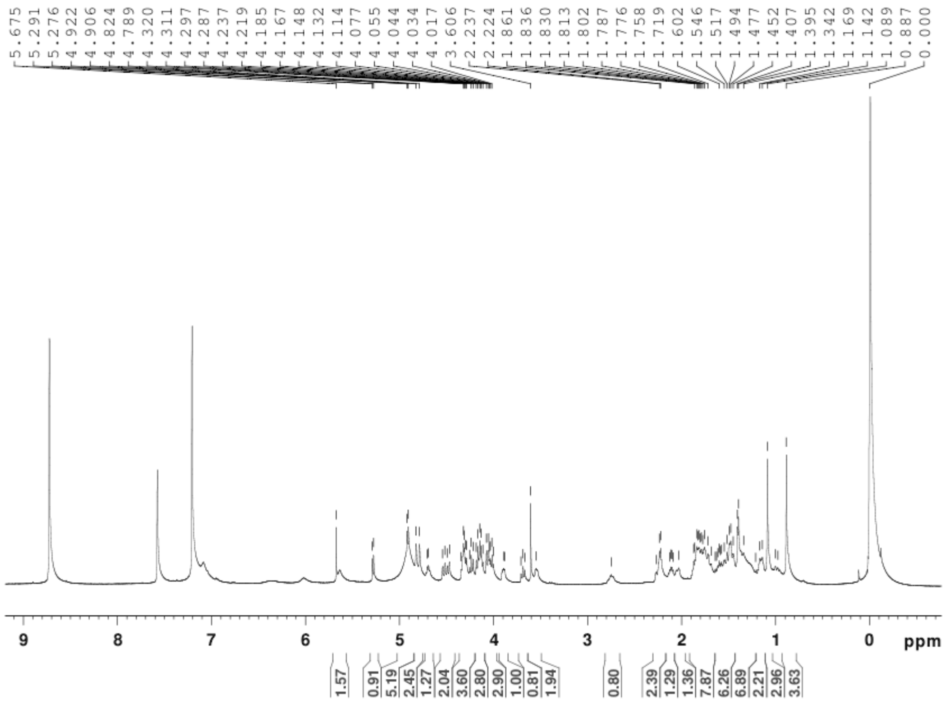

S26. ^{1}H NMR (500 MHz, C_5D_5N) spectrum for **4**.

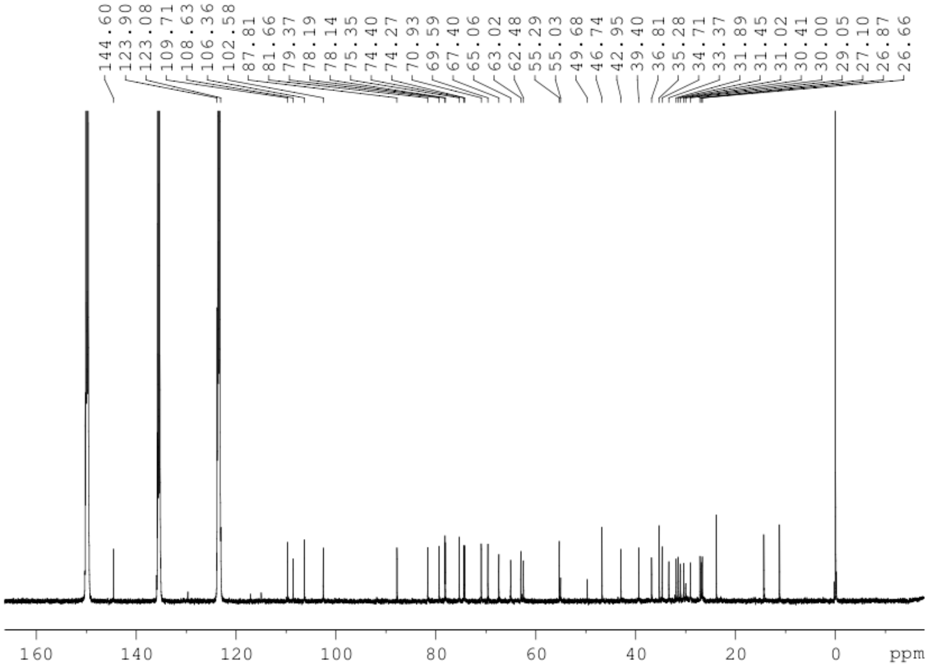

S27. ^{13}C NMR (125 MHz, C_5D_5N) spectrum for **4**.

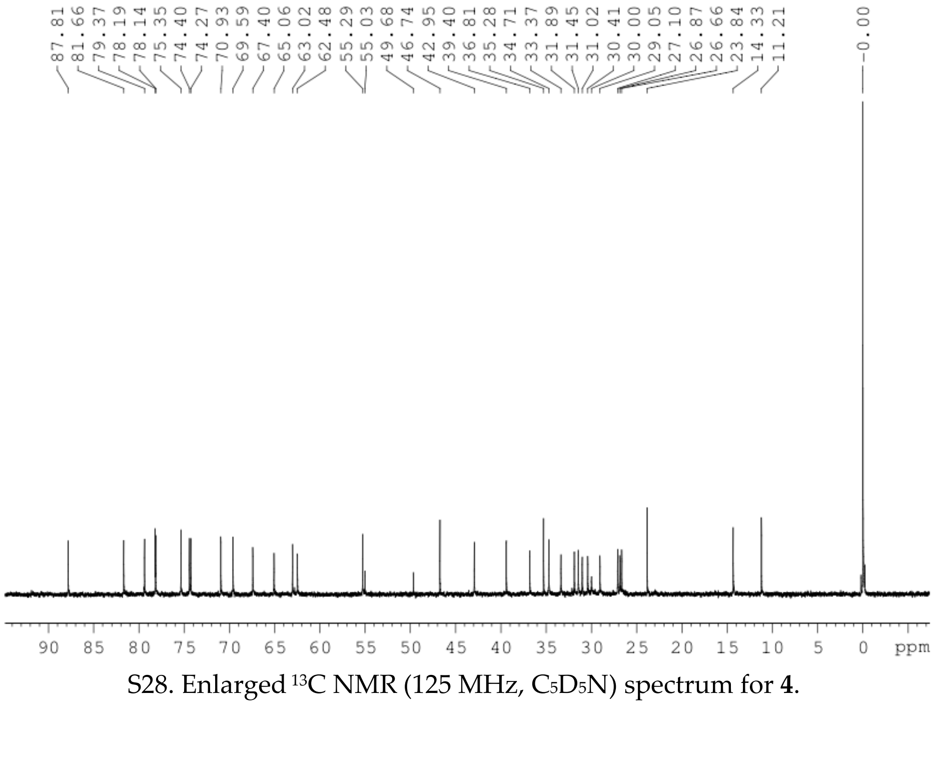

S28. Enlarged ^{13}C NMR (125 MHz, C_5D_5N) spectrum for **4**.

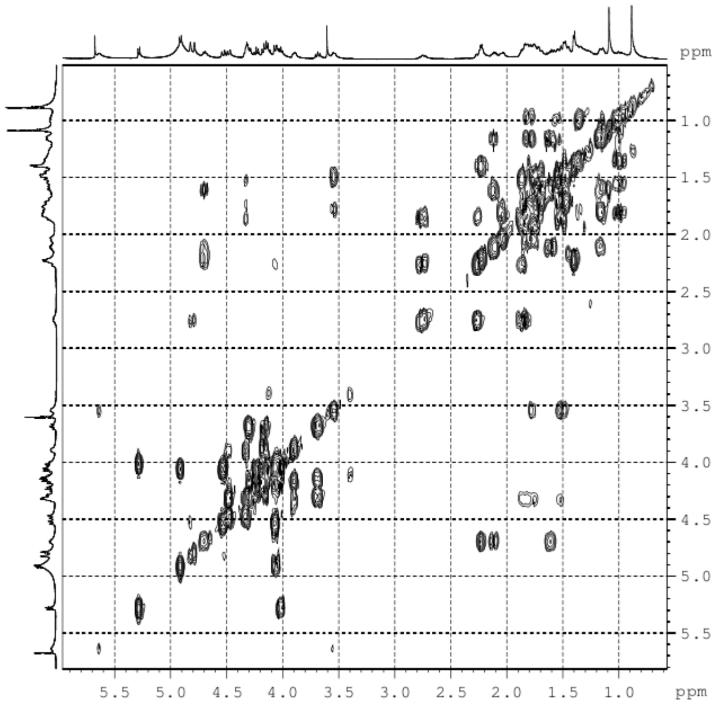

S29. ^1H ^1H COSY (C_5D_5N) spectrum for **4**.

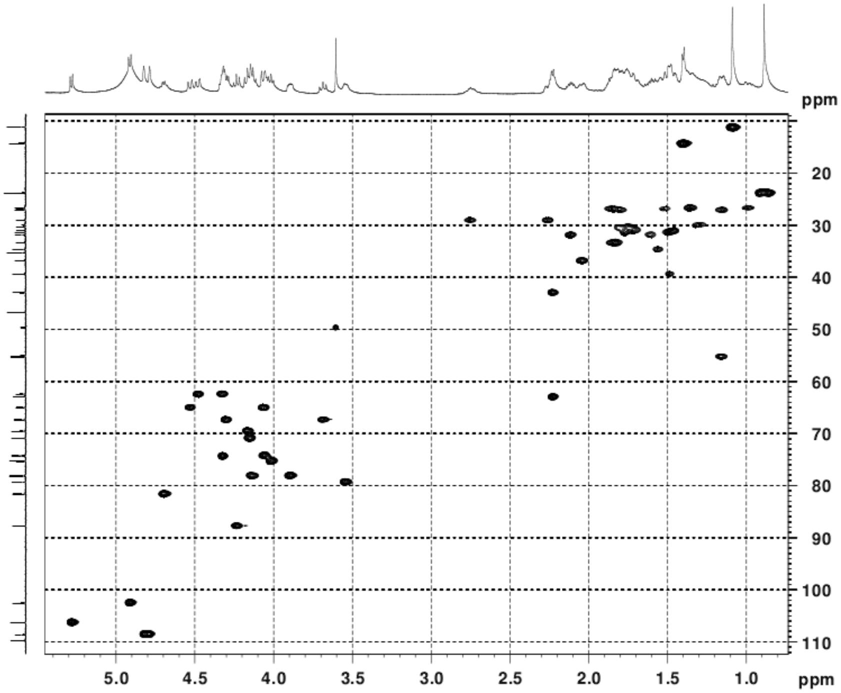

S30. HSQC (C_5D_5N) spectrum for **4**.

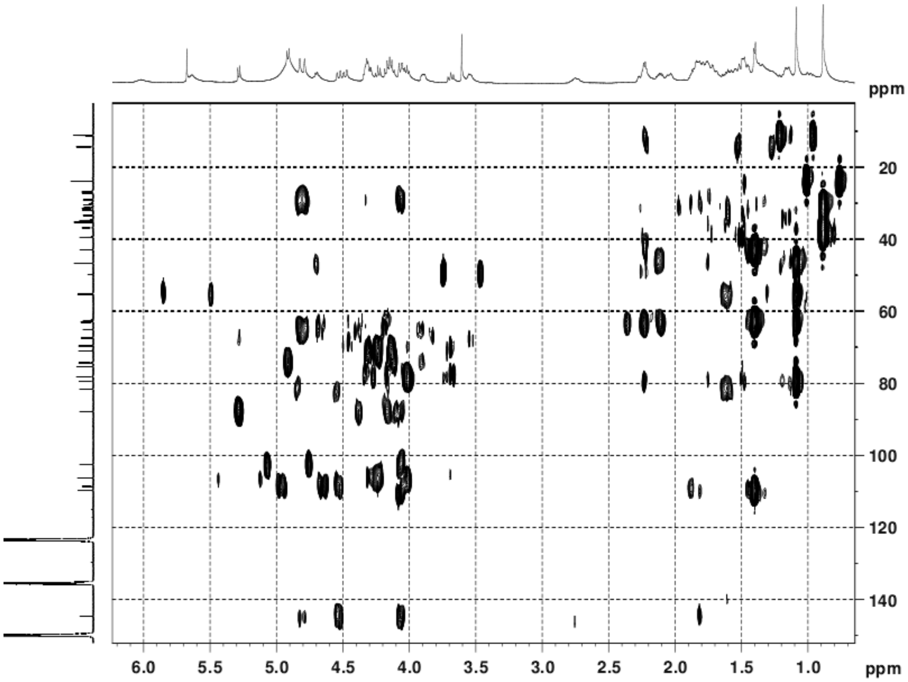

S31. HMBC (C_5D_5N) spectrum for **4**.

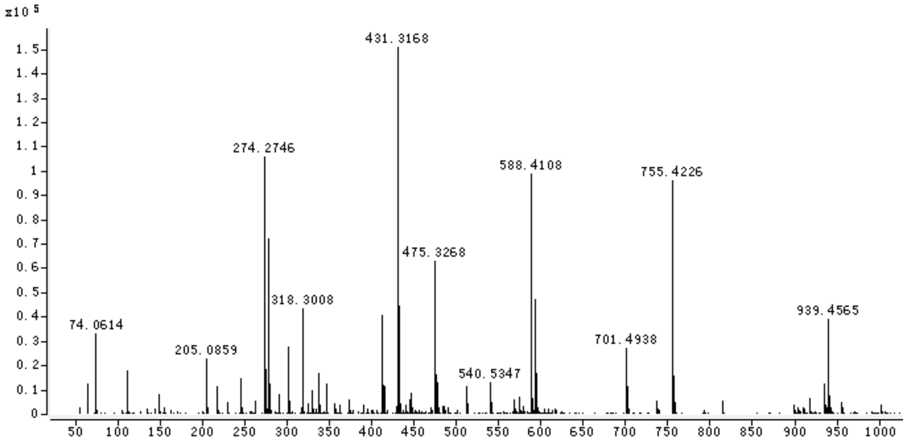

S32. HRESI-TOF-MS spectrum for **5**.

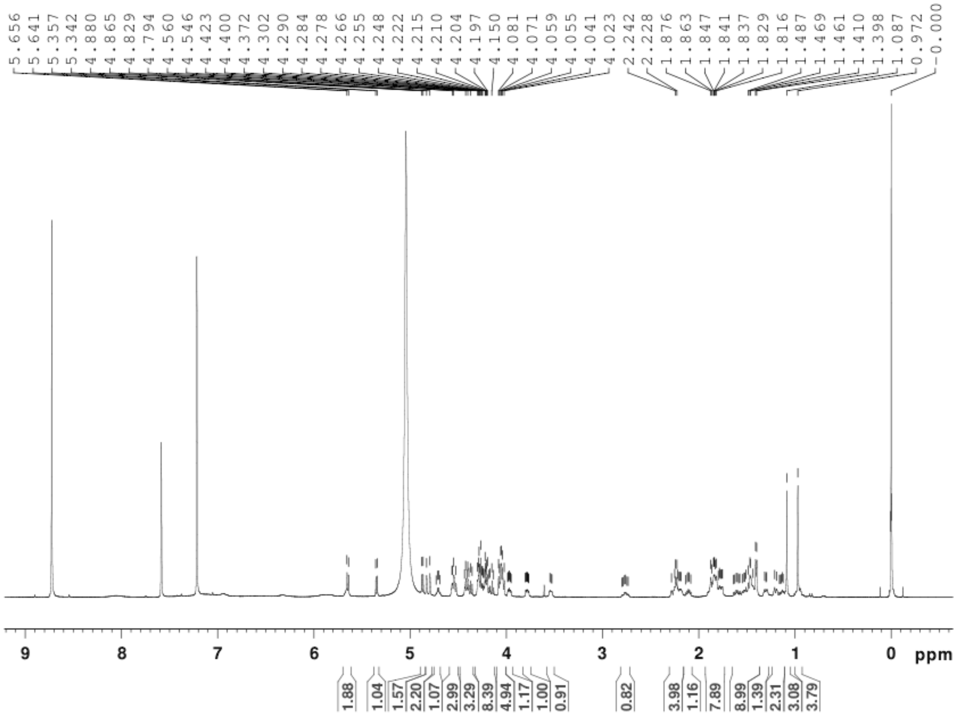

S33. ^{1}H NMR (500 MHz, C_5D_5N) spectrum for **5**.

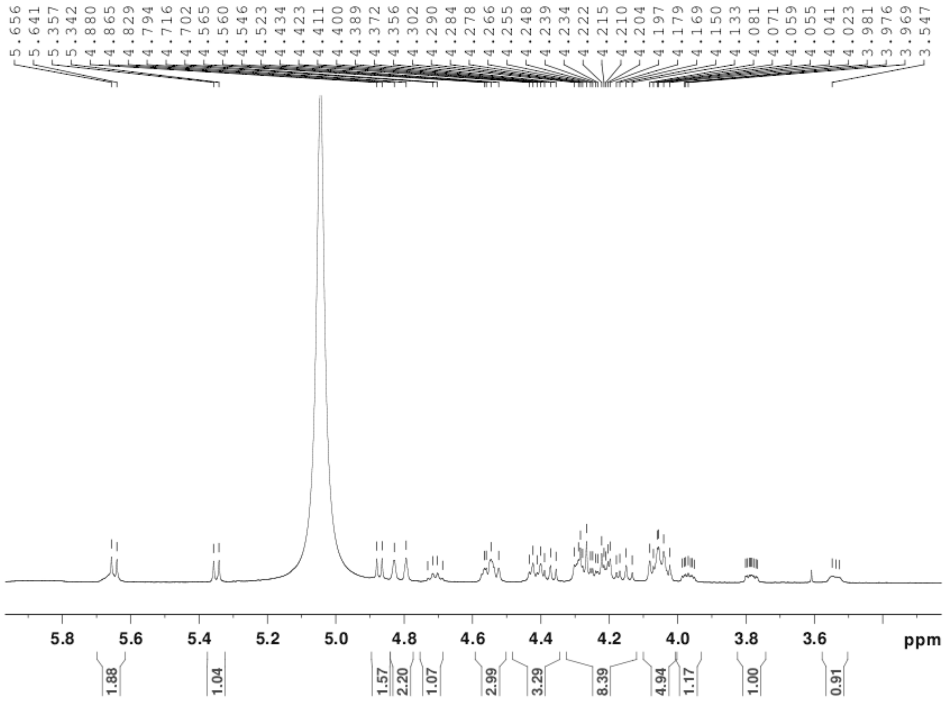

S34. Enlarged ^{1}H NMR (500 MHz, C_5D_5N) spectrum 1 for **5**.

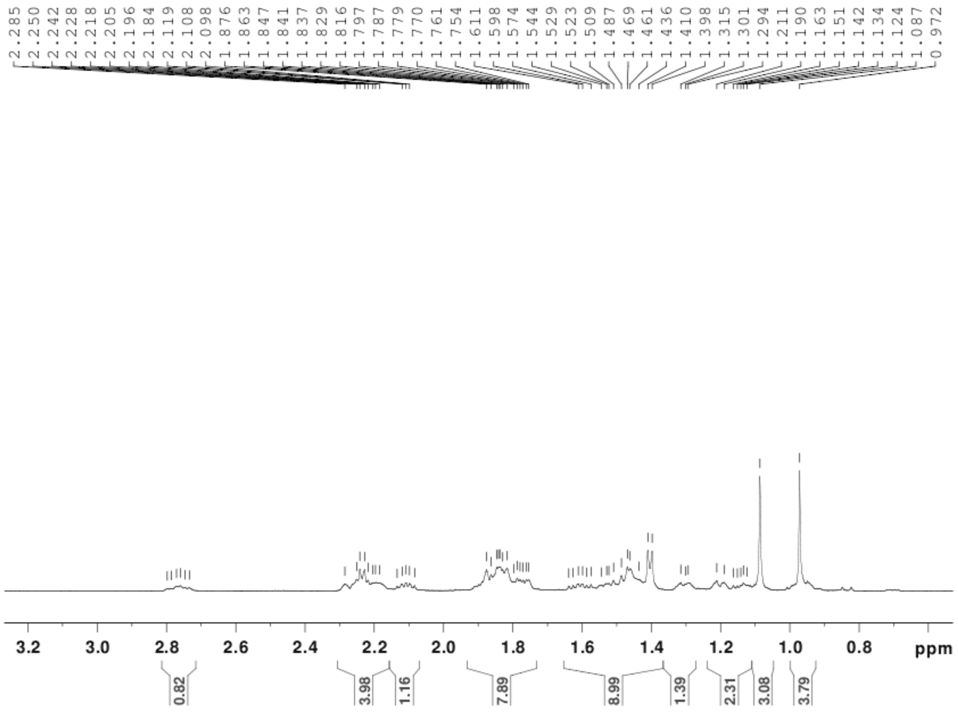

S35. Enlarged ^{1}H NMR (500 MHz, C_5D_5N) spectrum 2 for **5**.

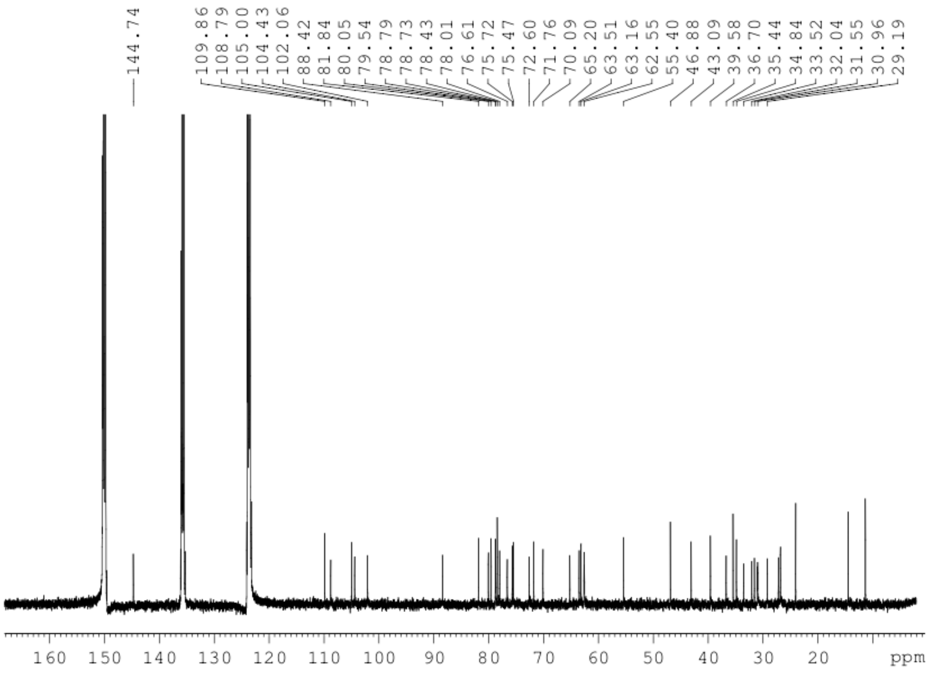

S36. ^{13}C NMR (125 MHz, C_5D_5N) spectrum for **5**.

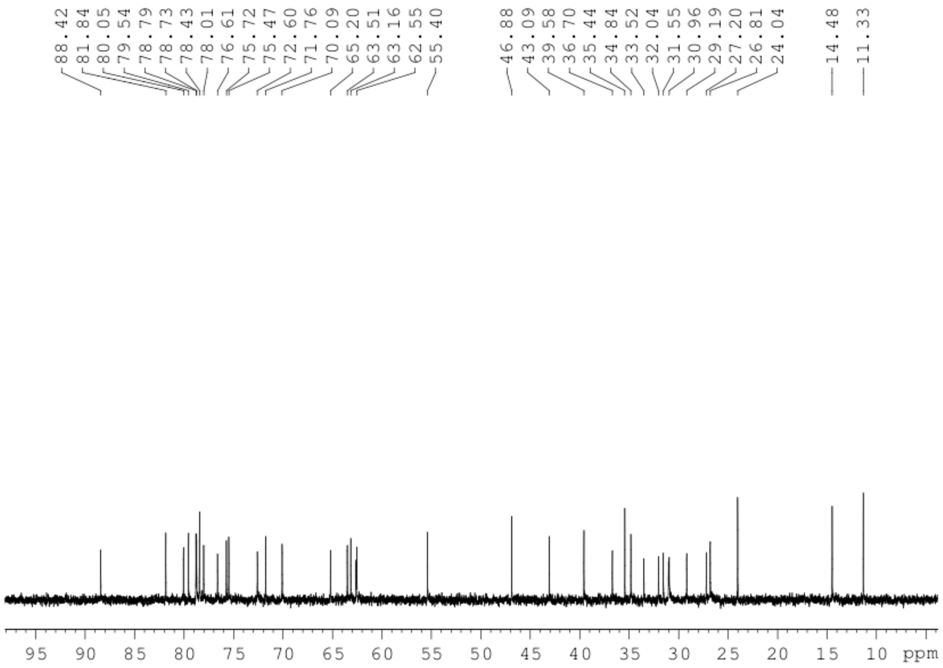

S37. Enlarged ^{13}C NMR (125 MHz, C_5D_5N) spectrum for **5**.

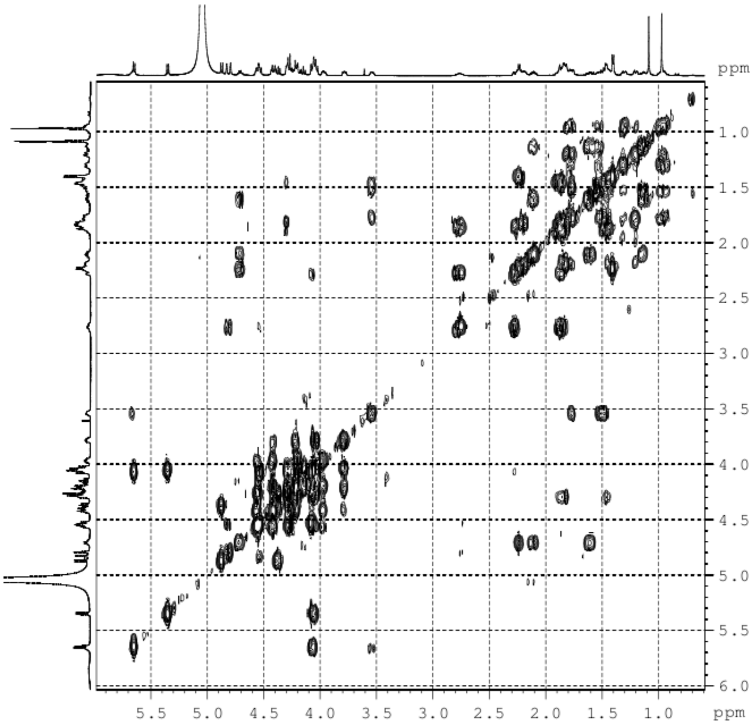

S38. ^{1}H ^{1}H COSY (C_5D_5N) spectrum for **5**.

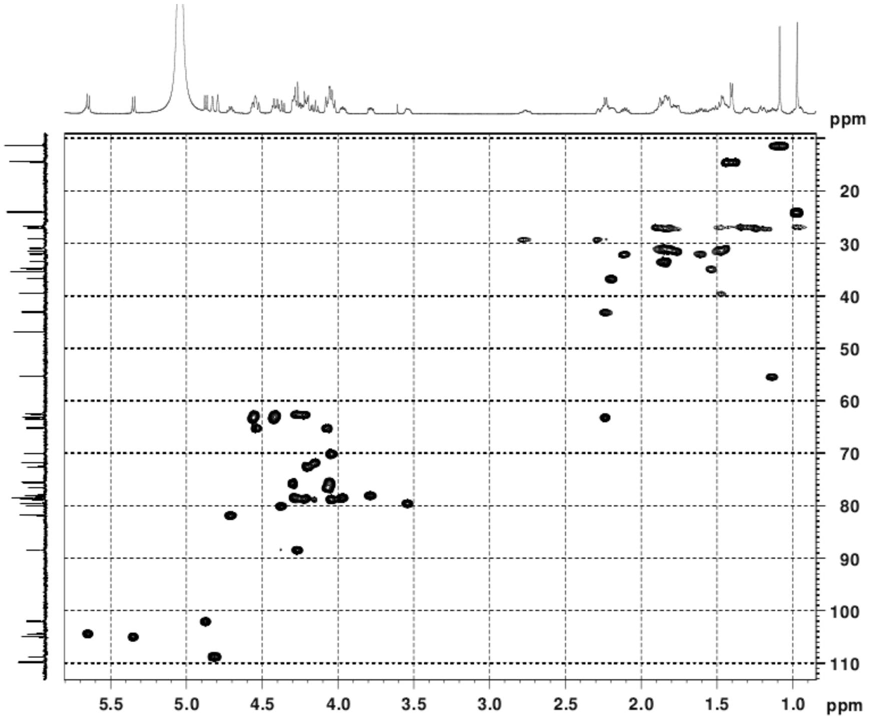

S39. HSQC (C_5D_5N) spectrum for **5**.

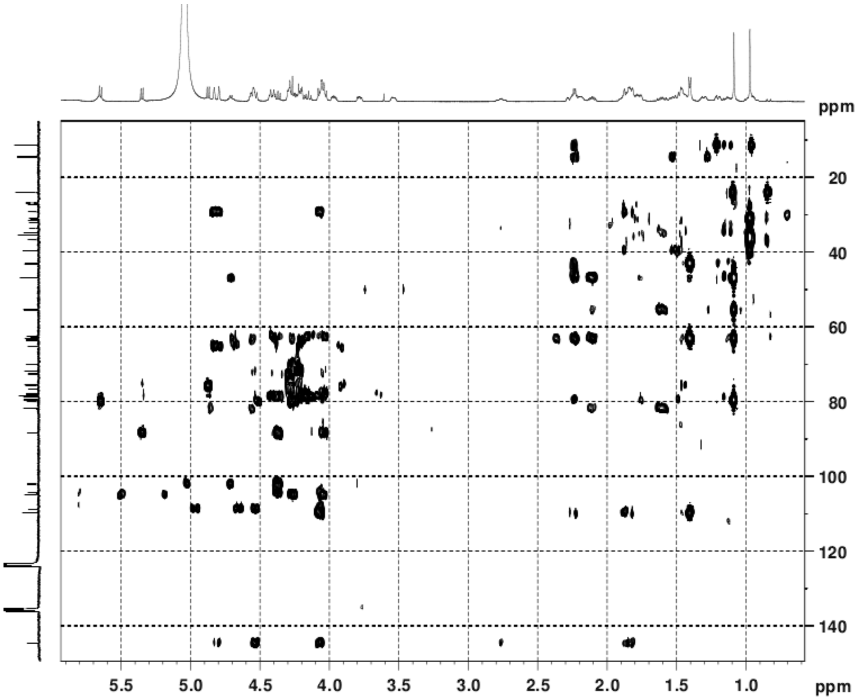

S40. HMBC (C_5D_5N) spectrum for **5**.

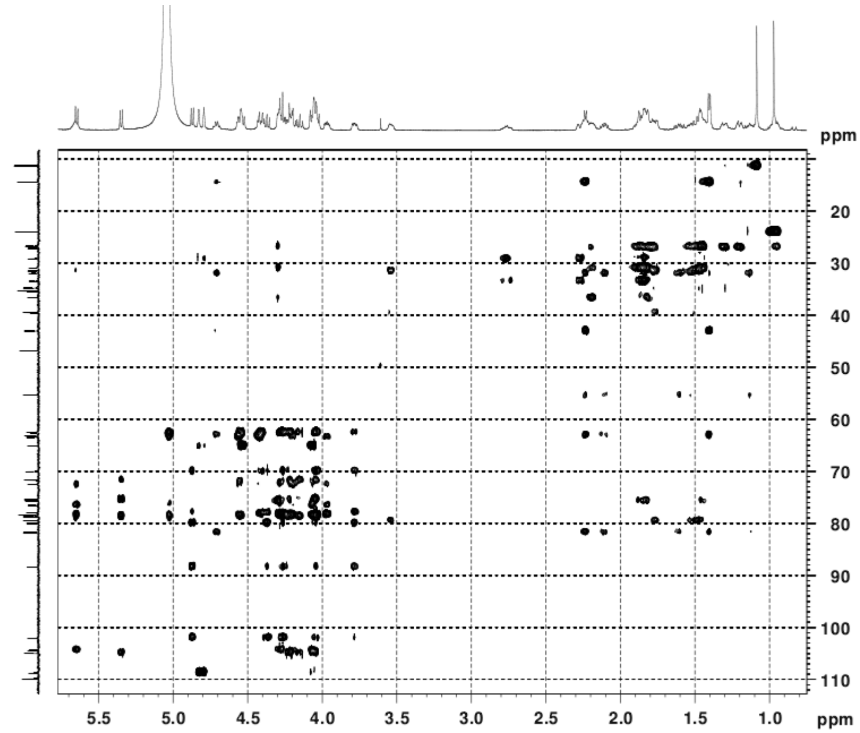

S41. HSQC-TOCSY (C_5D_5N) spectrum for **5**.

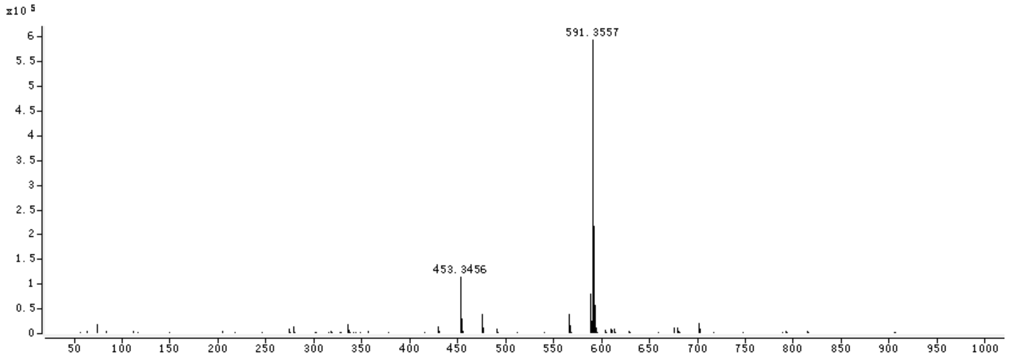

S42. HRESI-TOF-MS spectrum for **6**.

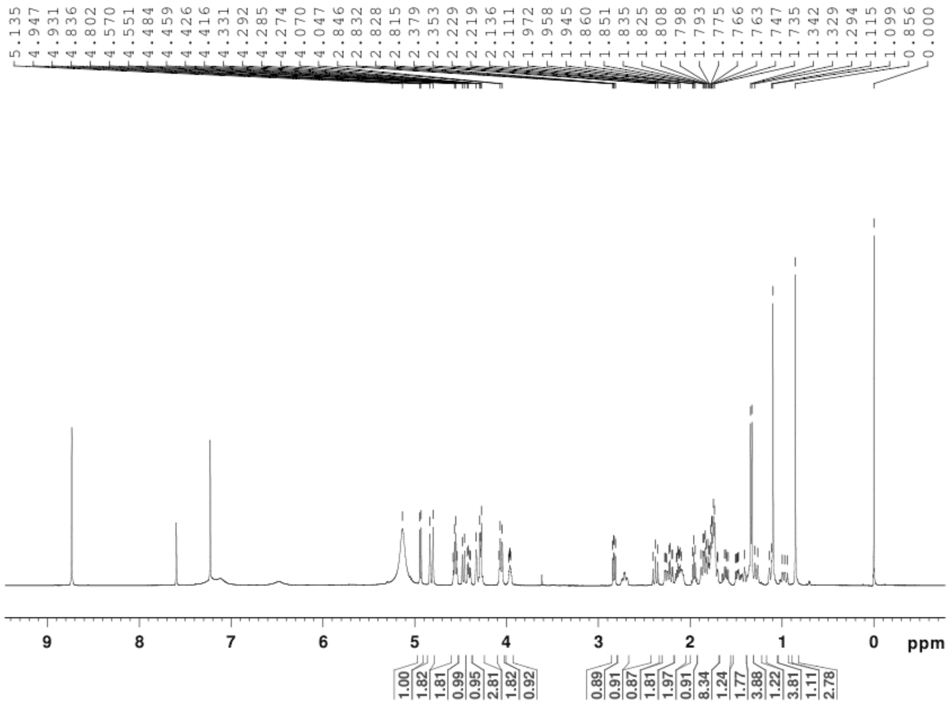

S43. ^{1}H NMR (500 MHz, C_5D_5N) spectrum for **6**.

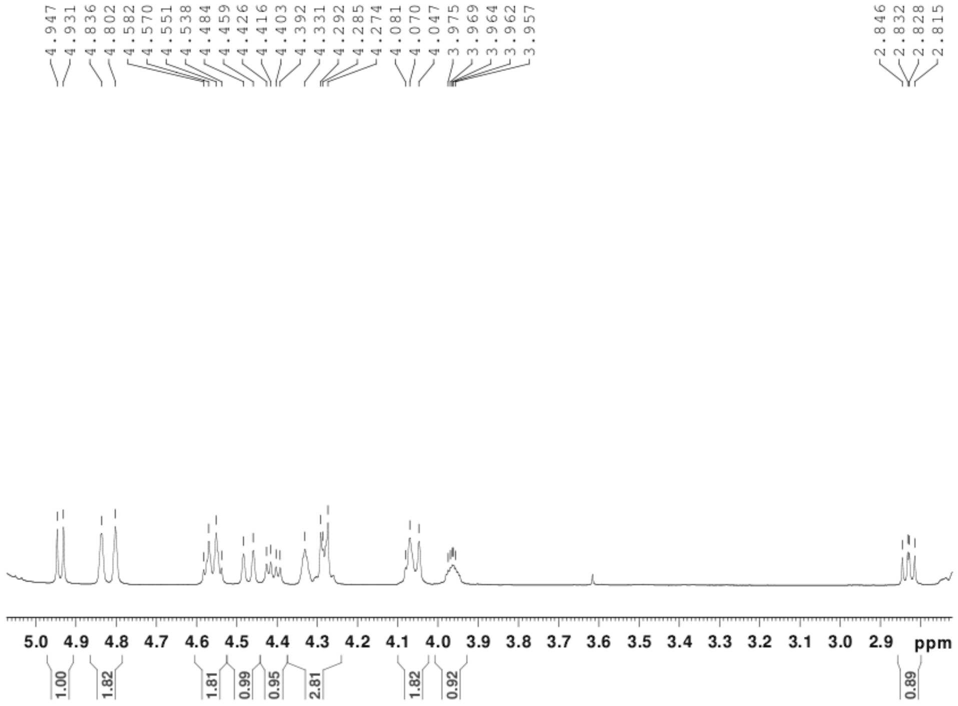

S44. Enlarged ¹H NMR (500 MHz, C_5D_5N) spectrum 1 for **6**.

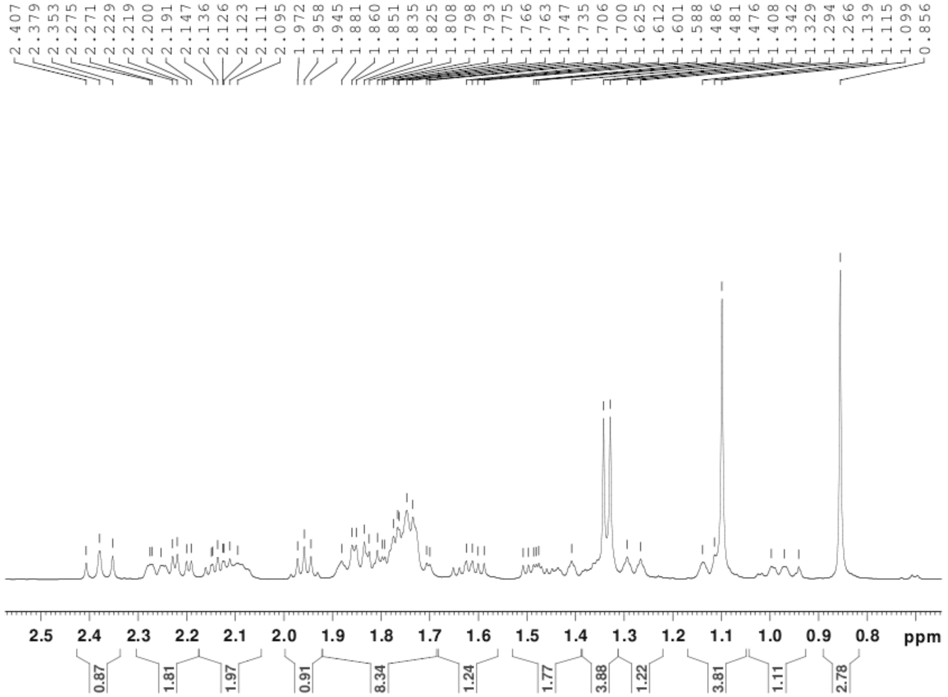

S45. Enlarged ¹H NMR (500 MHz, C_5D_5N) spectrum 2 for **6**.

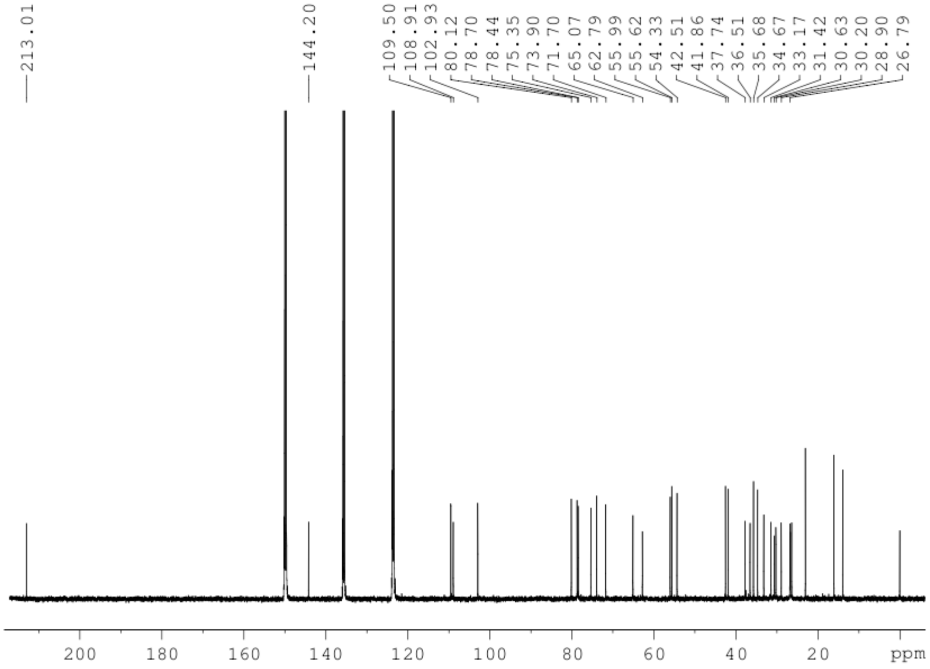

S46. ^{13}C NMR (125 MHz, C_5D_5N) spectrum for **6**.

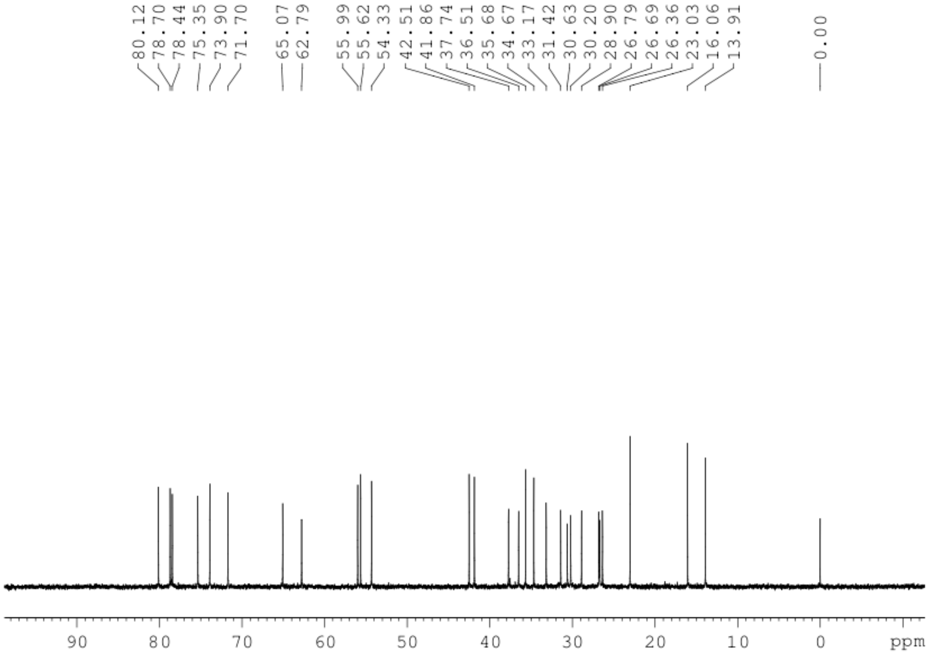

S47. Enlarged ^{13}C NMR (125 MHz, C_5D_5N) spectrum for **6**.

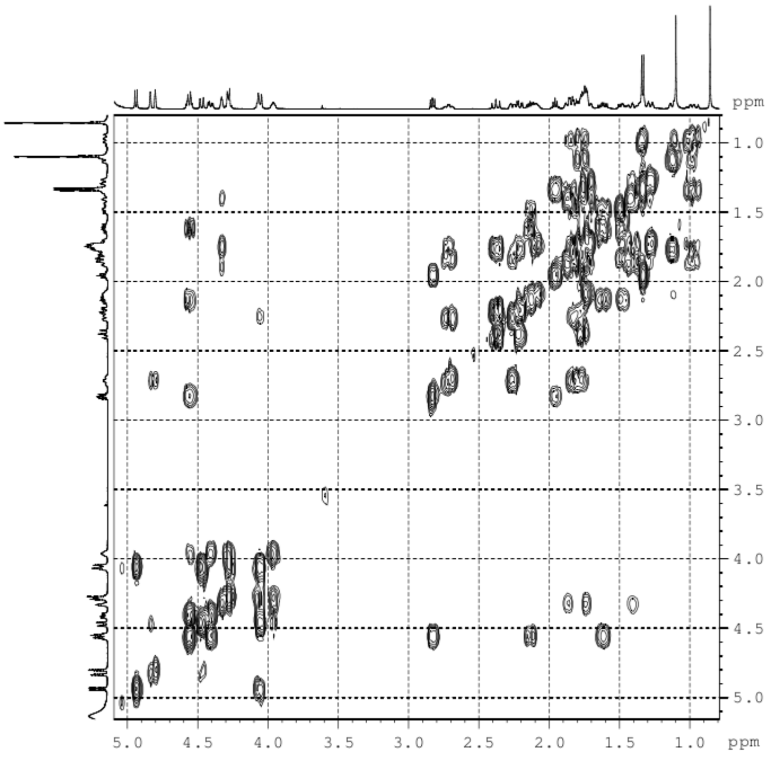

S48. ^{1}H ^{1}H COSY (C_5D_5N) spectrum for **6**.

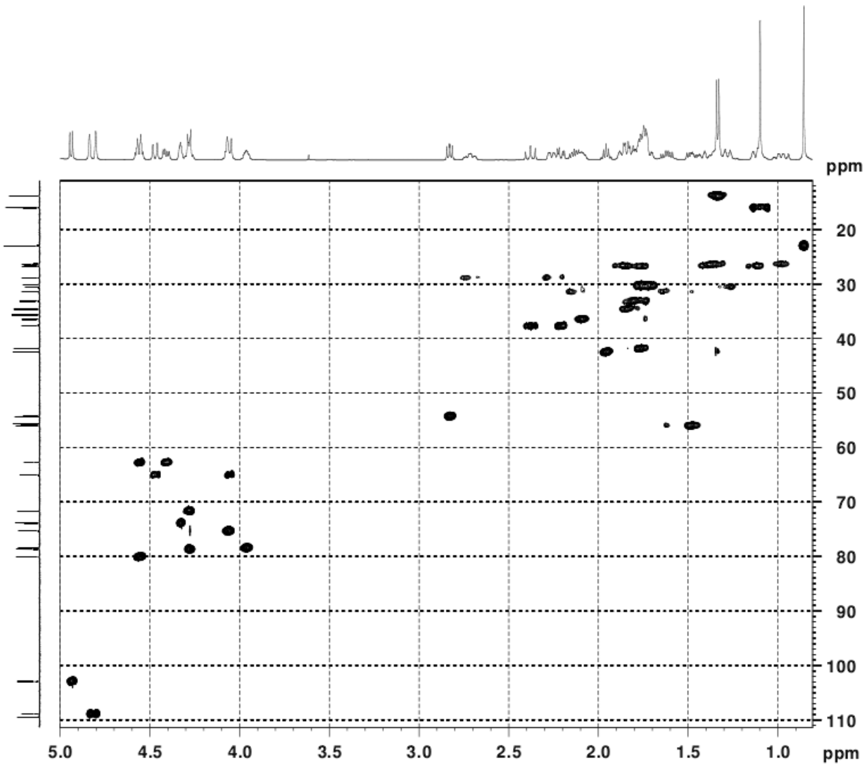

S49. HSQC (C_5D_5N) spectrum for **6**.

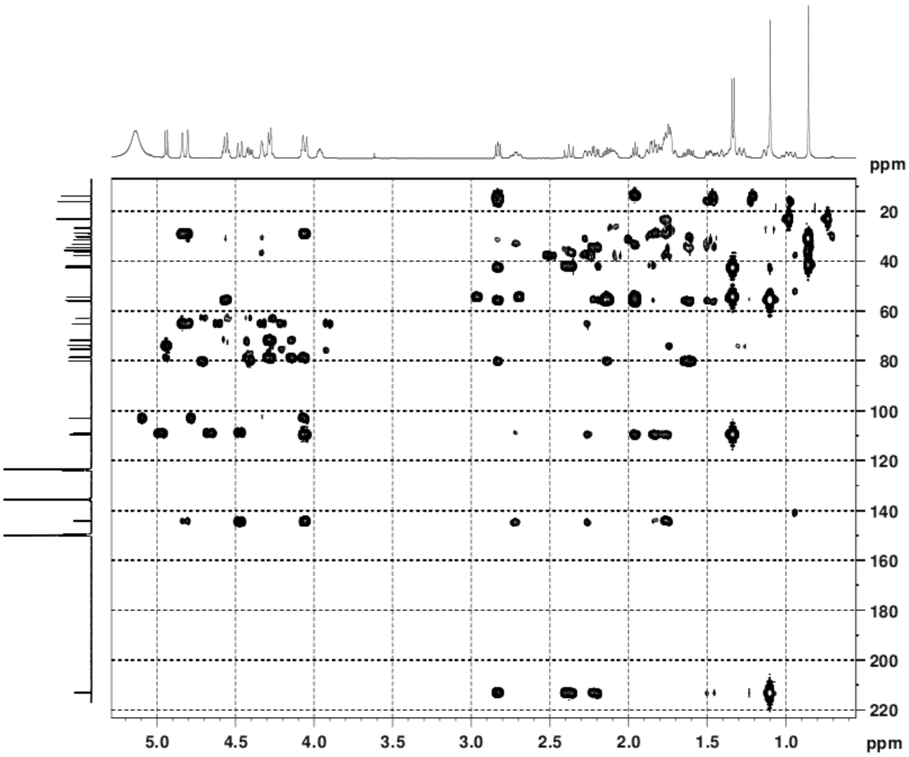

S50. HMBC (C_5D_5N) spectrum for **6**.

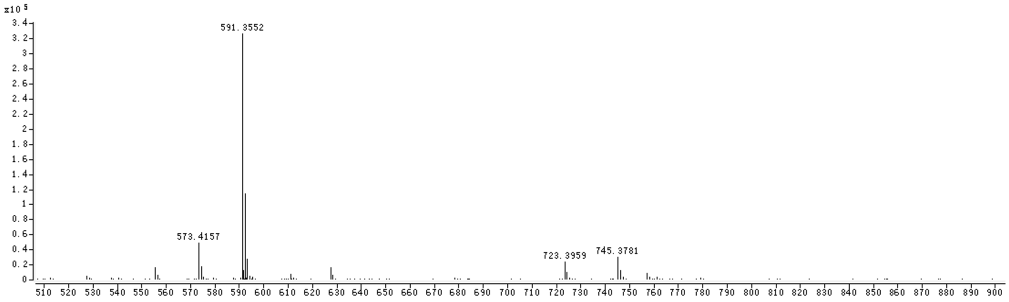

S51. HRESI-TOF-MS spectrum for **7**.

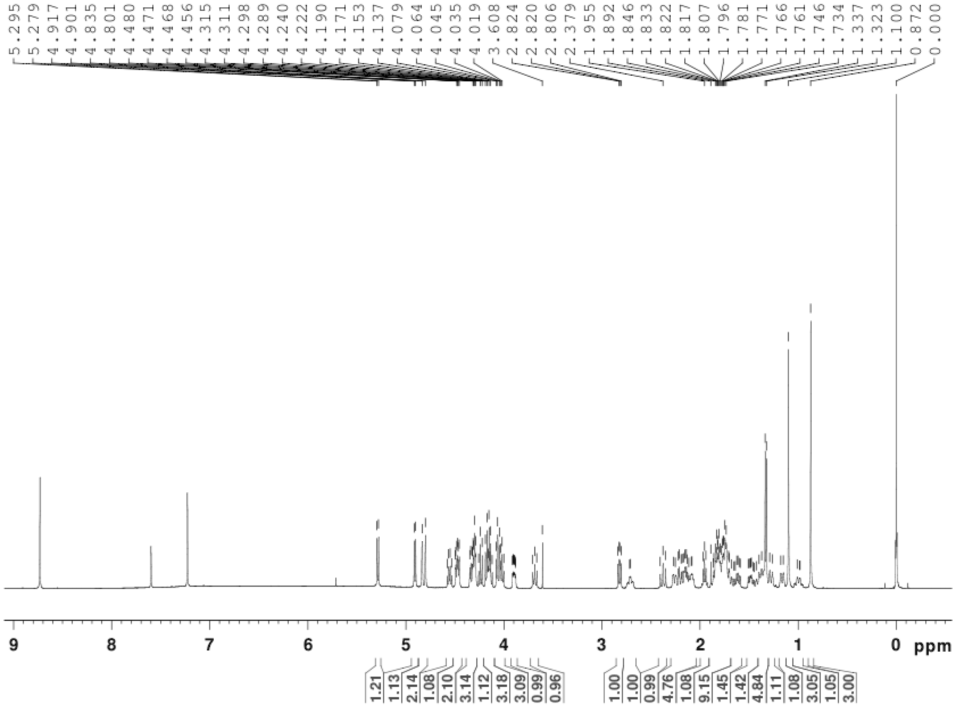

S52. ^{1}H NMR (500 MHz, C_5D_5N) spectrum for **7**.

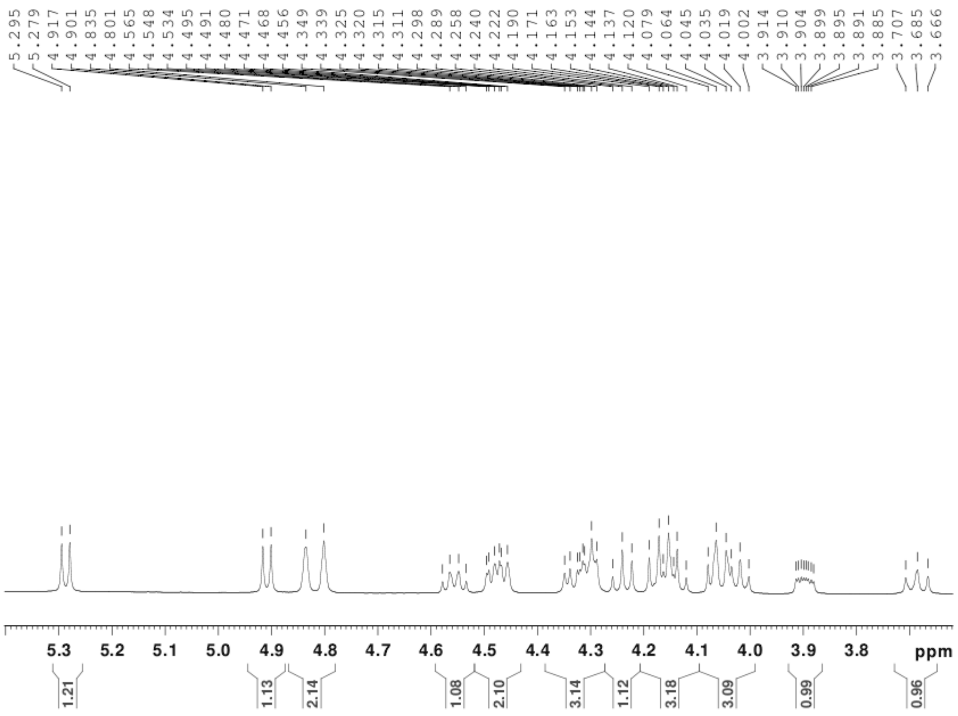

S53. Enlarged ^{1}H NMR (500 MHz, C_5D_5N) spectrum 1 for **7**.

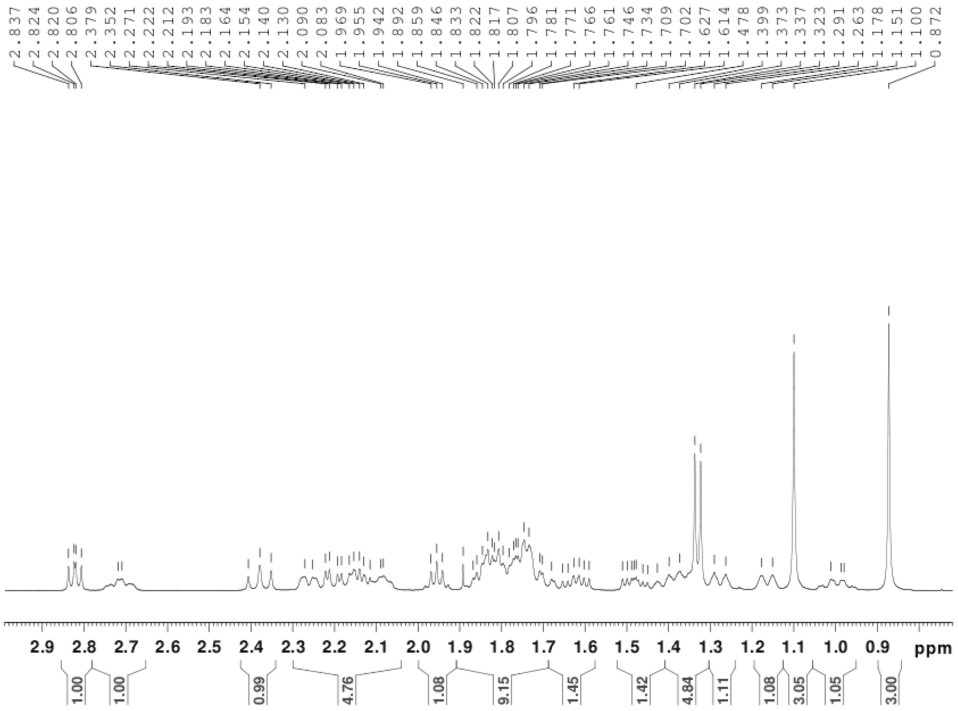

S54. Enlarged ^{1}H NMR (500 MHz, C_5D_5N) spectrum 2 for **7**.

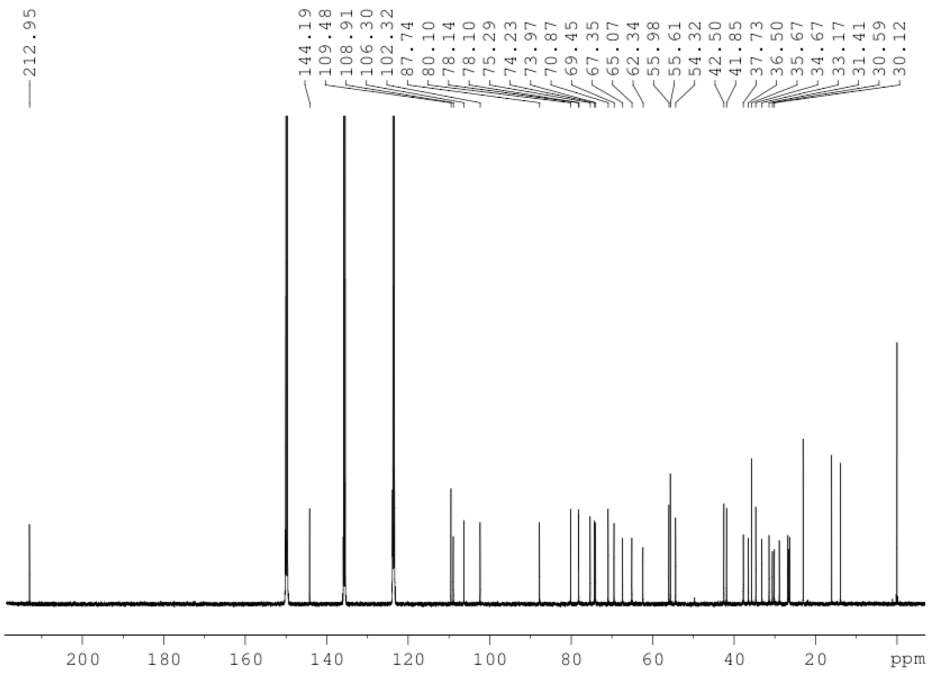

S55. ^{13}C NMR (125 MHz, C_5D_5N) spectrum for **7**.

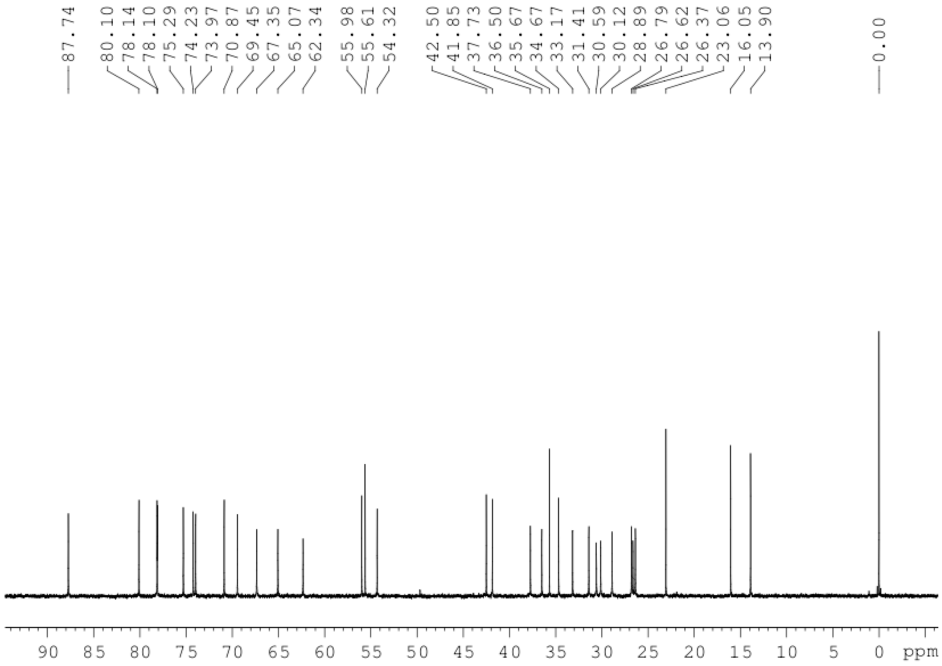

S56. Enlarged ^{13}C NMR (125 MHz, C_5D_5N) spectrum for **7**.

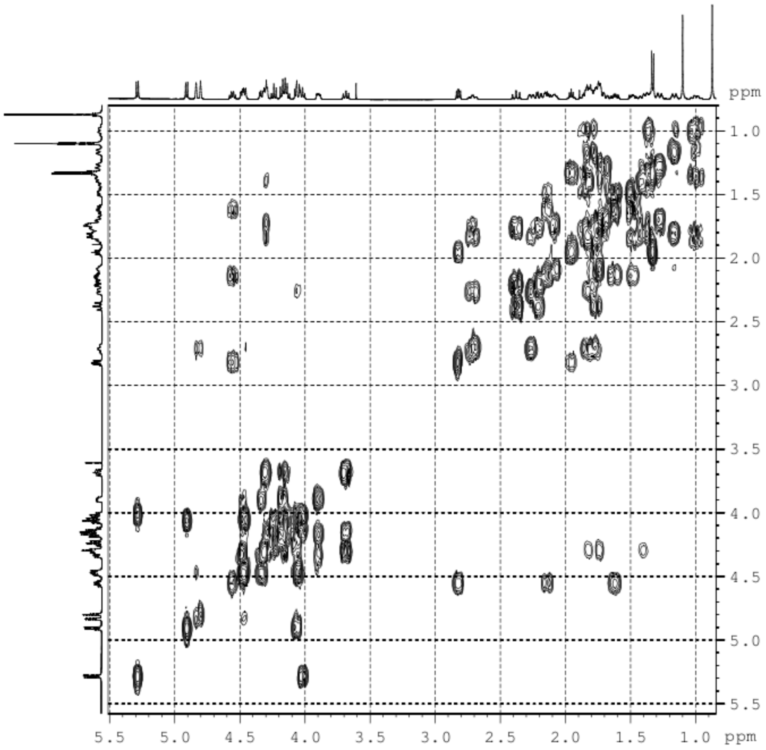

S57. ^{1}H ^{1}H COSY (C_5D_5N) spectrum for **7**.

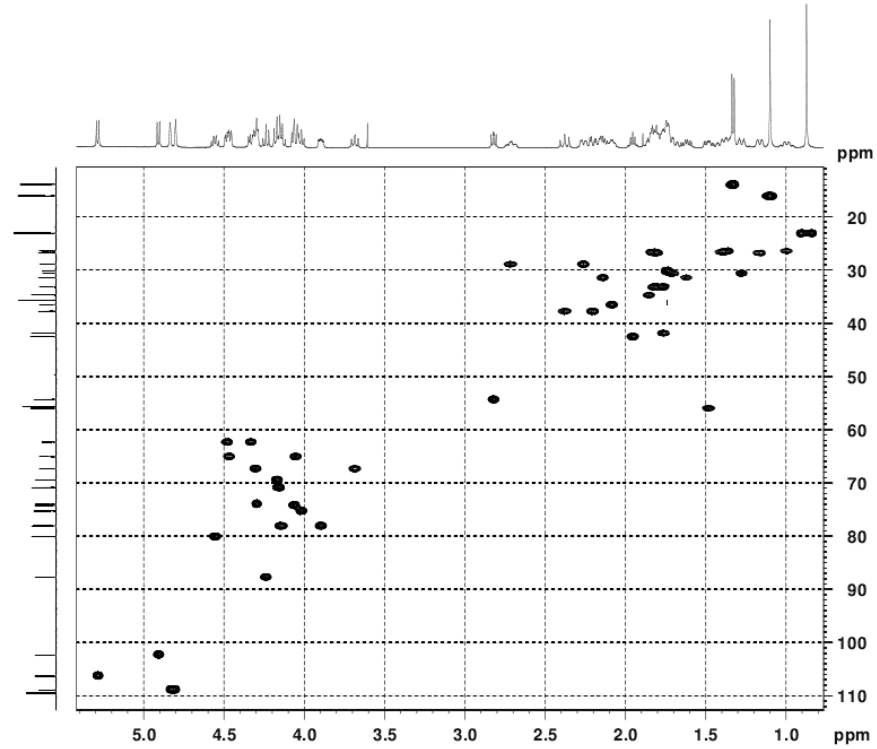

S58. HSQC (C_5D_5N) spectrum for **7**.

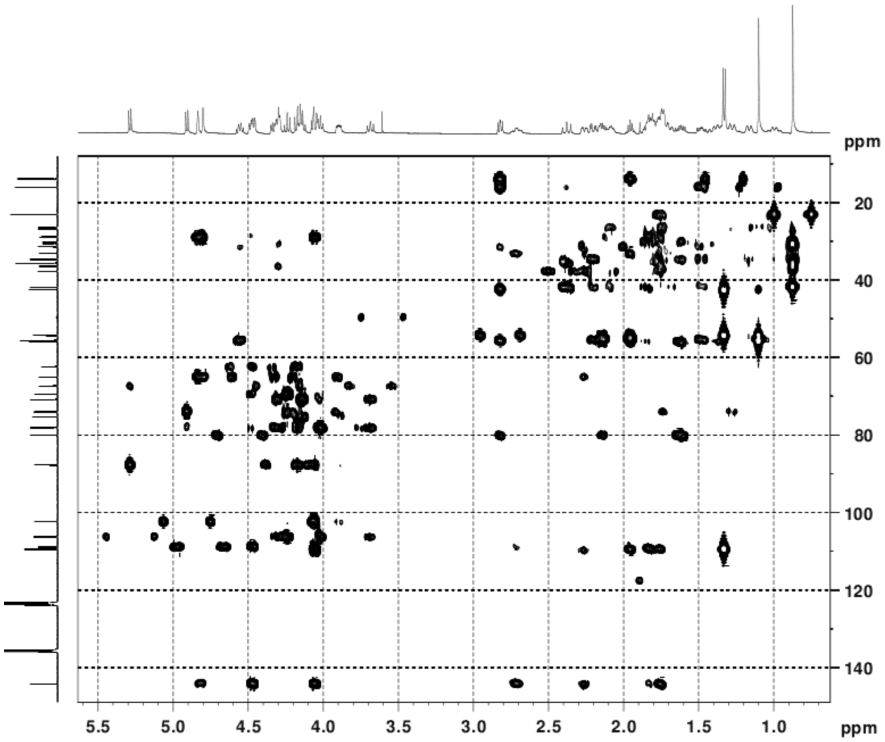

S59. HMBC (C_5D_5N) spectrum for **7**.

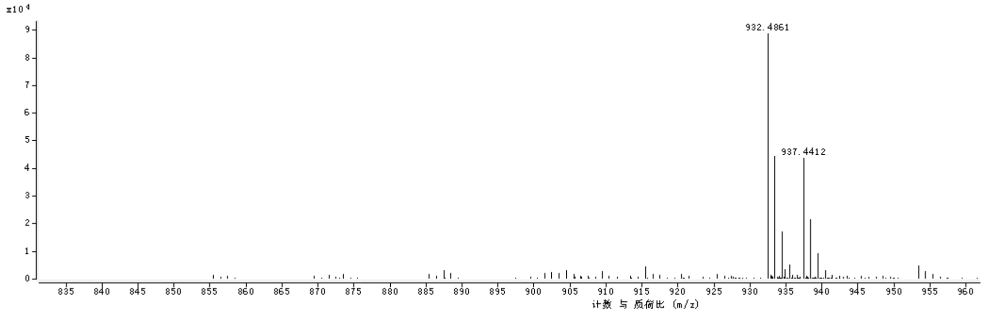

S60. HRESI-TOF-MS spectrum for **8**.

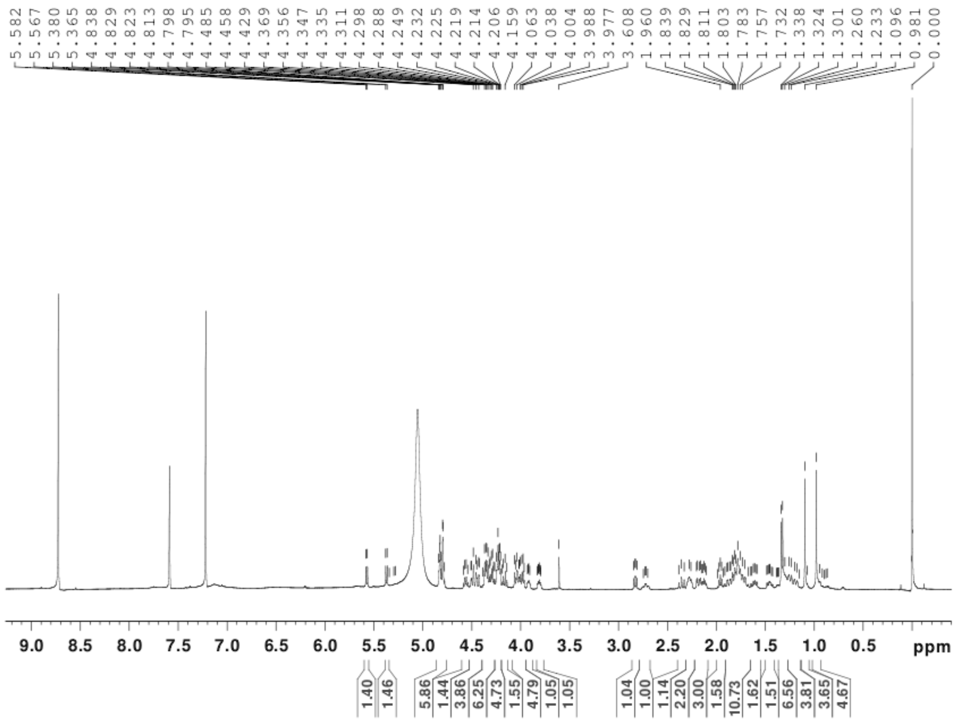

S61. ^{1}H NMR (500 MHz, C_5D_5N) spectrum for **8**.

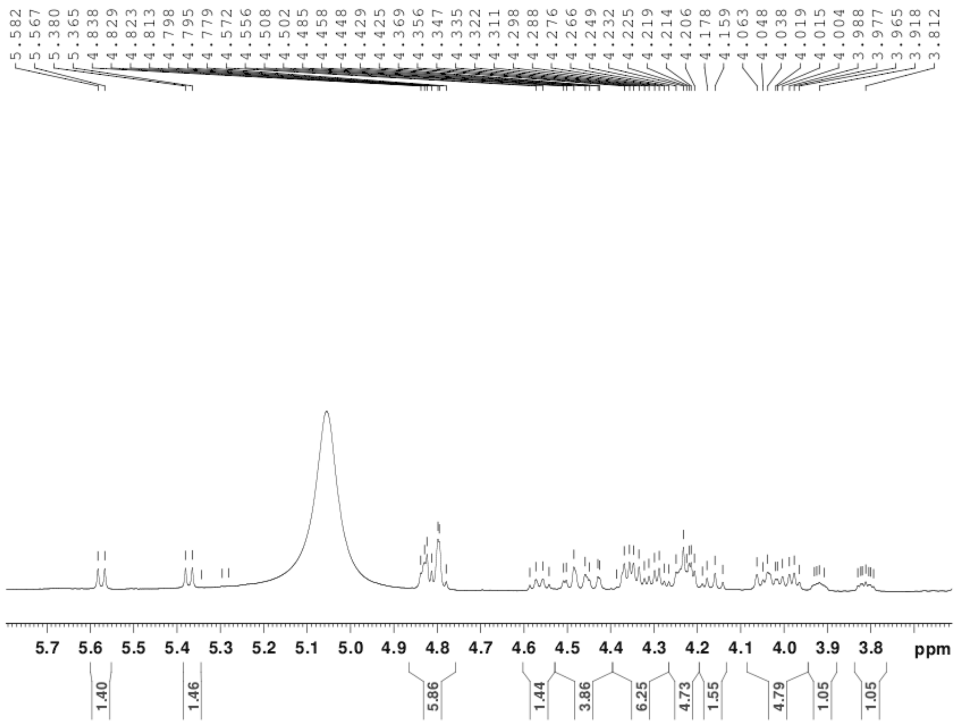

S62. Enlarged ^{1}H NMR (500 MHz, C_5D_5N) spectrum 1 for **8**.

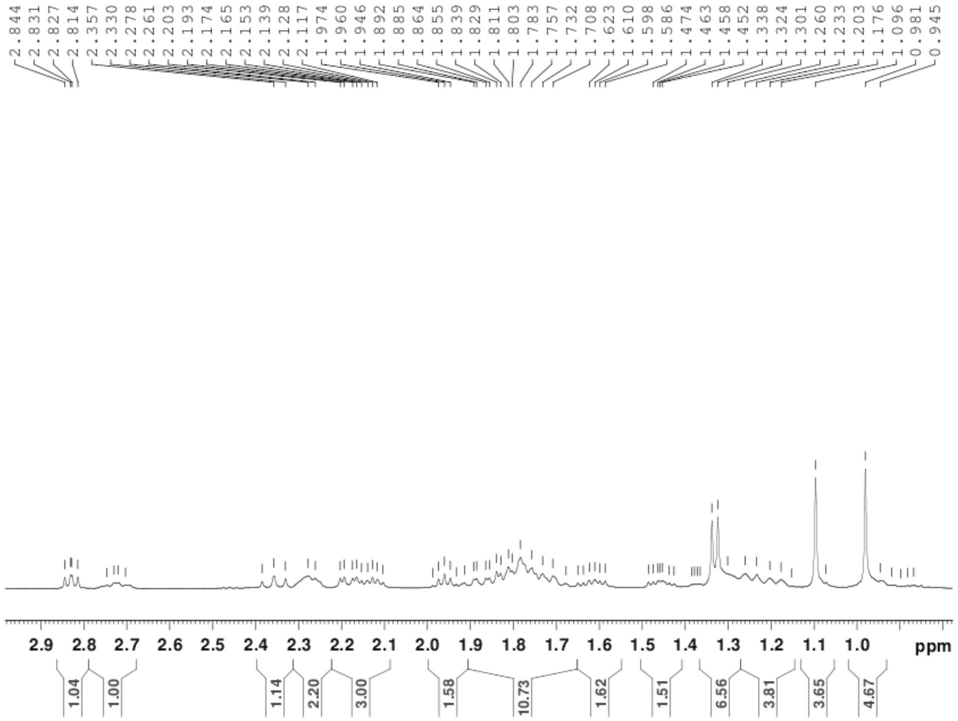

S63. Enlarged ^{1}H NMR (500 MHz, C_5D_5N) spectrum 2 for **8**.

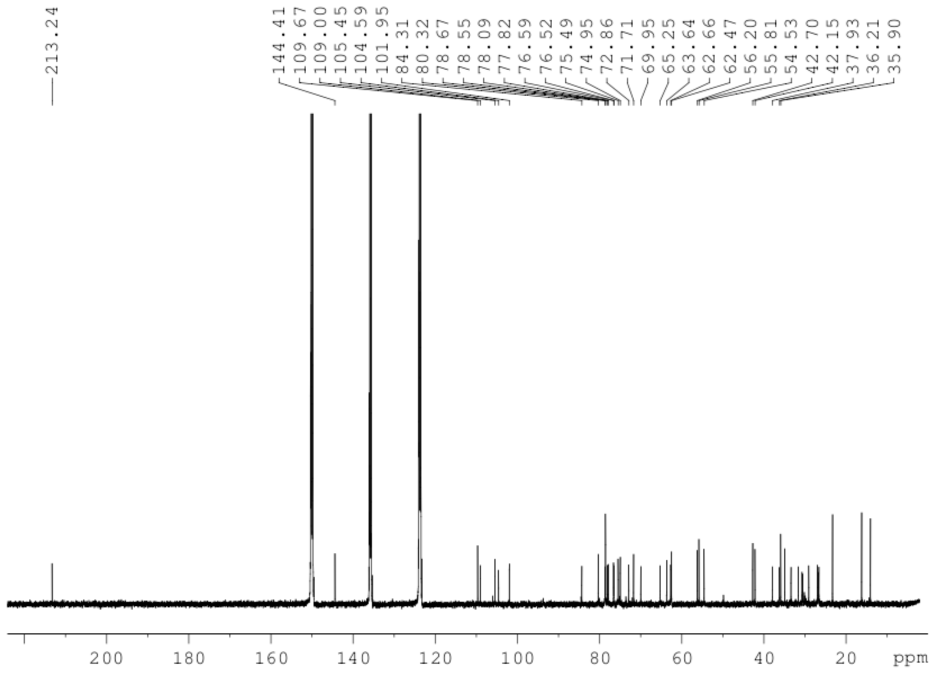

S64. ^{13}C NMR (125 MHz, C_5D_5N) spectrum for **8**.

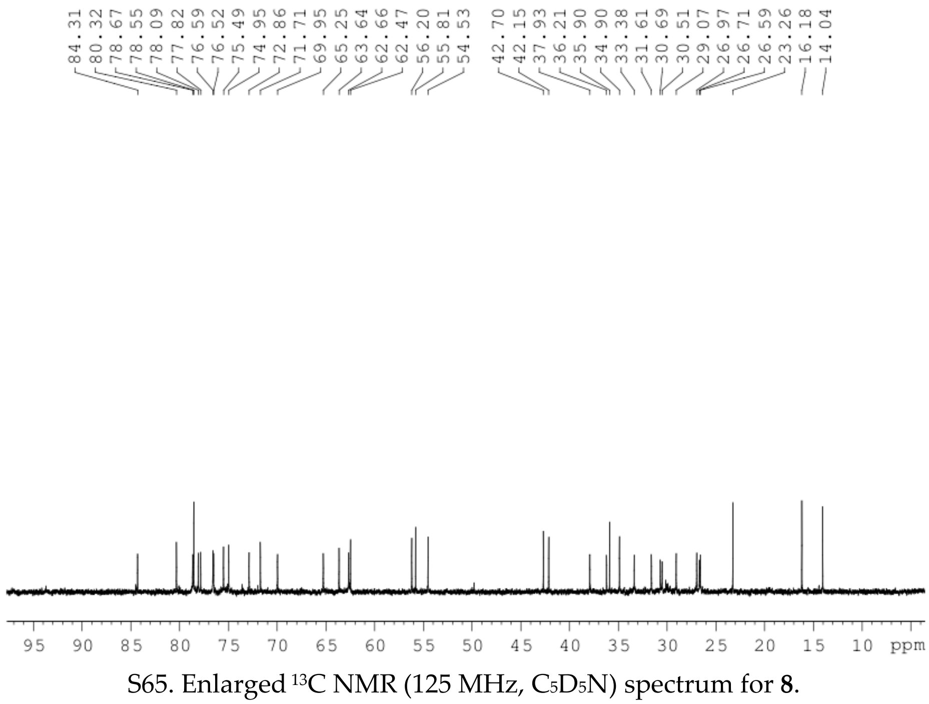

S65. Enlarged ^{13}C NMR (125 MHz, C_5D_5N) spectrum for **8**.

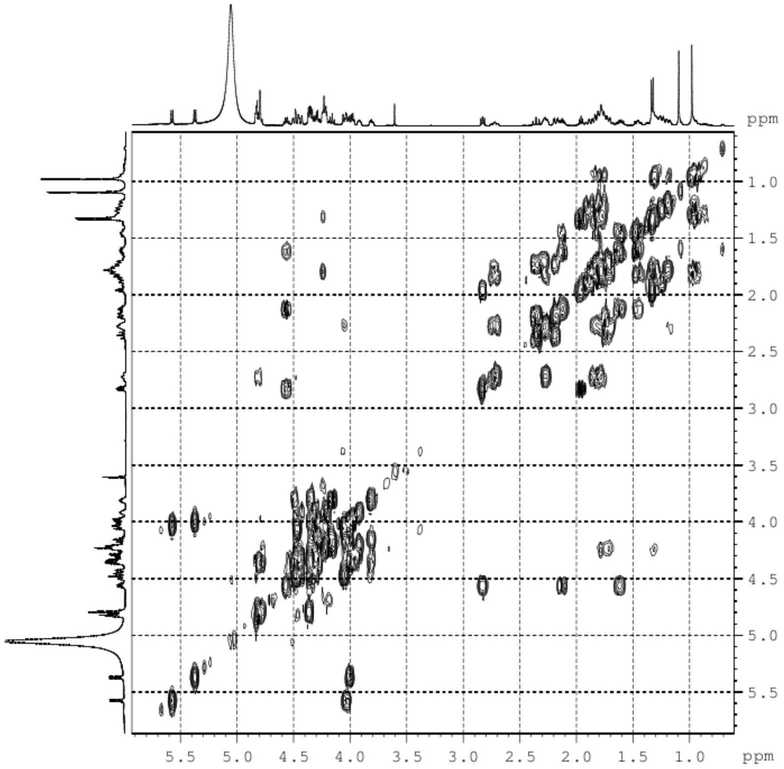

S66. ^{1}H ^{1}H COSY (C_5D_5N) spectrum for **8**.

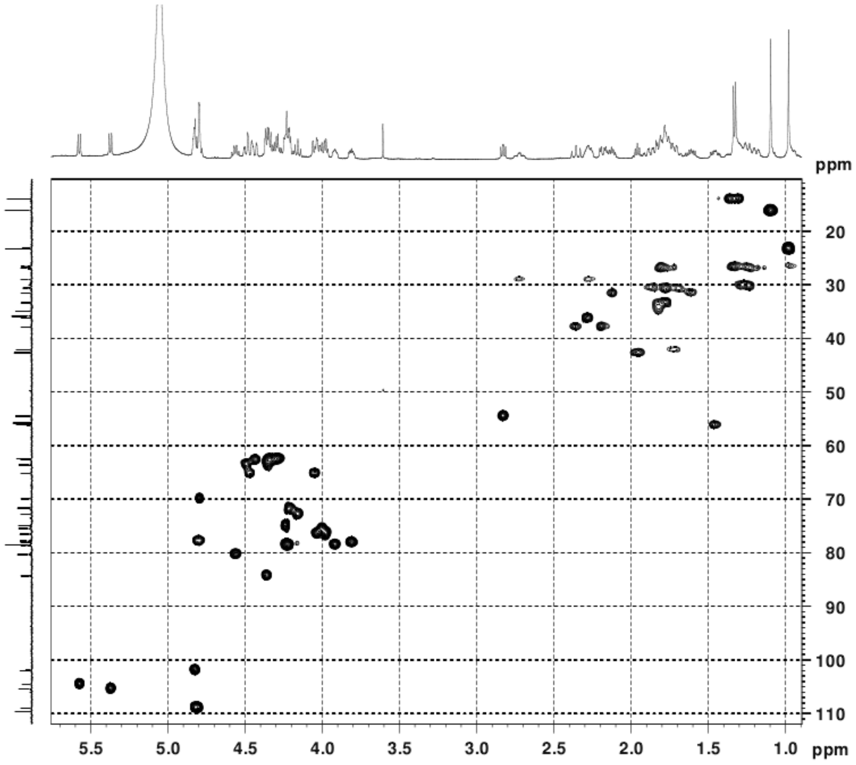

S67. HSQC (C_5D_5N) spectrum for **8**.

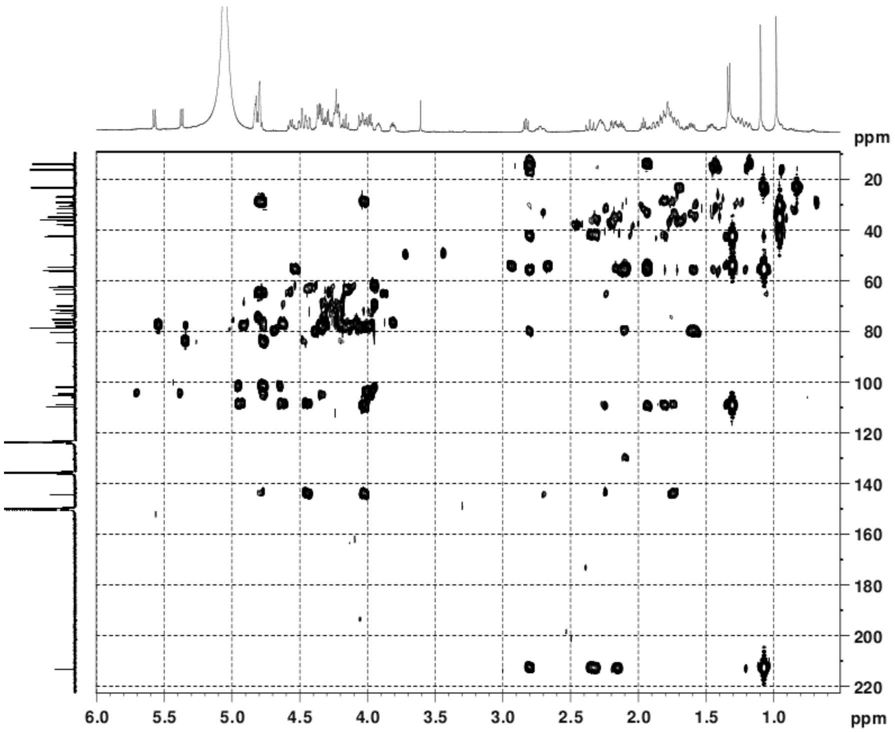

S68. HMBC (C_5D_5N) spectrum for **8**.

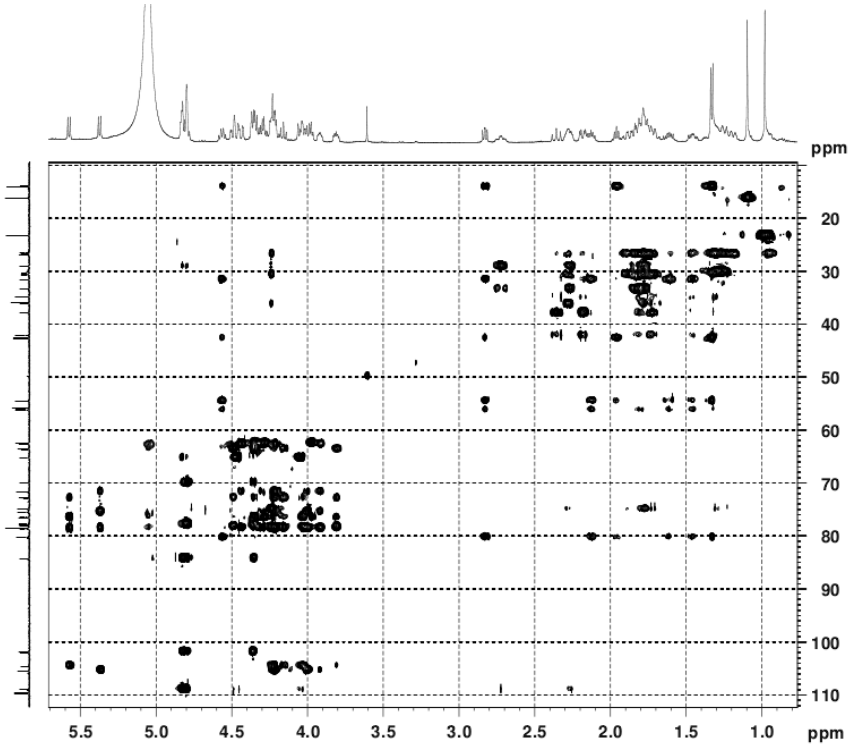

S69. HSQC-TOCSY (C_5D_5N) spectrum for **8**.

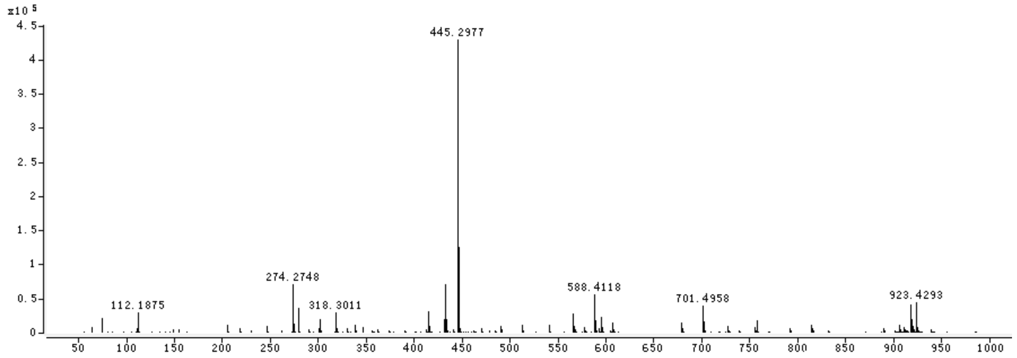

S70. HRESI-TOF-MS spectrum for **9**.

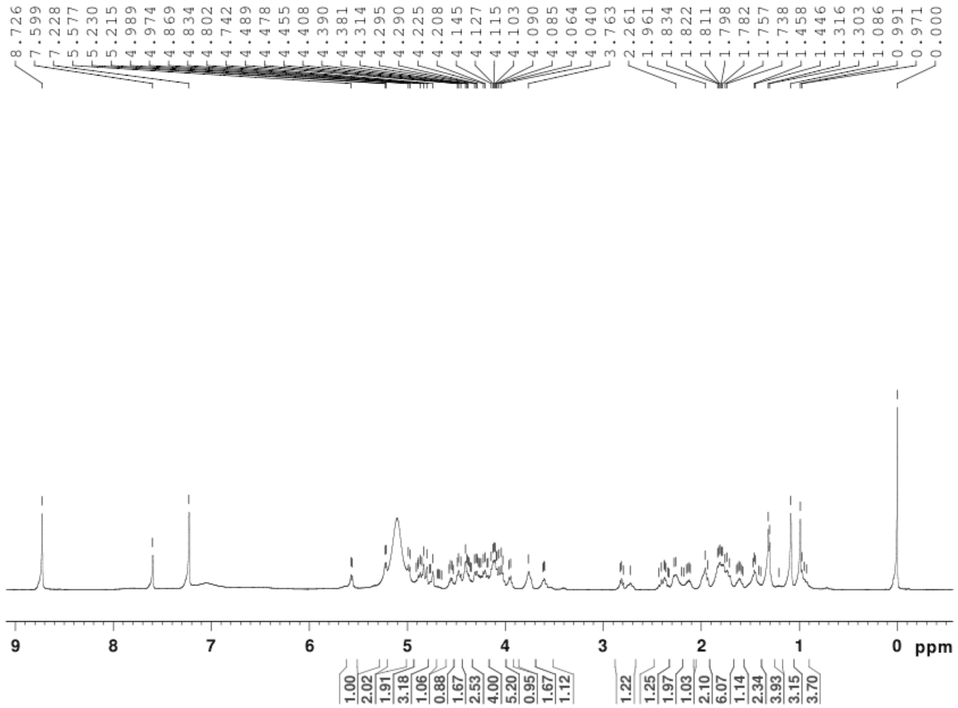

S71. ^{1}H NMR (500 MHz, C_5D_5N) spectrum for **9**.

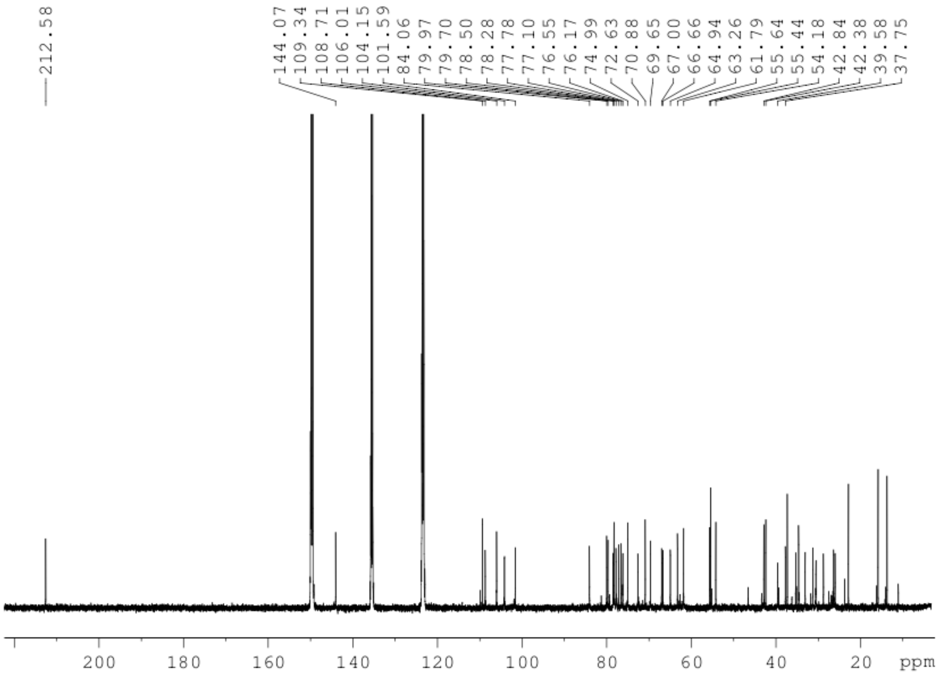

S72. ^{13}C NMR (125 MHz, C_5D_5N) spectrum for **9**.

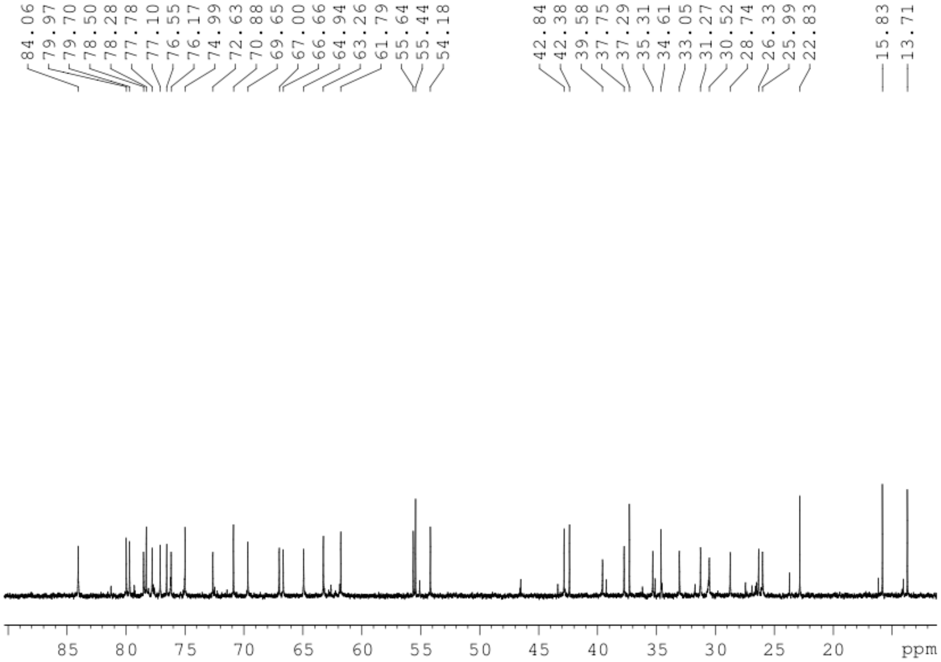

S73. Enlarged ^{13}C NMR (125 MHz, C_5D_5N) spectrum for **9**.

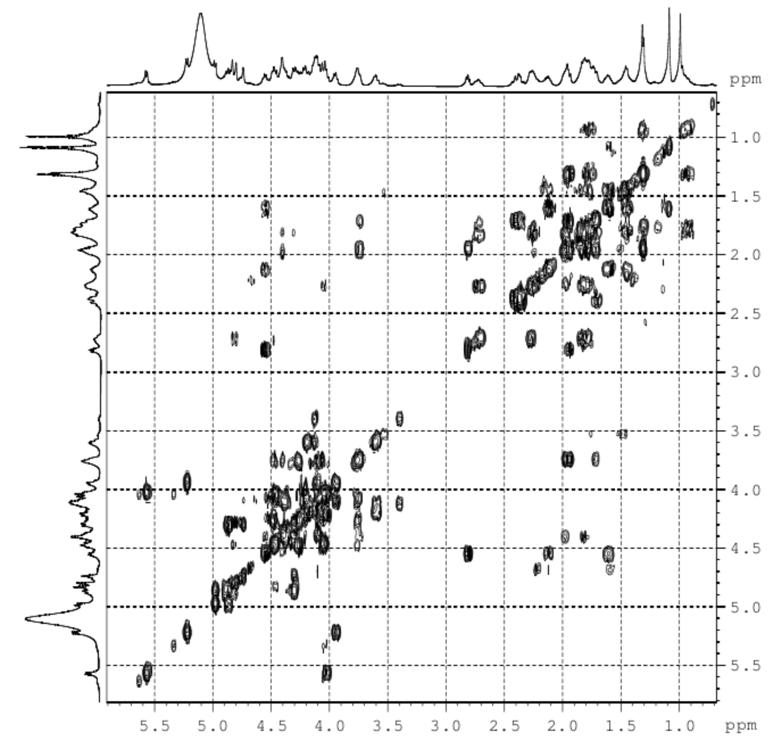

S74. ^{1}H ^{1}H COSY (C_5D_5N) spectrum for **9**.

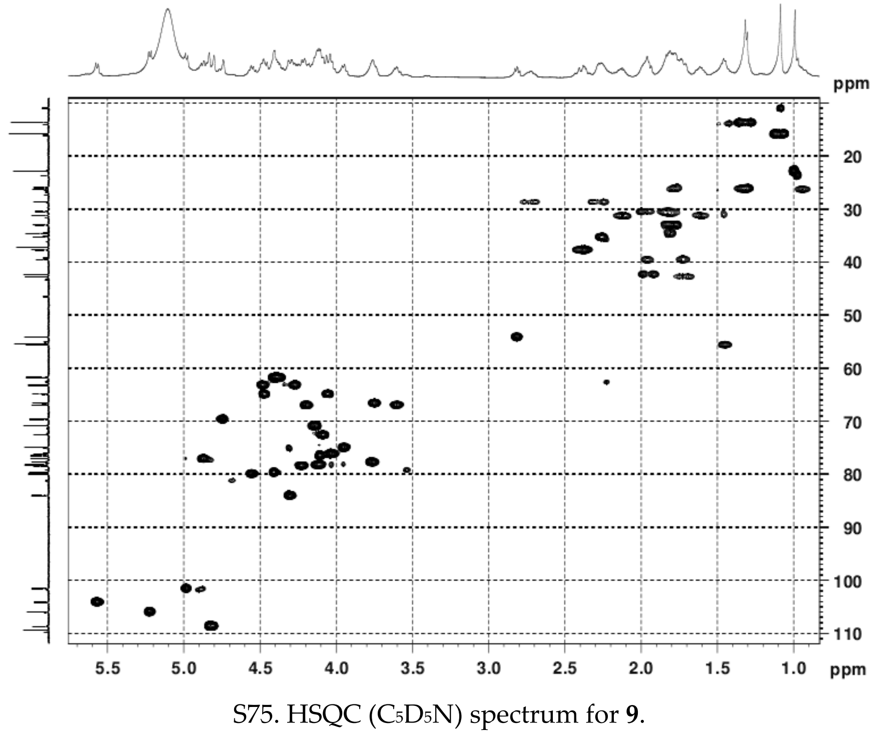

S75. HSQC (C_5D_5N) spectrum for **9**.

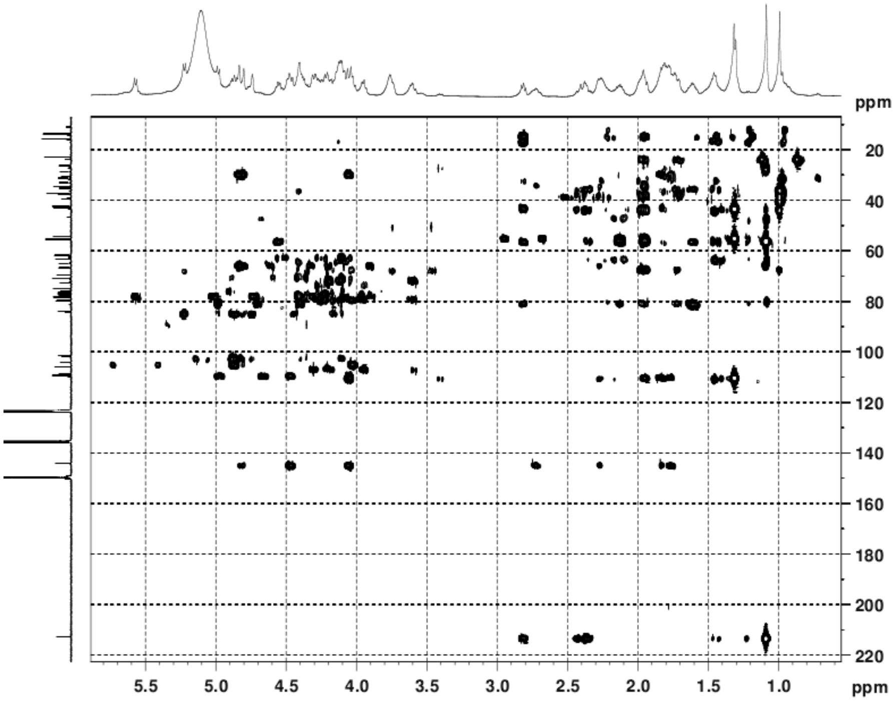

S76. HMBC (C_5D_5N) spectrum for **9**.

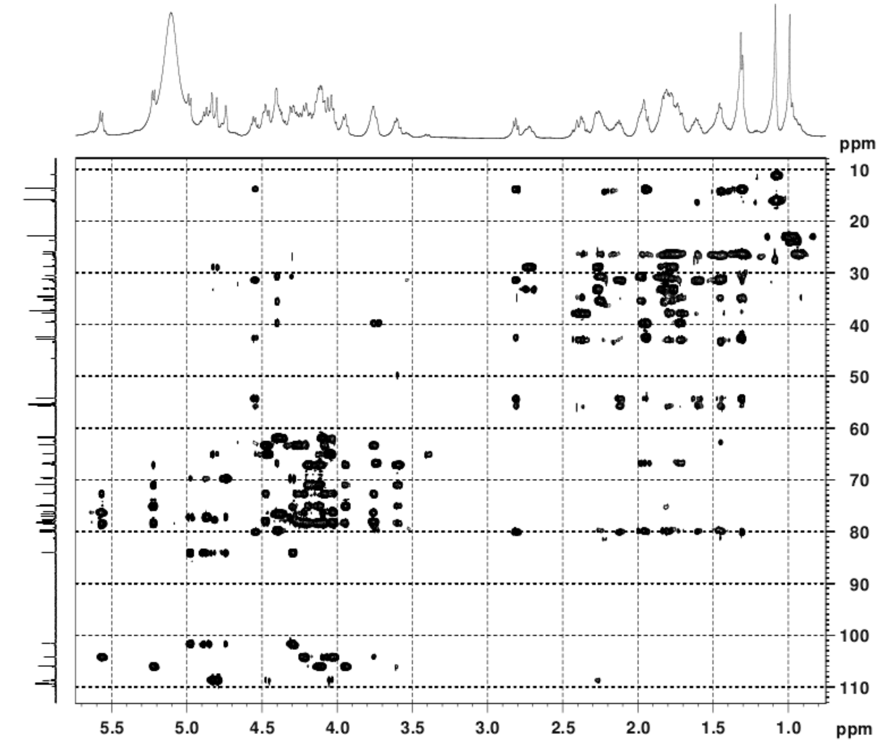

S77. HSQC-TOCSY (C_5D_5N) spectrum for **9**.
